# Selective cobalt nanoparticles for catalytic transfer hydrogenation of N-heteroarenes†
†Electronic supplementary information (ESI) available. See DOI: 10.1039/c7sc02062g



**DOI:** 10.1039/c7sc02062g

**Published:** 2017-07-12

**Authors:** Feng Chen, Basudev Sahoo, Carsten Kreyenschulte, Henrik Lund, Min Zeng, Lin He, Kathrin Junge, Matthias Beller

**Affiliations:** a Leibniz-Institut für Katalyse e.V. an der Universität Rostock , Albert-Einstein Straße 29a , Rostock , 18059 , Germany . Email: matthias.beller@catalysis.de; b State Key Laboratory for Oxo Synthesis and Selective Oxidation , Suzhou Research Institute of LICP , Chinese Academy of Sci-ences , Lanzhou 730000 , P. R. China

## Abstract

Nitrogen modified cobalt catalysts supported on carbon were prepared by pyrolysis of the mixture generated from cobalt(ii) acetate in aqueous solution of melamine or waste melamine resins, which are widely used as industrial polymers. The obtained nanostructured materials catalyze the transfer hydrogenation of N-heteroarenes with formic acid in the absence of base. The optimal Co/Melamine-2@C-700 catalyst exhibits high activity and selectivity for the dehydrogenation of formic acid into molecular hydrogen and carbon dioxide and allows for the reduction of diverse N-heteroarenes including substrates featuring sensitive functional groups.

## Abstract 2

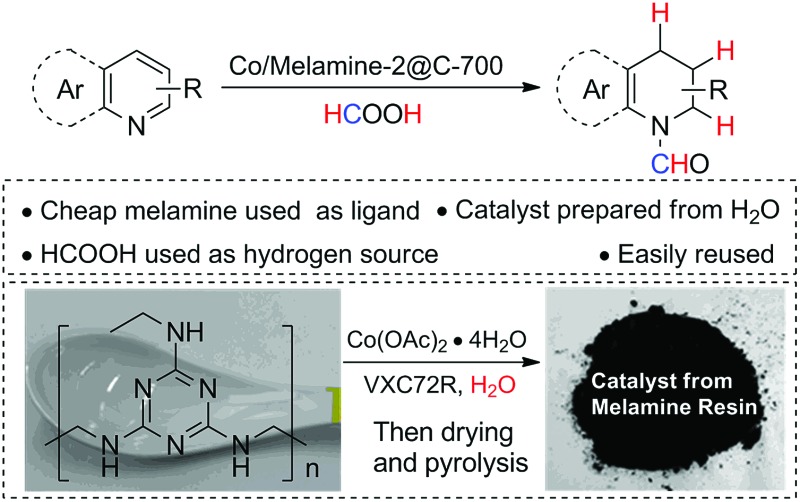
Nitrogen modified cobalt nanoparticles are easily prepared from melamine or melamine resins. The resulting catalysts show excellent selectivity for transfer hydrogenation of N-heteroarenes.

## Introduction

Transfer hydrogenation is a fundamental transformation for organic synthesis, which is applied widely both in academic and industrial laboratories. For this transformation, besides well-developed organometallic complexes,^1^ heterogeneous materials constitute attractive catalysts because of their easy handling, separation, and reusability. Most of these known systems are based on precious metals, *e.g.* Pd, Ru, Ir, Rh, Au, and Ag, although also Ni-based catalysts have been reported.^1^ In general, transfer hydrogenations can be conveniently carried out without pressure equipment,^1,2^ and make use of isopropanol or formic acid derivatives as “liquid hydrogen donors”. Interestingly, formic acid can be produced from biomass as an environmental friendly and efficient reductant. In recent years, several improved heterogeneous as well as homogeneous catalysts have been developed for the dehydrogenation of HCOOH, especially in the context of hydrogen storage.^3^

Among the known transfer hydrogenation of different functional groups, the selective reduction of N-heteroarenes is challenging, because of their high resonance stability^4^ and the possibility of poisonous effects towards the catalyst. In fact, such substrates have been much less investigated compared to the more common catalytic hydrogenations.^5^ A recent example of transfer hydrogenations of quinolines – the resulting products constitute a common motif in natural products and bioactive molecules^4*c*^ – includes the report by Cao and co-workers using gold nanoparticles supported on titanium (Au/TiO_2_-R).^6^ Besides that, Pd/C was also reported by Török and co-workers for related reactions.^7^ Clearly, the development of more abundant non-noble metal based catalysts has been rarely explored for such transformations so far.

Recently, we^8^ and other groups^9^ developed new materials in which non-noble metal nanoparticles cooperatively interact with N-doped graphene and graphitic layers.^10^ Due to this specific environment, these materials show excellent selectivity in several hydrogenation and oxidation reactions. Unfortunately, most of these catalysts require more special amine ligands, *e.g.* phenanthroline, for their synthesis. Hence, there is ongoing interest to generate such materials with less expensive N-sources.^11^ Herein, we report a more convenient and inexpensive synthesis of cobalt nanoparticles supported on nitrogen modified carbon and their application in the selective transfer hydrogenation of N-heteroarenes.

**Scheme 1:**
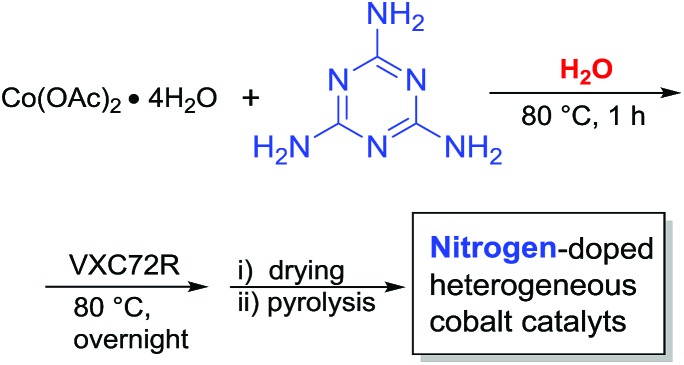
Preparation of cobalt nanoparticles from melamine in water.

Melamine is an important industrial building block and monomer for paints, plastics, paper products and so on.^12^ It is very economical and contains 67% nitrogen by mass. Thus, it is an ideal nitrogen source for the preparation of N-modified carbon materials.^13^ Based on this and our previous work, we thought, it would allow the preparation of novel heterogenized metal nanoparticles. Initially, a series of cobalt catalysts was prepared by mixing cobalt acetate with melamine in water, addition of the support (Vulcan VXC72R) and subsequent pyrolysis at different temperatures (400–800 °C for 2 h under argon atmosphere) (Scheme 1).

## Results and discussion

The obtained materials were tested for the transfer hydrogenation of quinoline (**1a**) as a model substrate for N-heteroarenes. Using a combination of HCOOH/Et_3_N (8 : 1), the catalyst obtained at 700 °C gave the best yield of 3,4-dihydroquinoline-1(2*H*)-carbaldehyde (**2a**), although the materials produced at 600 and 800 °C also exhibited high activity (Table 1, entries 1–3). However, a significantly reduced conversion is observed in the presence of the material prepared at 400–500 °C (Table 1, entries 4 and 5). The material prepared without melamine gave **2a** only in 15% yield (Table 1, entry 7). This implies that the doping with nitrogen is crucial to achieve sufficient activity for this transformation. Obviously, the material prepared without cobalt precursor is inactive (Table 1, entry 8). Next, we were surprised to discover full conversion with 98% yield of **2a** without any presence of base (TEA) (Table 1, entry 9). It should be noted at this point that most transfer hydrogenation processes require addition of stoichiometric amount of base, which limits the practicability of such methods. Using different amounts of HCOOH for this transformation showed that 6 equivalents of HCOOH are necessary to obtain excellent yields (Table 1, entries 10–14). Besides, other reaction parameters were also investigated and the best reaction conditions are shown in Table 1, entry 15.

**Table 1:** Cobalt-catalyzed transfer hydrogenation of quinoline with formic acid^*a*^

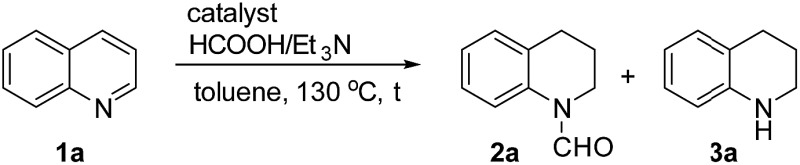						
Entry	Catalyst (mg)	HCOOH (eq.)	Et_3_N (eq.)	*t* (h)	Conv. (%)	Yield^*b*^ (%) **2a**
1	Co/Melamine-2@C-800 (60)	10	1.25	24	100	97
2	Co/Melamine-2@C-700 (60)	10	1.25	24	100	99
3	Co/Melamine-2@C-600 (60)	10	1.25	24	97	96
4	Co/Melamine-2@C-500 (60)	10	1.25	24	20	18
5	Co/Melamine-2@C-400 (60)	10	1.25	24	18	17
6	Co/Melamine-1@C-700 (60)	10	1.25	24	95	94
7	Co@C-800 (60)	10	1.25	24	16	15
8	Melamine-2@C-800 (60)	10	1.25	24	0	0
**9**	**Co/Melamine-2@C-700 (60)**	**10**	**—**	**24**	**100**	**98**
10	Co/Melamine-2@C-700 (60)	8	—	24	100	97
11	Co/Melamine-2@C-700 (60)	6	—	24	100	95
12	Co/Melamine-2@C-700 (60)	5	—	24	88	81
13	Co/Melamine-2@C-700 (60)	4	—	24	71	58
14	Co/Melamine-2@C-700 (60)	3	—	24	51	42
15	Co/Melamine-2@C-700 (60)	8	—	18	100	99
16	Co/Melamine-2@C-700 (60)	8	—	8	72	71

^*a*^0.5 mmol quinoline in 1.5 mL toluene.

^*b*^GC-yield using dodecane as an internal standard.

The element analysis of Co/Melamine-2@C-700 shows that it contains 5.0 wt% of cobalt and 0.75 wt% of nitrogen. The detailed characterizations of the selected materials have been investigated by scanning transmission electron microscopy (STEM), X-ray photoelectron spectroscopy (XPS) and Powder X-Ray Diffraction (PXRD).

**Fig. 1:**
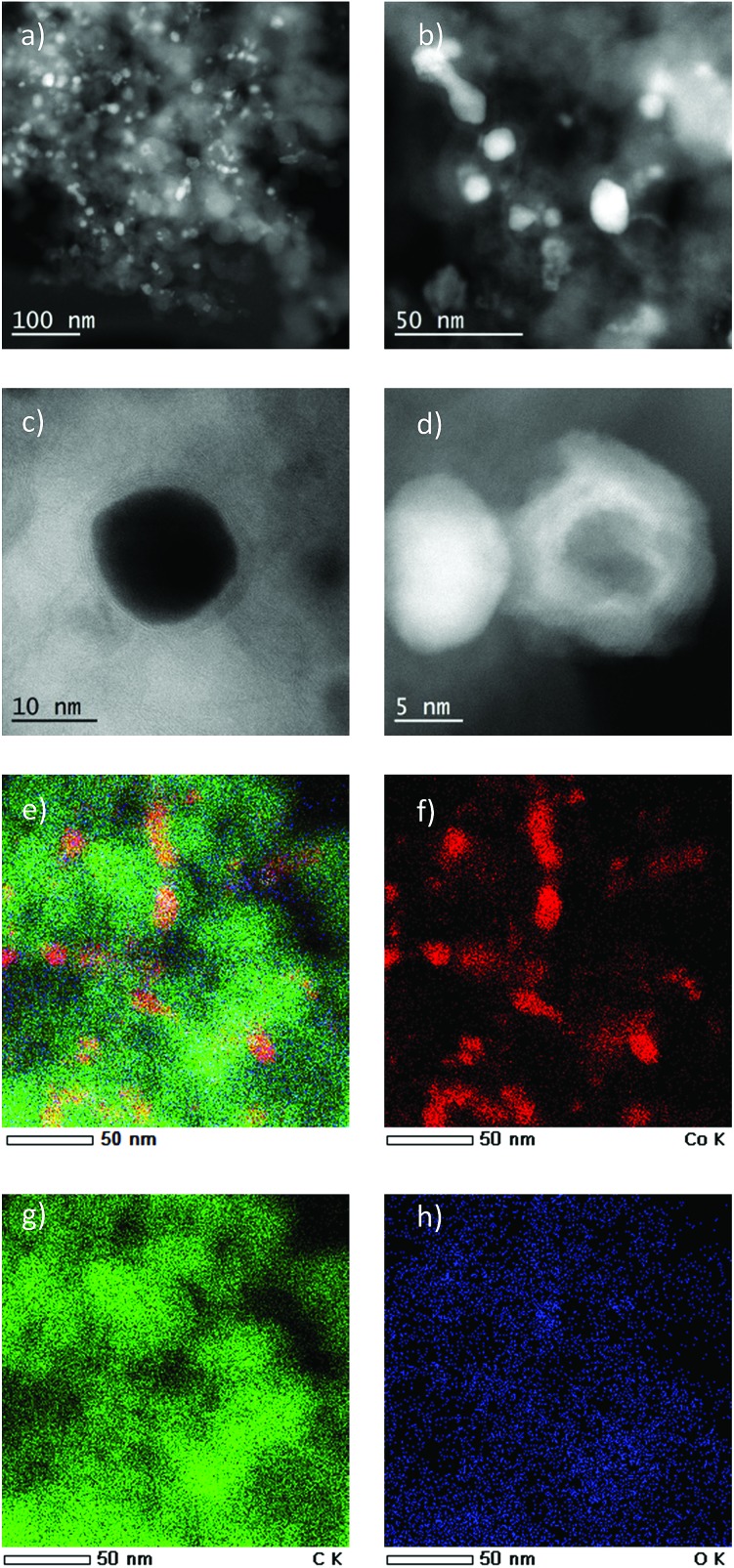
(a–c) STEM images of Co/Melamine-2@C-700. The high angle annular dark field (HAADF) image (a and b) of Co/Melamine-2@C-700 shows the distribution of metallic (dense contrast) and oxidic (cloudy, less intense) Co particle, while they close up (c) shows an example of both type next to each other. The annular bright field (ABF) image (d) displays a close up of a metallic cobalt particle surrounded by a thin graphitic structure. (e–h) STEM-EDX mapping of another catalyst region showing the carbon/cobalt structure present in the fresh specimen: (e) overlay of cobalt (red), carbon (green) and oxygen (blue) maps, (f) cobalt map, (g) carbon map, (h) oxygen map.

**Fig. 2:**
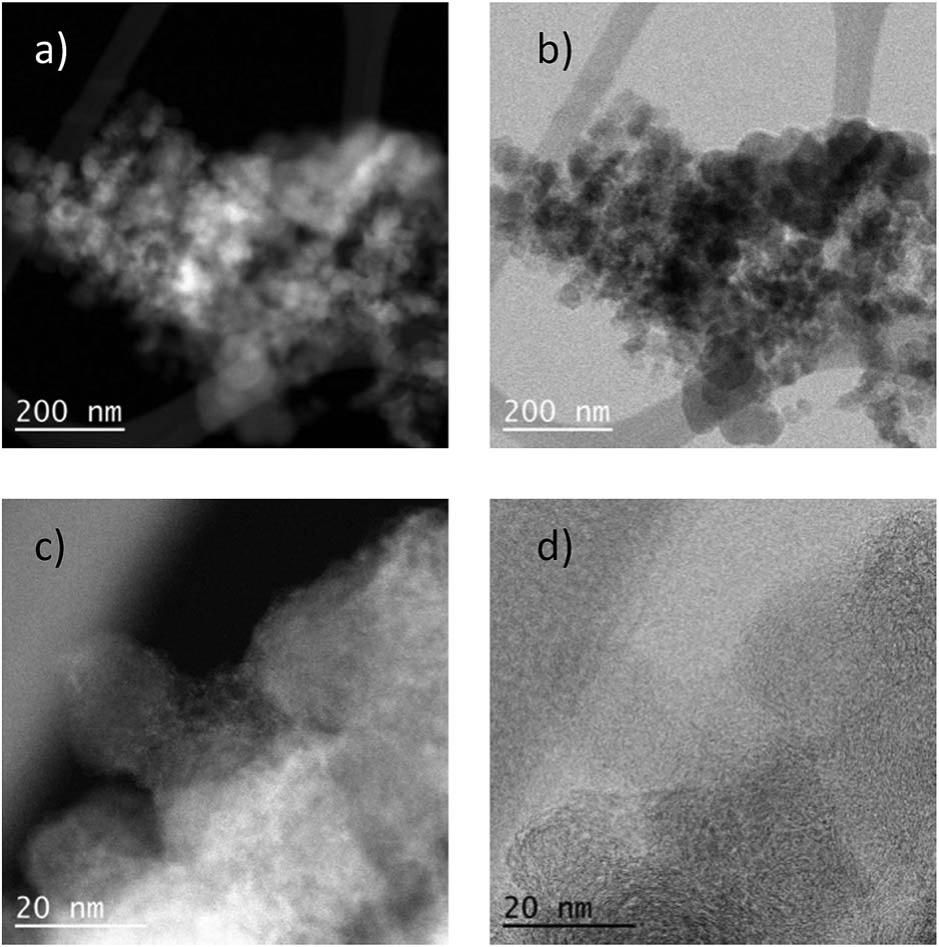
STEM images of Co/Melamine-2@C-400 (a) high angle annular dark field (HAADF) overview of Co/Melamine-2@C-400, (b) annular bright field (ABF) image of Co/Melamine-2@C-400, no cobalt particles were observed, (c and d) HAADF and ABF images showing details of the carbon phase of Co/Melamine-2@C-400.

The most active Co/Melamine-2@C-700 and less active Co/Melamine-2@C-400 were compared by STEM. In the first sample different kinds of cobalt species were detected: in addition to metallic cobalt particles surrounded by graphitic layers, cobalt oxide particles without graphitic structures are present. Most particle sizes are less than 20 nm (Fig. 1). No cobalt particles were detected for the material pyrolyzed at 400 °C (Co/Melamine-2@C-400) (Fig. 2).

**Fig. 3:**
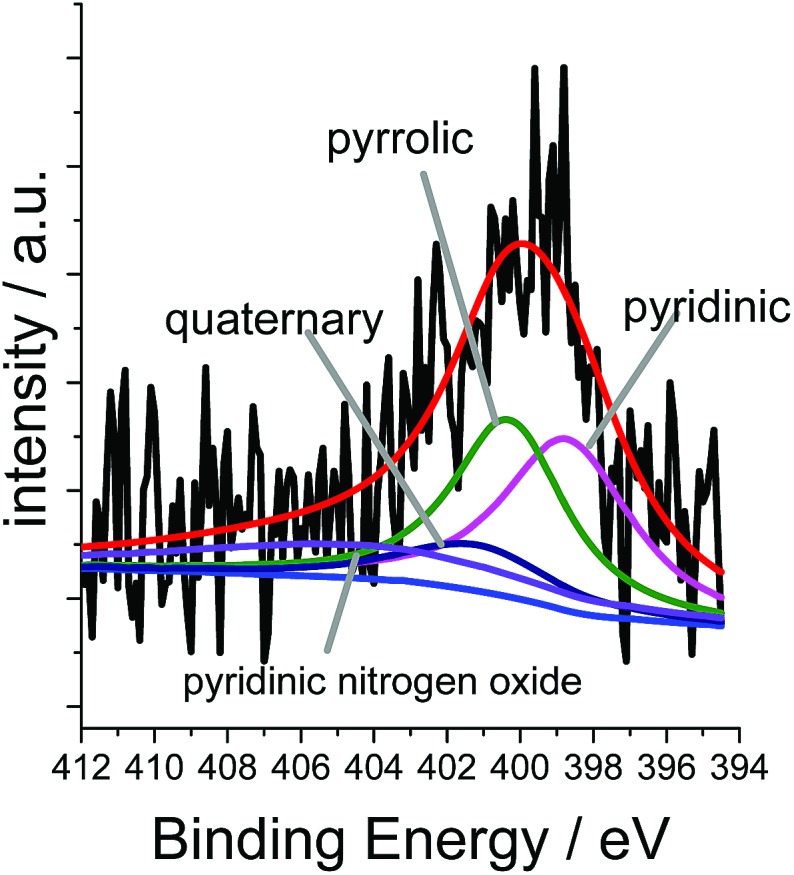
XPS measurement of Co/Melamine-2@C-700.

**Fig. 4:**
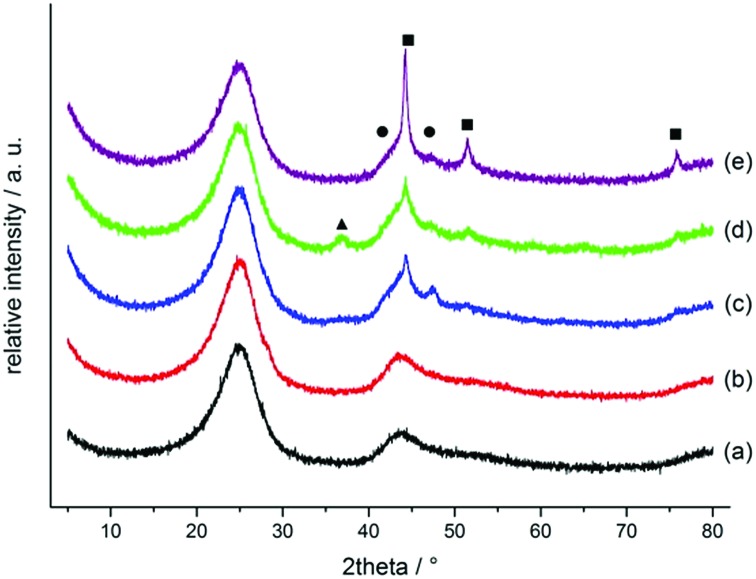
Powder diffraction pattern of supported Co/Melamine catalysts prepared at different conditions: pyrolysis at 400, 500, 600, 700 and 800 °C, respectively. Phases are labelled as squares (β-Co), circles (α-Co) and triangles (Co_3_O_4_).

XPS measurements for Co/Melamine-2@C-700 disclose that nitrogen is embedded into the carbon phase. More specifically, pyridinic, pyrrolic, quaternary nitrogen, as well as pyridinic nitrogen oxide were detected (Fig. 3). The total nitrogen content on the surface of this catalyst is 1.27 at%. In this case, EDX spectroscopy disclosed that dispersed cobalt is found in a carbon matrix (Fig. S5†). Subsequently, materials prepared using various amounts of melamine at different pyrolysis temperature were characterized by powder X-ray diffraction. No crystalline Co-phases can be identified by diffraction methods after pyrolysis at 400 and 500 °C. At higher pyrolysis temperature metallic cobalt is formed in both, hexagonal and cubic modifications. At 800 °C most of the α-Co is converted into its cubic form. Interestingly, some Co_3_O_4_ is detected at 600 and 700 °C which is not to be found at 800 °C (Fig. 4).

With optimal reaction conditions in hand, the hydrogenation of diverse N-heteroarenes was explored. As shown in Table 2, the substrate scope is broad and the functional group tolerance is excellent. Quinolines substituted with both electron-donating and electron-withdrawing groups are converted to the corresponding products in high to excellent yields. It is to note that substrates containing sensitive Br, OH, NH_2_, OTf substituents were smoothly reduced to give **2d**, **2i**, **2j**, **2o**, **2s**, and **2u**. Notably, the tolerance of Br and OTf substituents allow further transformations through standard coupling reactions. Furthermore, hydrogenation sensible COOMe, COOH, CN, C=O, C=C groups are compatible under this employed reaction conditions and the corresponding formamides (**2k**, **2m**, **2n**, **2t**, **2w**) were obtained in high yield and selectivity. Particularly, substrates featuring substituents, *e.g.* OH, Ac, NH_2_, CH_2_, Ph, on the pyridine ring also formed the desired products **2s**, **2t**, **2u**, **2v**, **2w**. Interestingly, quinolin-3-ol **1s** produced 3-oxo-3,4-dihydroquinoline-1(2*H*)-carbaldehyde **2s** in 64% yield through partial hydrogenation of pyridine ring followed by isomerization.

**Table 2:** Transfer hydrogenation of quinolines: substrate scope

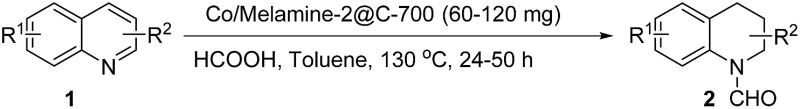					
Entry	Substrate	Product	Entry	Substrate	Product
1	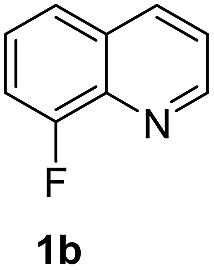	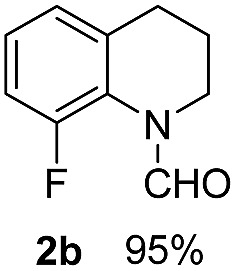	12	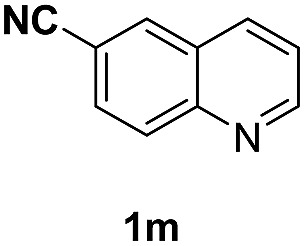	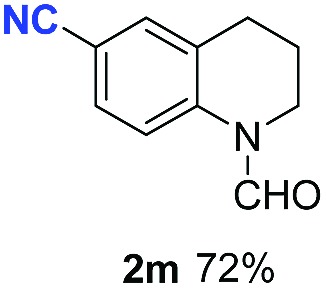
2	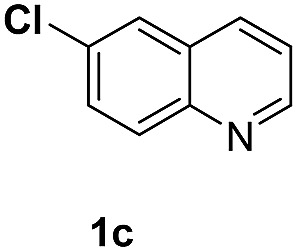	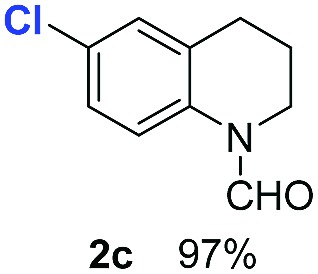	13	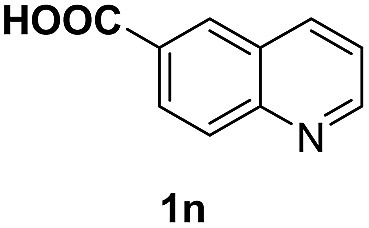	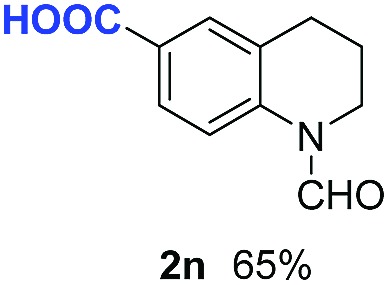
3	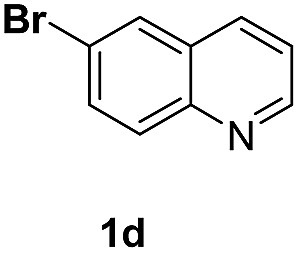	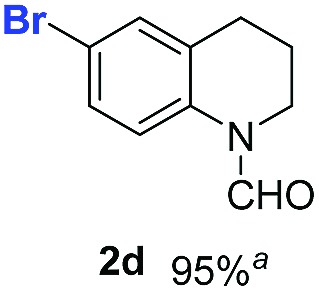	14	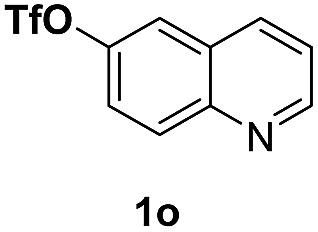	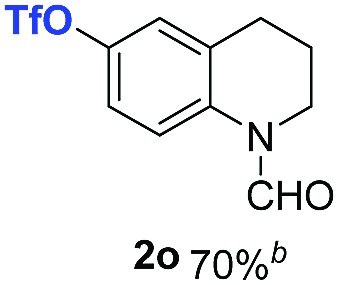
4	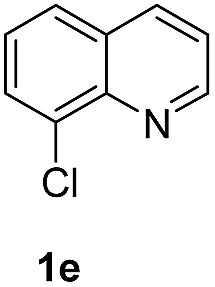	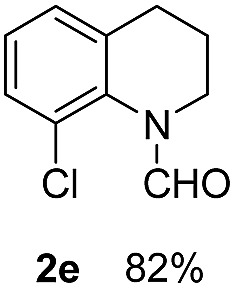	15	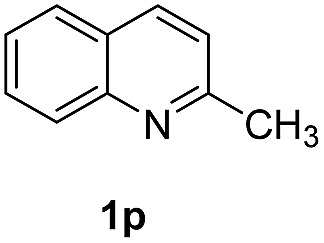	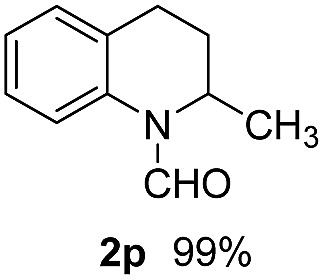
5	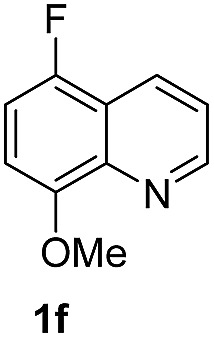	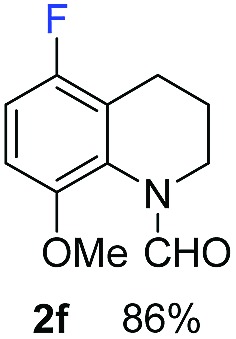	16	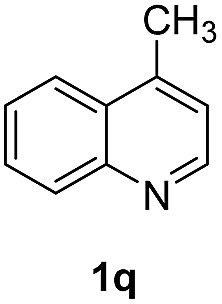	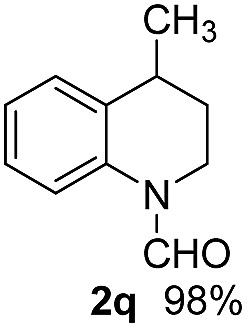
6	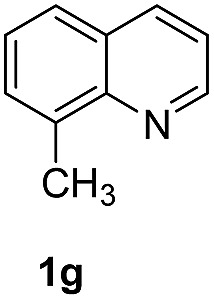	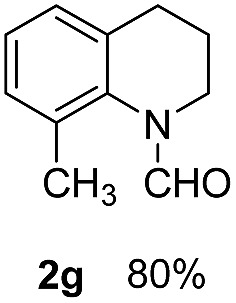	17	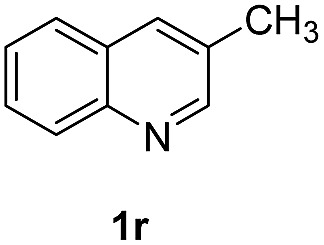	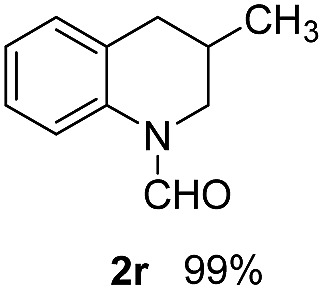
7	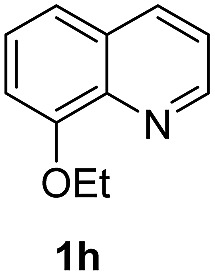	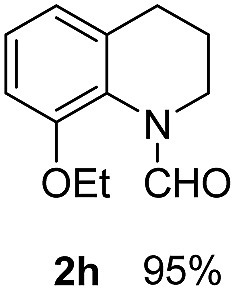	18	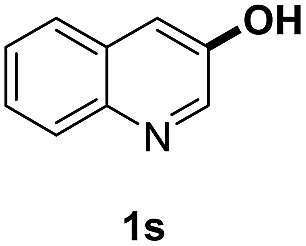	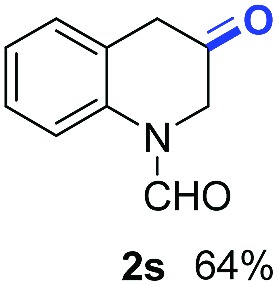
8	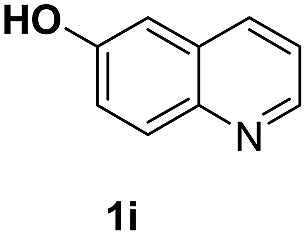	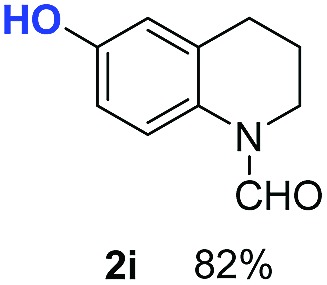	19	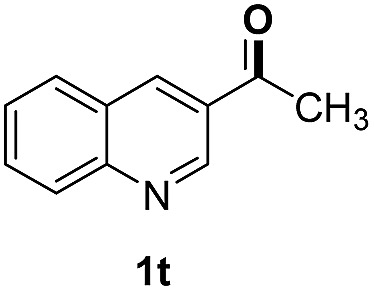	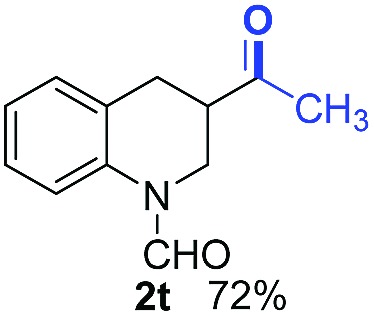
9	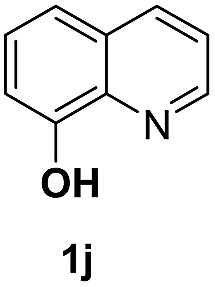	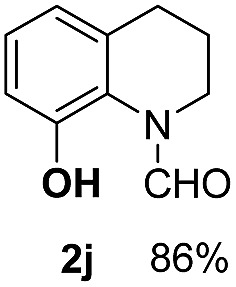	20	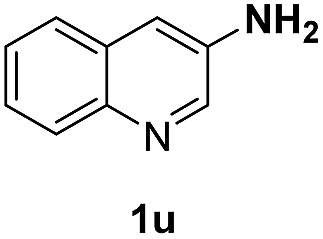	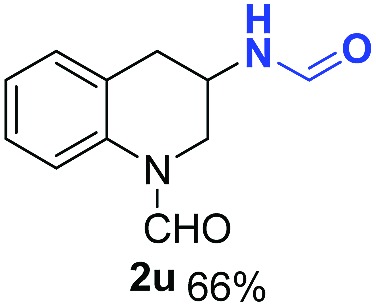
10	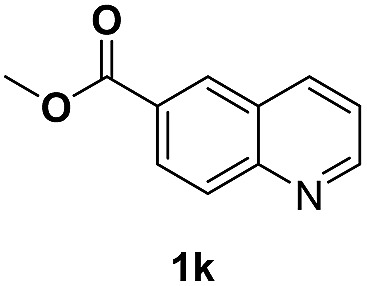	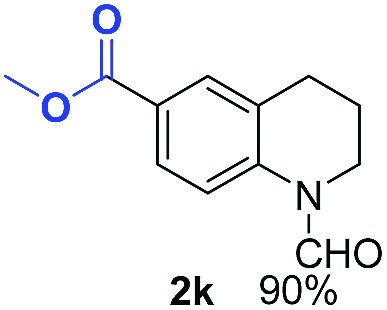	21	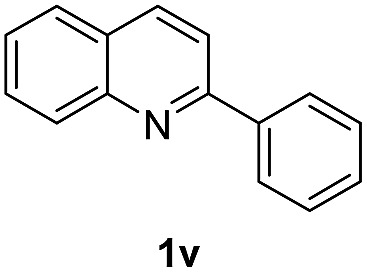	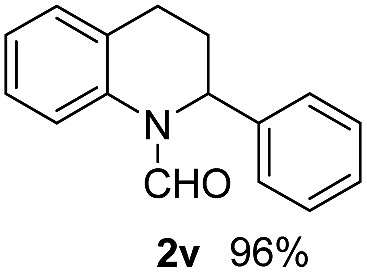
11	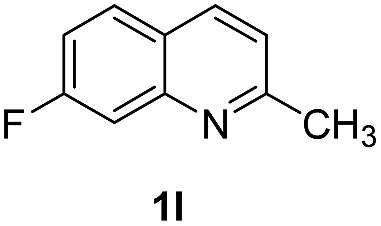	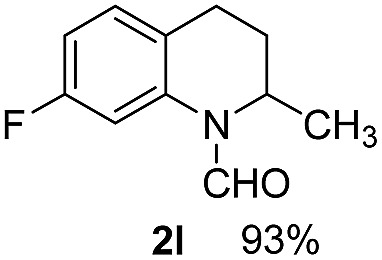	22	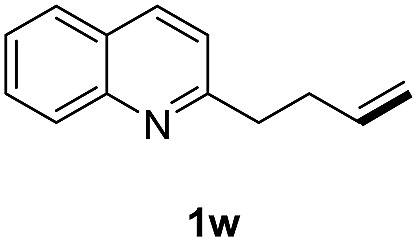	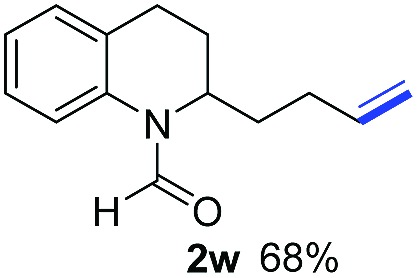

^*a*^1.0 equivalent of Et_3_N was used.

^*b*^3,4-Dihydroquinoline-1(2*H*)-carbaldehyde was obtained in 9% yield.

Apart from quinolines, other N-heteroarenes such as phenanthridine, phthalazine and 1,5-naphthyridine remained suitable substrates for this transformation delivering the corresponding formamides in moderate to high yield with good selectivity (Table 3). Furthermore, for phthalazine, only one C=N bond was hydrogenated keeping another C=C or C=N bond of pyridine ring intact (**9**).

**Table 3:** Transfer hydrogenation of other N-heteroarenes

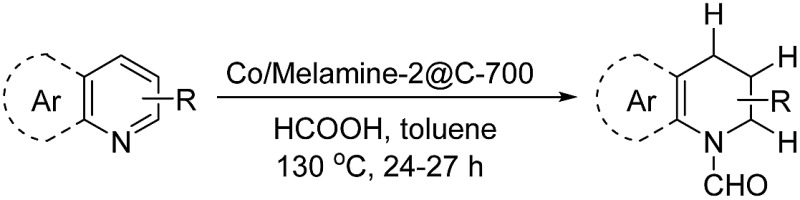					
Entry	Substrate	Catalyst (mol%)	HCOOH (equiv.)	Product	Yield (%)
1	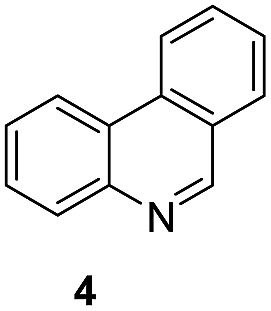	13.5	20	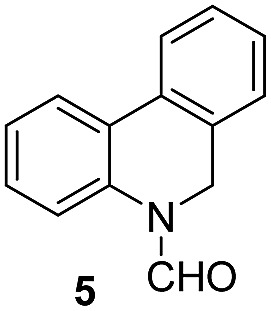	95
2	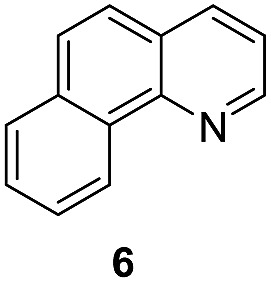	10	20	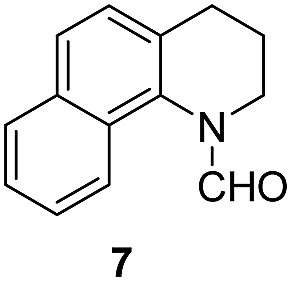	89
3	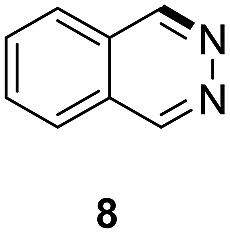	10	15	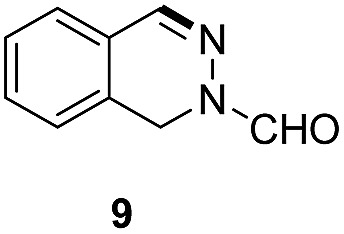	81
4	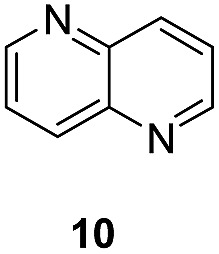	10	10	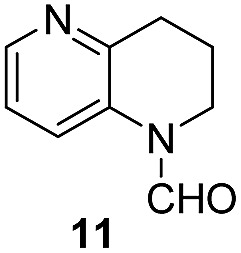	38^*a*^

^*a*^With 2.5 equivalent Et_3_N.

**Scheme 2:**
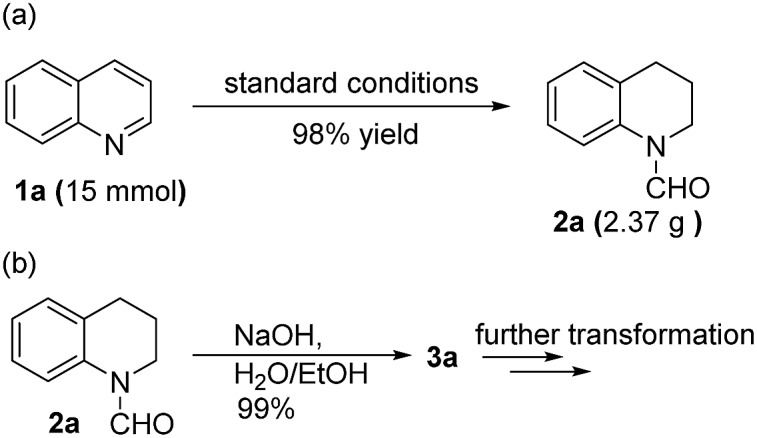
Up scaling experiment and further transformation of formamide.

**Fig. 5:**
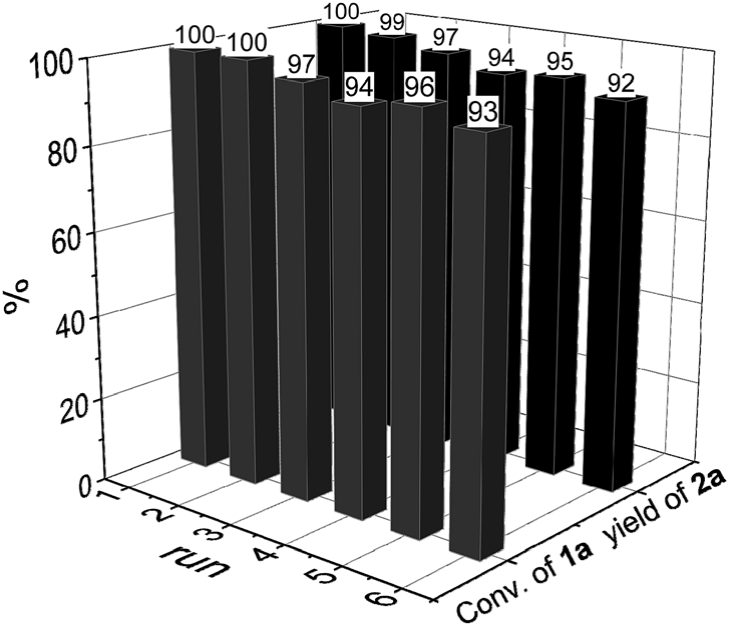
Recycling experiments.

**Fig. 6:**
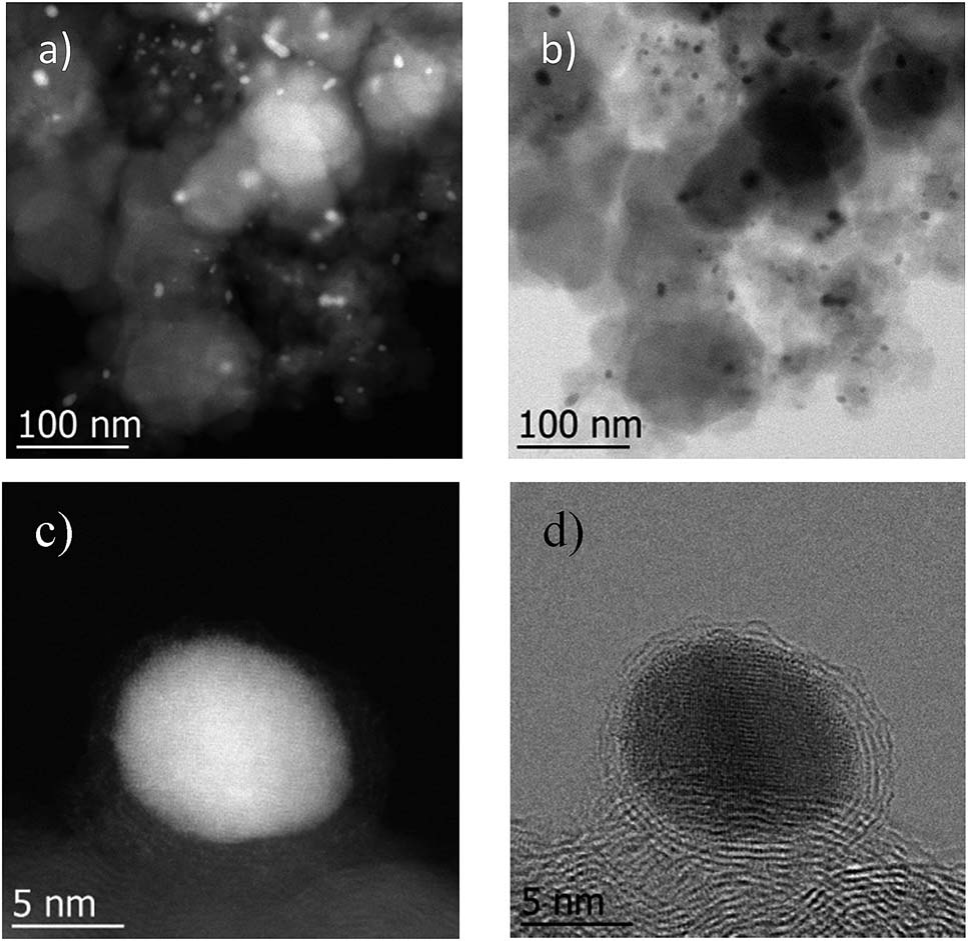
STEM images of Co/Melamine-2@C-700 after 4 runs. (a) high angle annular dark field (HAADF) overview of Co/Melamine-2@C-700 after 4 runs, (b) annular bright field (ABF) images of Co/Melamine-2@C-700 after 4 runs, (c and d) images showing metallic cobalt particles with graphitic layers were stable and remained after recycling experiments.

To demonstrate the practicability of our protocol, reaction of quinoline **1a** was performed on 15 mmol-scale to give **2a** in 98% yield (Scheme 2a). This product can be easily hydrolyzed to the amine, 1,2,3,4-tetrahydroquinoline (**3a**), in quantitative yield simply by using NaOH/H_2_O/EtOH (Scheme 2b). The stability and reusability are important features of heterogeneous catalysts. To explore the recycling of the active cobalt catalyst, experiments were carried out for the model substrate **1a** (Fig. 5). Notably, this heterogeneous cobalt catalyst showed no obvious deactivation after 6 runs and the selectivity remained excellent. Furthermore, the STEM images of Co/Melamine-2@C-700 after 4 runs were recorded and shown in Fig. 6. The metallic cobalt particles with graphitic layers were stable and remained after recycling experiments. However, the cobalt oxide phase aggregated and large cobalt oxide particle could be detected (see Fig. S6†).

**Fig. 7:**
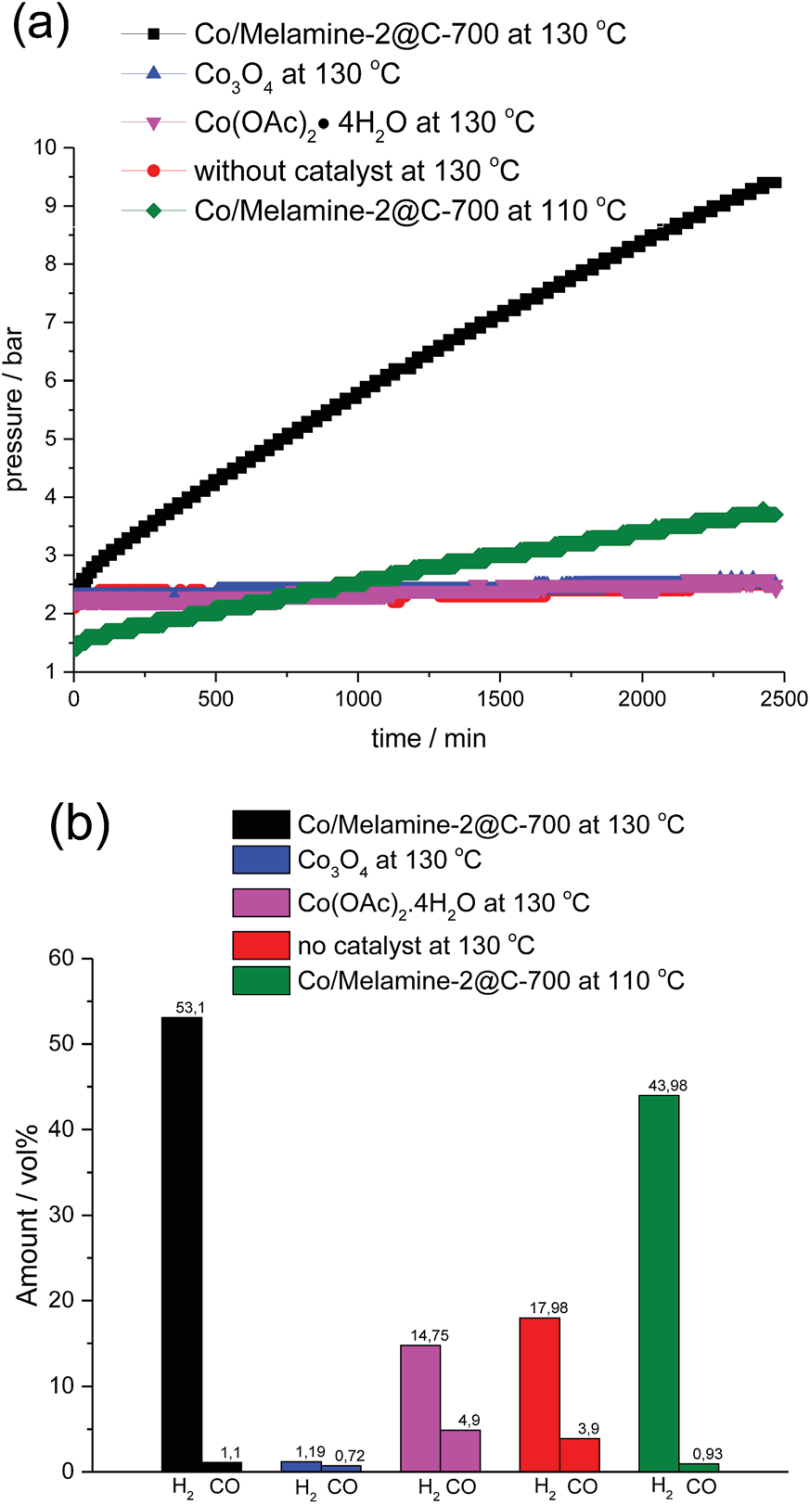
Hydrogen production from formic acid.

To investigate the mechanism of this heterogeneous transfer hydrogen process, several control experiments were conducted. In general, such reactions might proceed *via* initial formation of molecular hydrogen from formic acid and subsequent hydrogenation or alternatively straight from formic acid. In the latter case, the active metal hydride species will be directly generated from the corresponding Co formate species *via* β-hydride elimination. To proof the activity of our cobalt nanoparticles in the presence of H_2_, the reaction of quinoline was performed under 10 bar of hydrogen. Indeed, selective reduction took place and **3a** was obtained in 82% yield. Next, we studied hydrogen generation from formic acid in the presence of different cobalt catalysts (Fig. 7). All these reactions were conducted in a 100 mL autoclave, which was equipped with a pressure detector. As shown in Fig. 7a, commercially available Co_3_O_4_ as well as homogeneous Co(OAc)_2_·4H_2_O generated no or only very little amount of gas even after 40 h. On the other hand, the novel Co/Melamine-2@C-700 catalyst showed stable hydrogen formation throughout the reaction. Gas phase analysis by GC-TCD (see ESI†) revealed the highest hydrogen selectivity for the Co/Melamine-2@C-700 nanoparticles (Fig. 7b). Obviously, this novel material is also of significant interest for hydrogen generation processes in the context of a hydrogen economy, which is beyond the scope of this work.

**Scheme 3:**
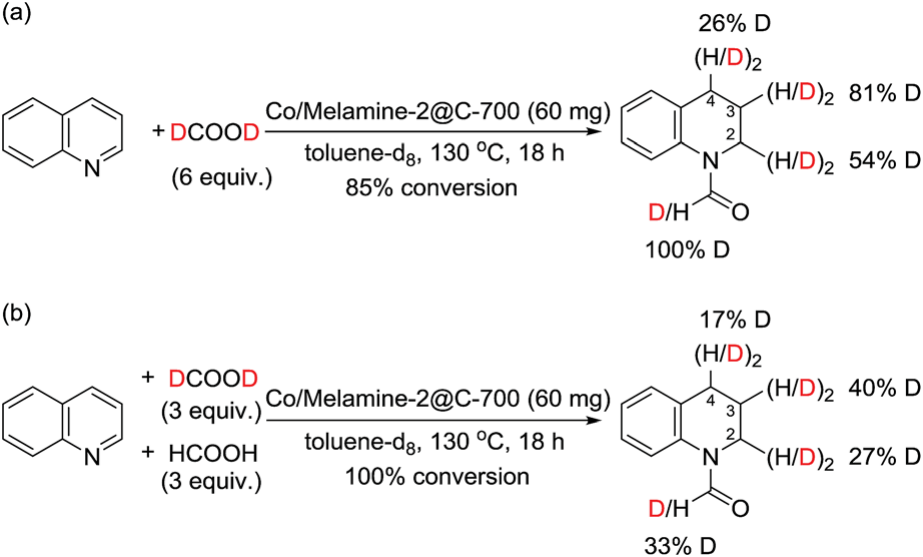
Transfer hydrogenation of quinoline: deuteration labelling experiments.

In addition, experiments with deuterated formic acid (DCOOD) instead of HCOOH were performed for our model reaction using 1,1,2,2-tetrachloroethane as internal standard. High conversion (85%) of quinoline **1a** was observed and the formyl group was deuterated in 100%, while the deuterium incorporation at the arene ring varied in between 26-81% (Scheme 3). This result shows that additional dehydrogenation/hydrogenation reactions catalyzed by this cobalt catalyst take place under the reaction conditions. From a competition experiment using DCOOD/HCOOH (1/1) it became evident that HCOOH is more active than DCOOD because of lower deuterium incorporation at all positions.

**Fig. 8:**
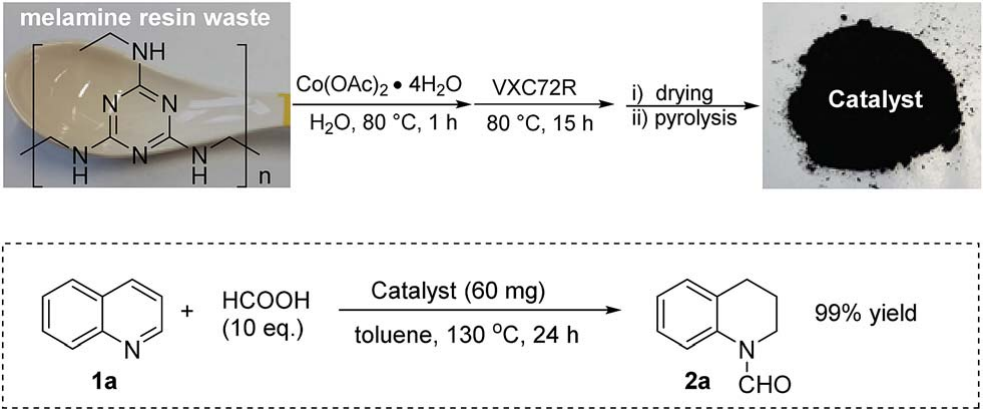
Catalyst preparation from melamine resin and its application.

Melamine is widely used for the production of consumer household and toy products *vide supra*. From these applications also significant amounts of waste are generated each year. For curiosity we became interested if the reuse of such waste materials to generate active catalysts is possible: hence, a commercial spoon made from melamine resin was finely crushed and the resulting powder was used to prepare the catalyst instead of pure melamine. ICP-MS analysis showed that no significant amounts of noble metals (<1 ppm) were present in the obtained powder. The obtained catalyst was tested for the benchmark hydrogenation and showed similar activity compared to the material obtained from pure melamine (Fig. 8).

## Conclusion

In conclusion, nitrogen modified heterogeneous cobalt catalysts supported on carbon were prepared in water using inexpensive melamine or melamine resins as nitrogen source. The optimal catalyst Co/Melamine-2@C-700 allows for selective transfer hydrogenation of diverse heteroarenes using formic acid as reductant. Compared to most known transfer hydrogenations, no addition of base is necessary to obtain sufficient catalyst activity. Compared to previously known heterogeneous catalysts, a broader substrate scope including quinolines, phenanthridine, phthalazine, and 1,5-naphthyridine as well as improved functional group tolerance were realized with the novel material. Interestingly, this non-noble metal catalyst shows also activity and selectivity for the dehydrogenation of formic acid, which will be explored further on in the area of energy technologies.
