# Supplementary material for: Selective cobalt nanoparticles for catalytic transfer hydrogenation of N-heteroarenes
Source: Chem Sci. 2017 Jul 12;8(9):6239–46. doi: 10.1039/c7sc02062g (PMC5628387; doi:10.1039/c7sc02062g)
Supplement: Supplementary file 1 [file SC-008-C7SC02062G-s001.pdf]

Electronic Supplementary Material (ESI) for Chemical Science.
This journal is © The Royal Society of Chemistry 2017

Selective Cobalt Nanoparticles for Catalytic Transfer Hydrogenation of N-Heteroarenes

Feng Chen,[a] Basudev Sahoo,[a] Carsten Kreyenschulte,[a] Henrik Lund,[a] Min Zeng,[b] Lin He,[b] Kathrin Junge,[a] and Matthias Beller*[a]

E-Mail: Matthias.Beller@catalysis.de

[a] Leibniz-Institut für Katalyse e.V. an der Universität Rostock, Albert-Einstein Straße 29a, Rostock, 18059, Germany.

[b] State Key Laboratory for Oxo Synthesis and Selective Oxidation, Suzhou Research Institute of LICP, Chinese Academy of Sciences, Lanzhou 730000, P. R. China.

Table of Contents:

1. General Remarks
2. Procedure for the Preparation of Catalysts
3. General Procedure for the Hydrogenation of Quinolines
4. Procedure for Catalyst Recycling
5. STEM Measurements of Catalyst
6. XPS Measurements of Catalyst
7. Hydrogen Production under Different Catalytic Systems
8. Deuterated Experiments
9. Characterization Data for the Substrate and Products
10. ^{1}H NMR, ^{13}C NMR and ^{19}F NMR Spectra of Substrates and Products
11. References

1. General Remarks

Unless otherwise stated, reactions were performed in autoclaves. Solvents were used directly without further purification. NMR spectra were recorded on Bruker AV 300 and 400 spectrometers. All chemical shifts (δ) are reported in parts per million (ppm) and coupling constants (J) in Hz. All chemical shifts are reported relative to tetramethylsilane (δ = 0.0 for ^{1}H NMR in $CDCl_3$) and *d*-solvent peaks (δ = 77.00 for ^{13}C NMR, chloroform), respectively. Mass spectra were recorded on an AMD 402/3 or a HP 5973 mass selective detector.

XRD powder pattern were recorded either on a Panalytical X'Pert diffractometer equipped with a Xcelerator detector or on a Panalytical Empyrean diffractometer equipped with a PIXcel 3D detector system, both used with automatic divergence slits and Cu Kα radiation (40 kV, 40 mA). Cu β-radiation was excluded by using nickel filter foil. If applied, Cu K α2 radiation was removed arithmetically using the Panalytical HighScore Plus software package. Peak positions and profile were fitted with Pseudo-Voigt function using the the Panalytical HighScore Plus software package Phase identification was done by using the PDF-2 database of the International Center of Diffraction Data (ICDD).

The TEM measurements were performed at 200kV with an aberration-corrected JEM-ARM200F (JEOL, Corrector: CEOS). The microscope is equipped with a JED-2300 (JEOL) energy-dispersive x-ray-spectrometer (EDXS) for chemical analysis.

The aberration corrected STEM imaging (High-Angle Annular Dark Field (HAADF) and Annular Bright Field (ABF)) were performed under the following conditions. HAADF and ABF both were done with a spot size of approximately 0.13nm, a convergence angle of 30-36° and collection semi-angles for HAADF and ABF of 90-170mrad and 11-22mrad respectively. Preparation of the TEM sample: The sample was deposed without any pretreatment on a holey carbon supported Cu-grid (mesh 300) and transferred to the microscope.

X-ray photoelectron spectra (XPS) were obtained using an ESCALAB250xi spectrometer equipped with a monochromatized Al Kα X-ray radiation source (hν = 1486.6 eV) with 20 eV pass energy. All binding energies were calibrated using the C1s peak at 284.8 eV as the reference. The XPS peaks were analyzed using a Shirley-type background and a nonlinear leastsquare fitting of the experimental data based on a mixed Gaussian/Lorentzian peak shape.

The metal precursor $Co(OAc)_2 \cdot 4H_2O$ was obtained from Sigma-Aldrich. Melamine was purchased from Alfa Aesar. VXC72R was obtained from Cabot. All reagents were used directly without further purification prior to use.

2. Procedure for the Preparation of Catalyst Co/Melamine-2@C-700

$Co(OAc)_2 \cdot 4H_2O$ (126.8 mg, 0.5 mmol) and melamine (127.0 mg, 1.0 mmol) (Co:melamine = 1:2 molar ratio) were stirred in water (30 mL) at 80 °C for 1hour. The support VXC72R (746.2 mg) was then added and the mixture was stirred at 80 °C for 15 h. Then the water was removed in vacuum and leaving behind a black solid that was dried at the pump. The sample was grinded to a fine powder which was then transferred to a ceramic crucible and placed in the oven. The latter was evacuated to ca. 5 mbar and then flushed with argon three times. The furnace was heated to 700 °C at a rate of 25 °C per minute and held at 700 °C for 2 hours under argon atmosphere. After the heating was switched off the oven was allowed to reach room temperature. During the whole process argon was constantly passed through the oven.

3. General Procedure for the Hydrogenation of Quinolines

The catalytic activity tests were performed in a 40 ml sealed tube. Quinolines **1** (0.5 mmol) formic acid (6-10 eq.) and cobalt catalyst (60 mg) were mixed with 1.5 mL toluene. The closed reaction tube was placed into the oil bath at 130 ^{o}C for 24 h. After completion of the reaction time, the sealed tube was cooled to room temperature. The crude reaction mixture was purified by flash chromatography on silicone gel (eluent: hexane / ethyl acetate) to afford corresponding products **2**.

4. Procedure for Catalyst Recycling

In a reaction tube (40 mL), quinoline **1a** (0.5 mmol) with Co/Melamine-2@C-700 (60 mg) was mixed with 1.5 mL toluene. The closed reaction tube was placed into the oil bath at 130 ^{o}C for 24 h. After completion of the reaction time, the sealed tube was cooled to room temperature. 50 μL dodecane was added into the solution and the yield was determined by GC analysis. The catalyst was isolated by centrifugation and reused for next reaction.

5. STEM Measurements of Catalyst

5.1 STEM Measurements of Co/Melamine-2@C-700

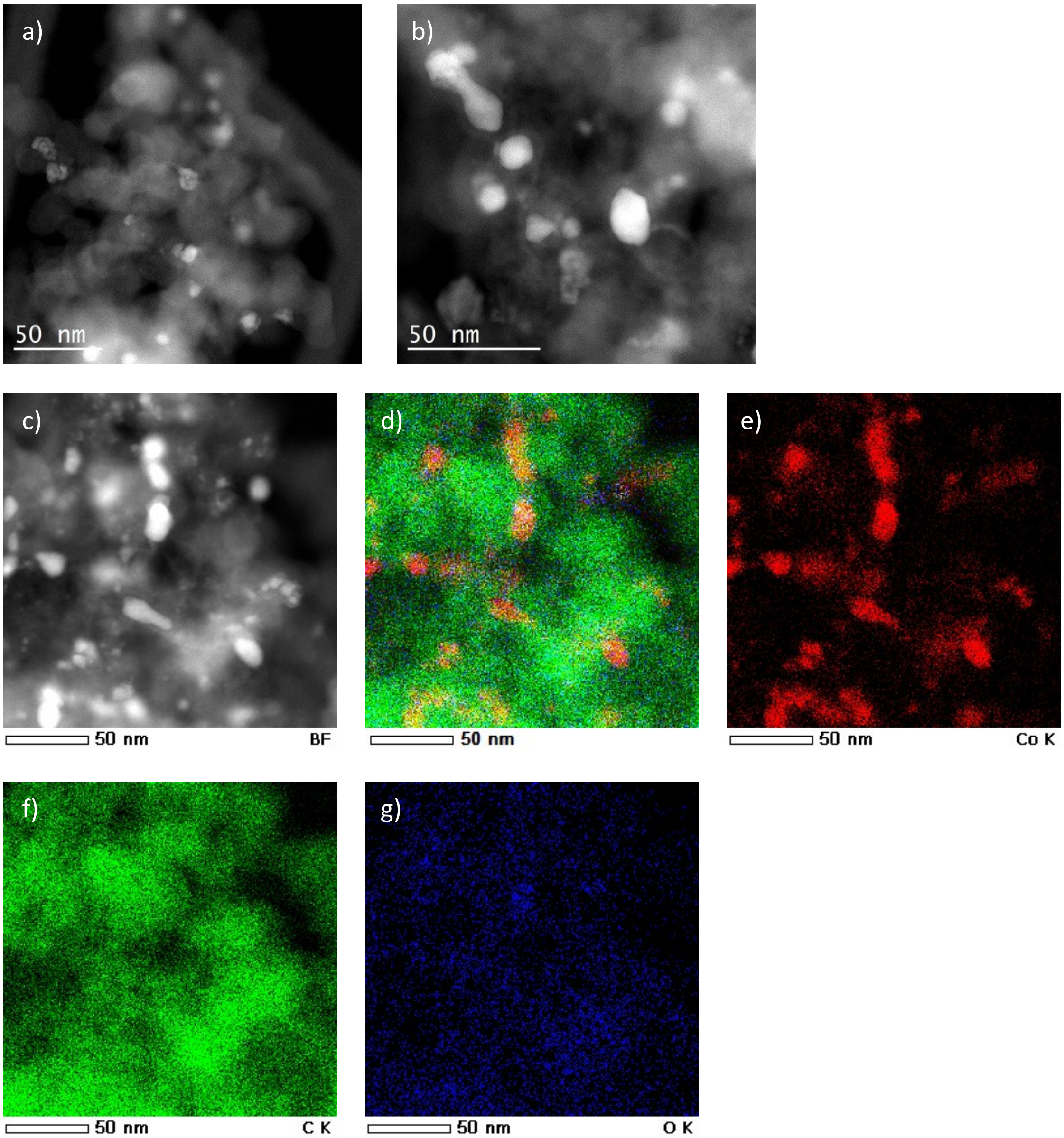

Figure S2. STEM measurements of Co/Melamine-2@C-700: a-c) HAADF images showing the variety of Co-containing particles such as metallic cobalt with graphene structure and cobalt oxide particles attached to the support, d-g) STEM-EDX mapping of the previous catalyst region showing the carbon/cobalt structure present in the fresh specimen: d) overlay of cobalt (red), carbon (green) and oxygen (blue) maps, e) cobalt map, f) carbon map, g) oxygen map.

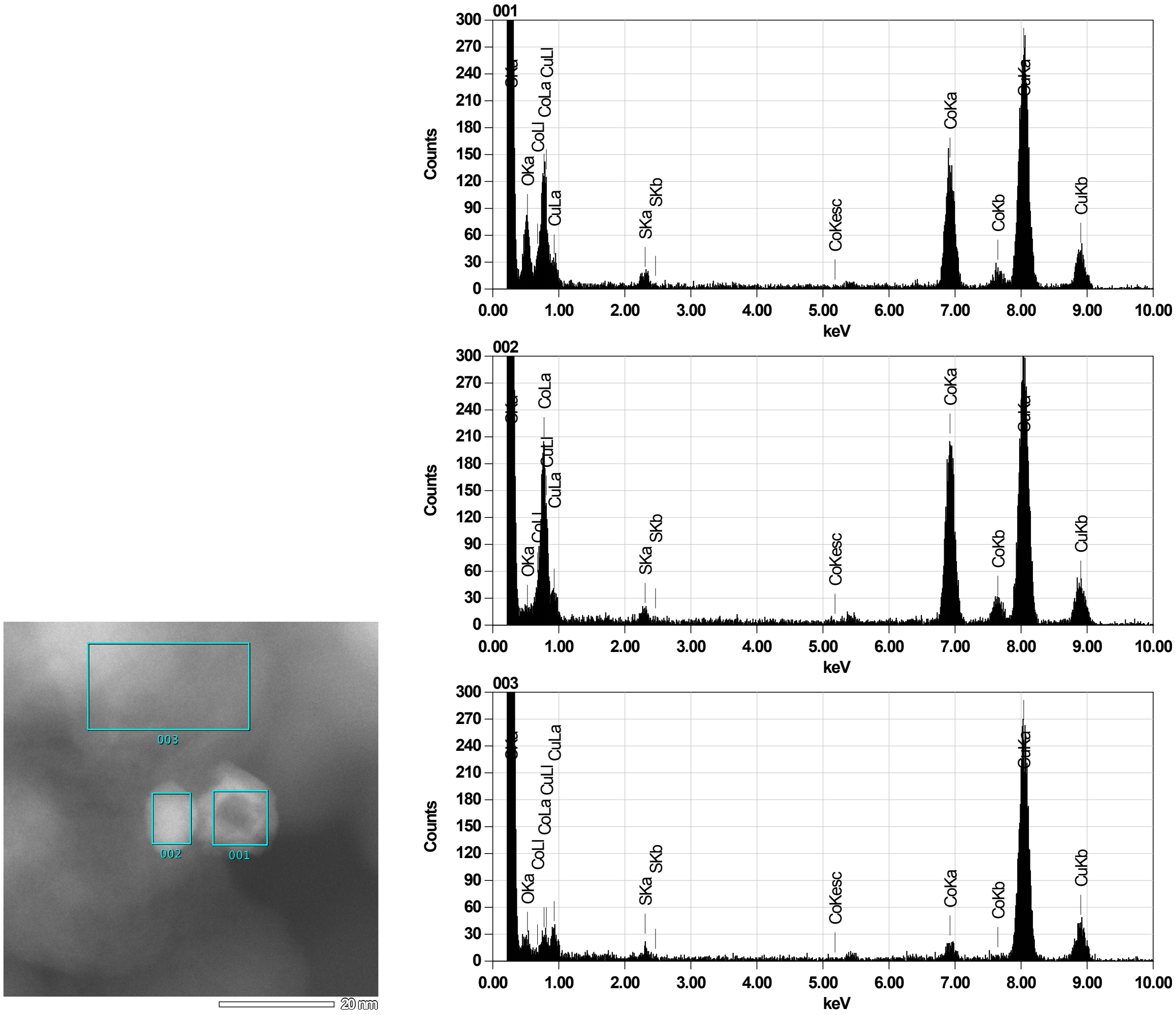

Figure S3. HAADF-STEM and EDX measurements of the indicated areas of Co/Melamine-2@C-700 show the spectral differences between the metallic and the oxidic Co particles (001 and 002) but also a remaining Co signal in an area of carbon support.

5.2 STEM Measurements of Co/Melamine-2@C-400

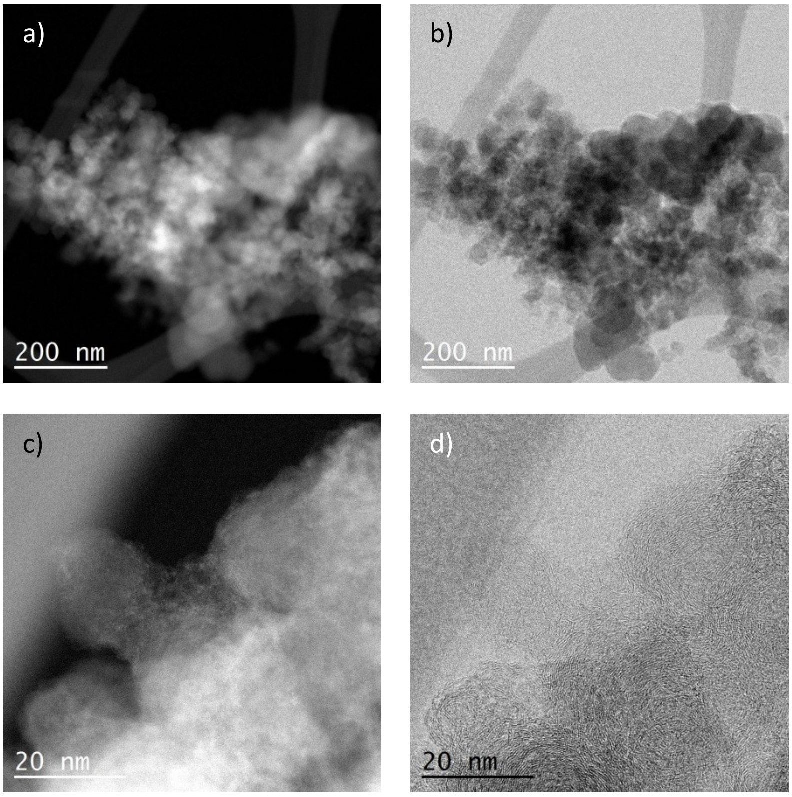

Figure S4. STEM measurements of Co/Melamine-2@C-400: a) high angle annular dark field (HAADF) overview of Co/Melamine-2@C-400, b) annular bright field (ABF) images of Co/Melamine-2@C-400, no cobalt particles were observed, c-d) images showing details of the carbon phase of Co/Melamine-2@C-400.

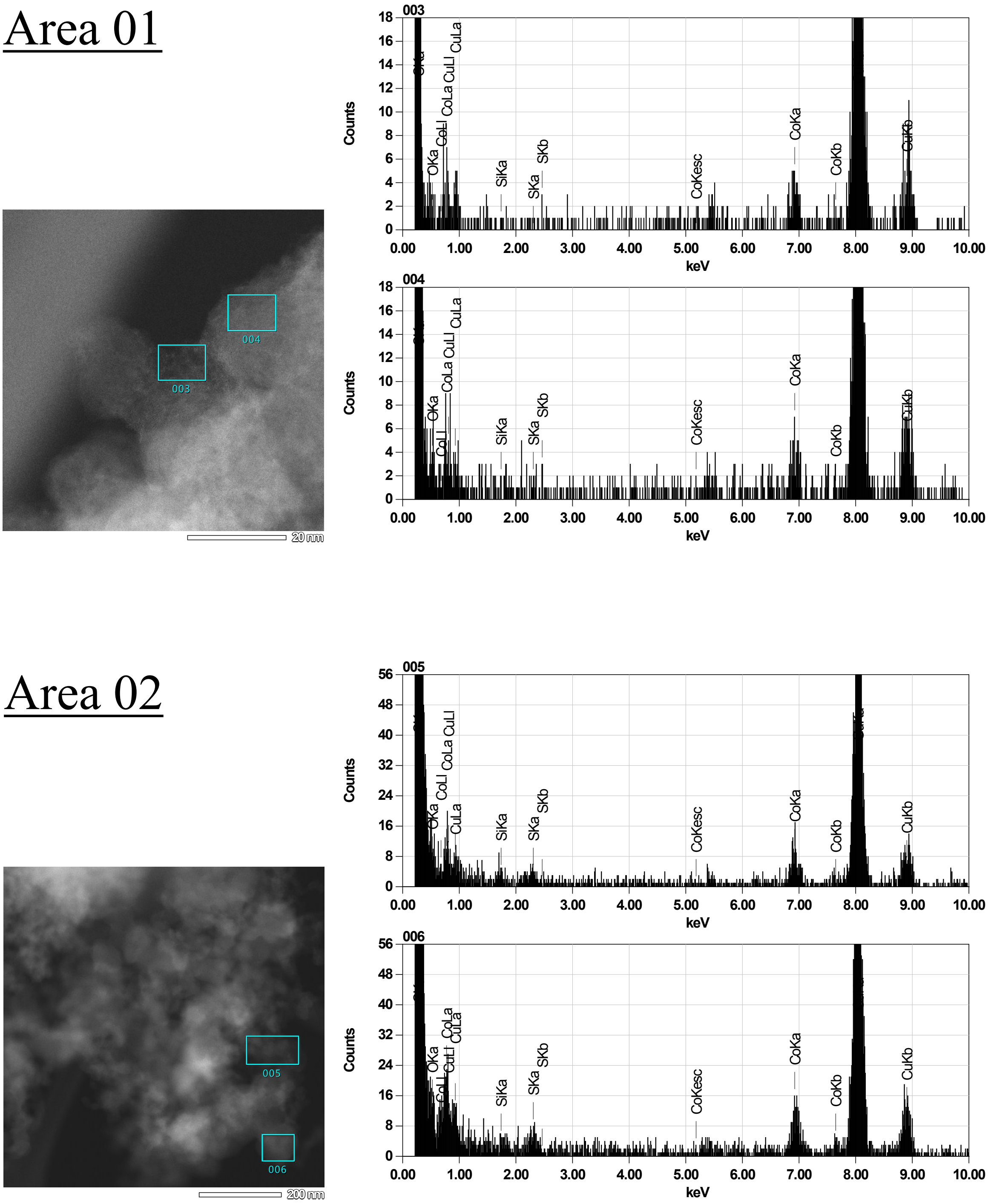

Figure S5. HAADF-STEM and EDX measurements of different areas of Co/Melamine-2@C-400 indicating the presence of finely distributed Co.

5.3 STEM Measurements of Co/Melamine-2@C-700 after 4 runs

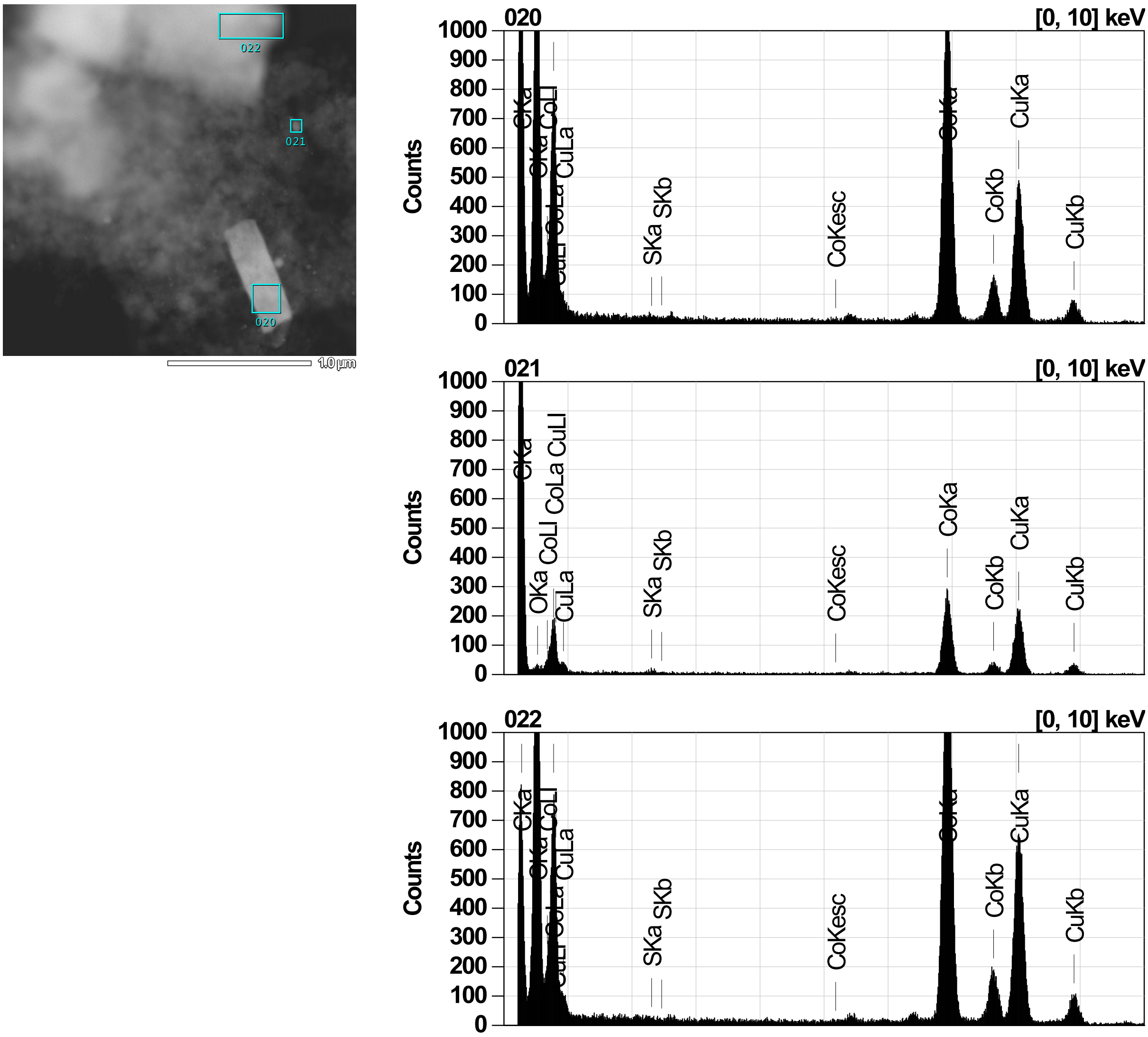

Figure S6. HAADF-STEM and EDX measurements of different areas of Co/Melamine-2@C-700after 4 runs indicating the presence of large cobalt oxide particle.

6. XPS Measurements of Catalyst

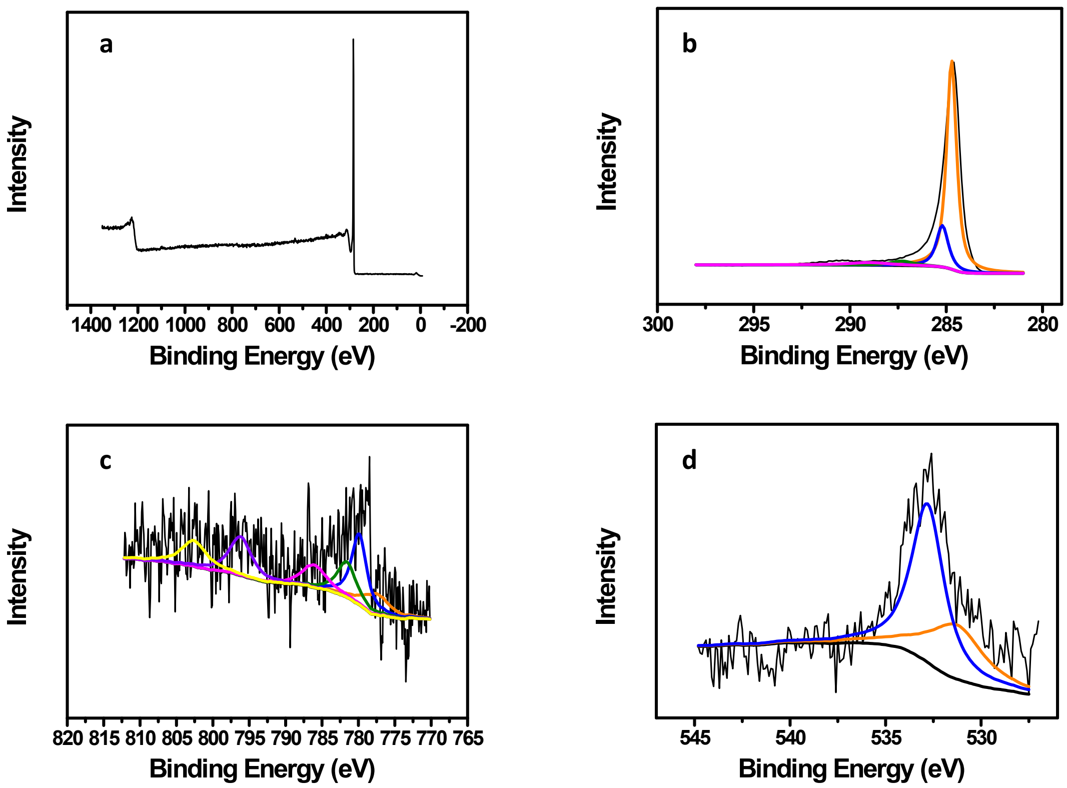

Figure S7. XPS Measurements of Co/Melamine-2@C-700. (a) survey (b) C1s (c) Co2p (d) O1s.

Table S1. XPS Measurements of different catalysts

entry	catalyst	Co	N	C	O
1	Co/Melamine-2/C-800	0.26	1.21	97.25	1.28
2	Co/Melamine-2/C-700	0.37	1.15	96.84	1.65
3	Co/Melamine-2/C-600	0.27	1.29	96.97	1.47
4	Co/Melamine-2/C-500	0.46	3.21	94.80	1.53
5	Co/Melamine-2/C-400	0.68	5.84	91.70	1.78
6	Co/Melamine-1/C-700	0.33	1.10	97.15	1.42
7	Co/Melamine-6/C-700	0.34	1.52	96.78	1.36

7. Hydrogen Production under Different Catalytic Systems

The reaction of 1.4 g HCOOH in 18.0 g toluene with or without catalyst was conducted in autoclave () for 40 h. The amount of gas which was formed after reaction was detected by GC after the autoclave cooled to room temperature.

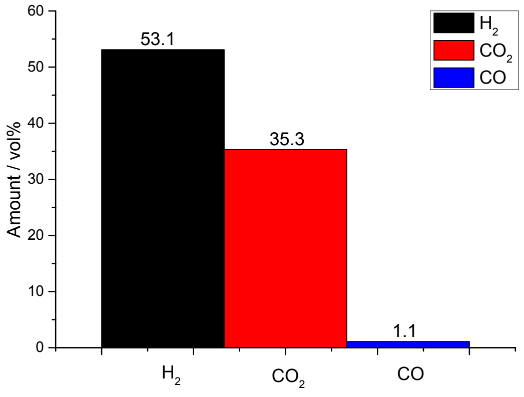

Figure S8. With Co/Mel(2)/C-700 at 130 °C (7.0 bar after cooling to room temperture)

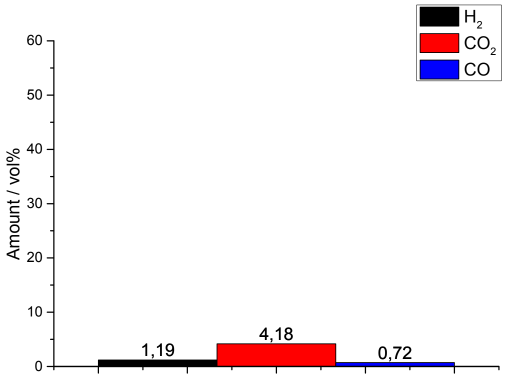

Figure S9. With Co_3O_4 at 130 °C (1.4 bar after cooling to room temperture)

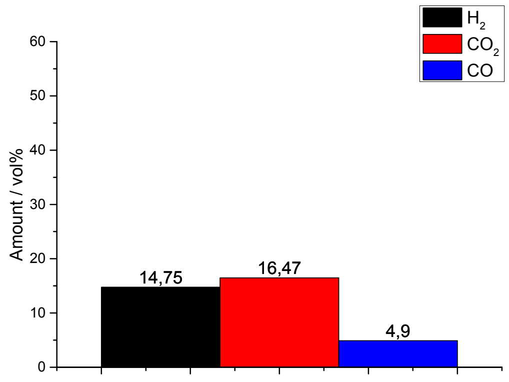

Figure S10. With Co(OAc)$_2$ at 130 ^{o}C (1.3 bar after cooling to room temperture)

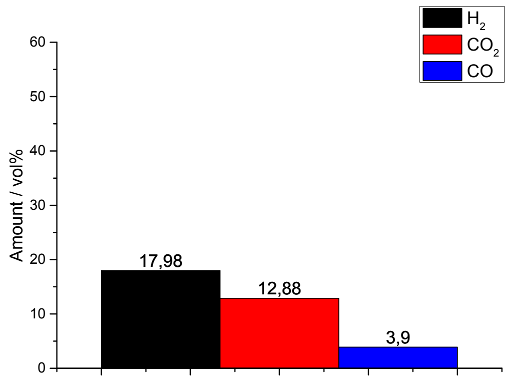

Figure S11. Without catalyst at 130 ^{o}C (1.3 bar after cooling to room temperture)

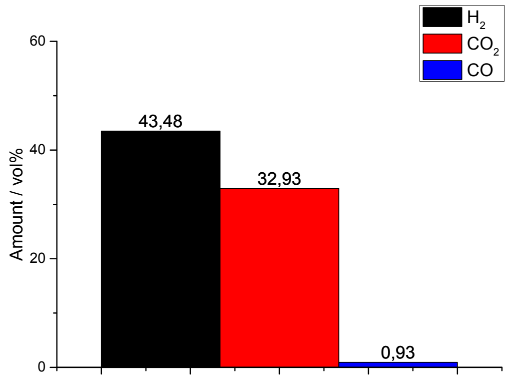

Figure S12. With Co/Mel(2)/C-700 at 110 ^{o}C (3.9 bar after cooling to room temperture)

8. Deuterated Experiments

The deuterated experiments were performed in a 40 ml sealed tube. Quinolines **1** (0.5 mmol), DCOOD (6 eq.) and cobalt catalyst (60 mg) were mixed with 1.5 mL toluene-d_8. The closed reaction tube was placed into the oil bath at 130 °C for 18 h. Then the sealed tube was cooled to room temperature. 1,1,2,2-tetrachloroethane (0.5 mmol) was added into the mixture and the crude reaction mixture was analyzed by NMR.

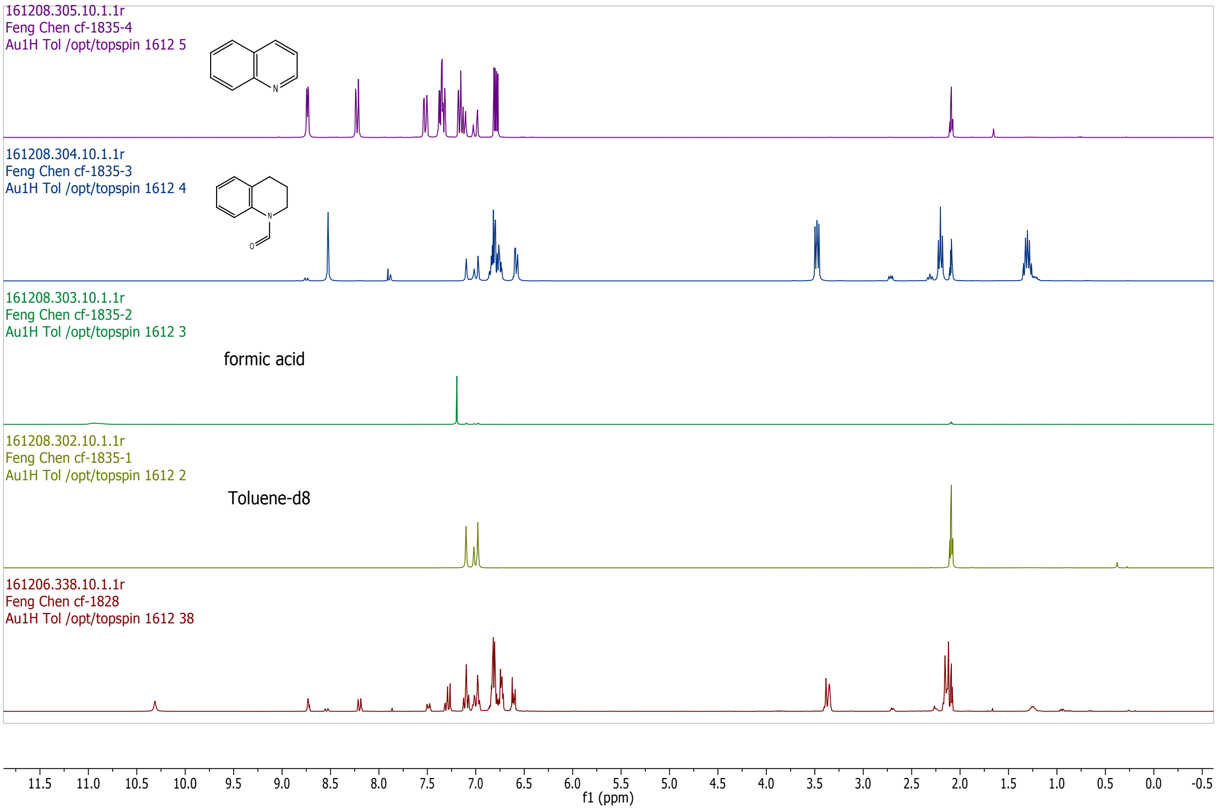

Figure S13. NMR spectrum in toluene-d_8

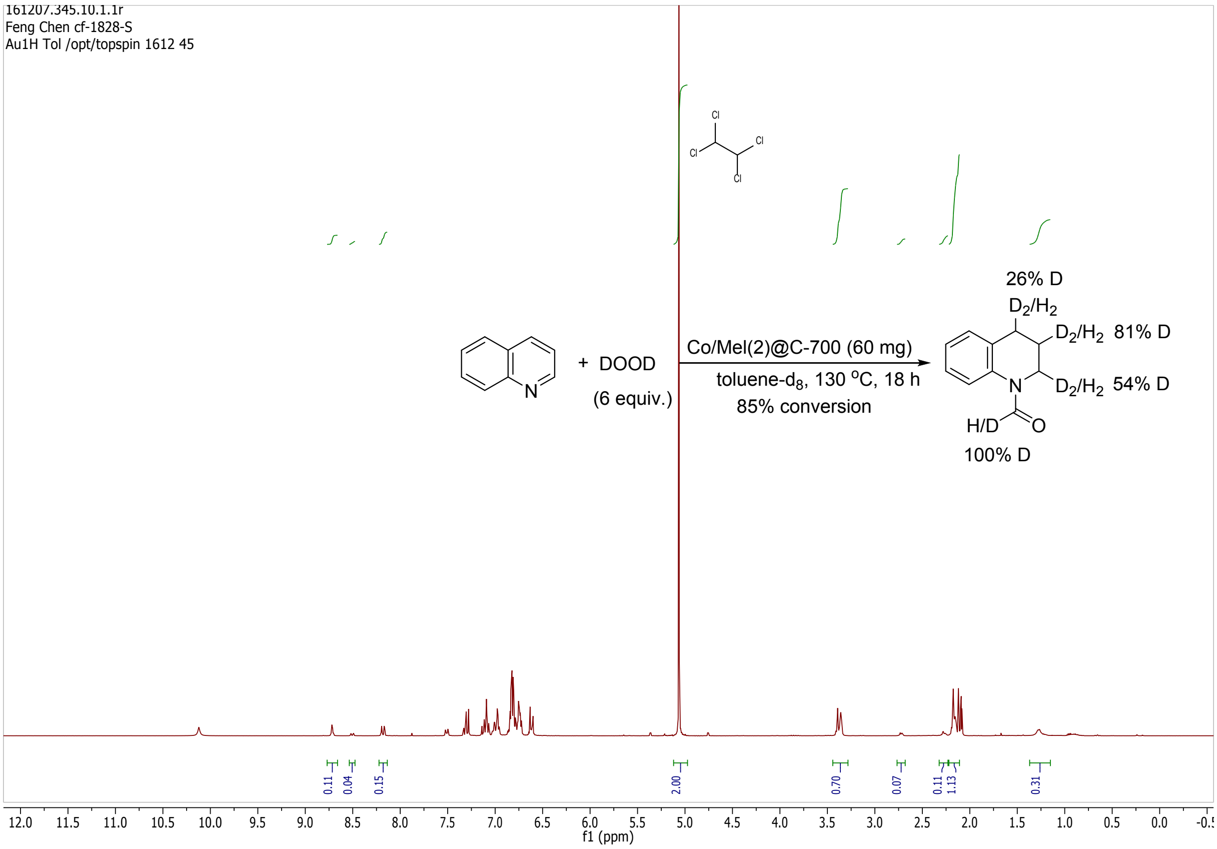

Figure S14. NMR spectrum of 1a with DCOOD in toluene-d_8 after reaction

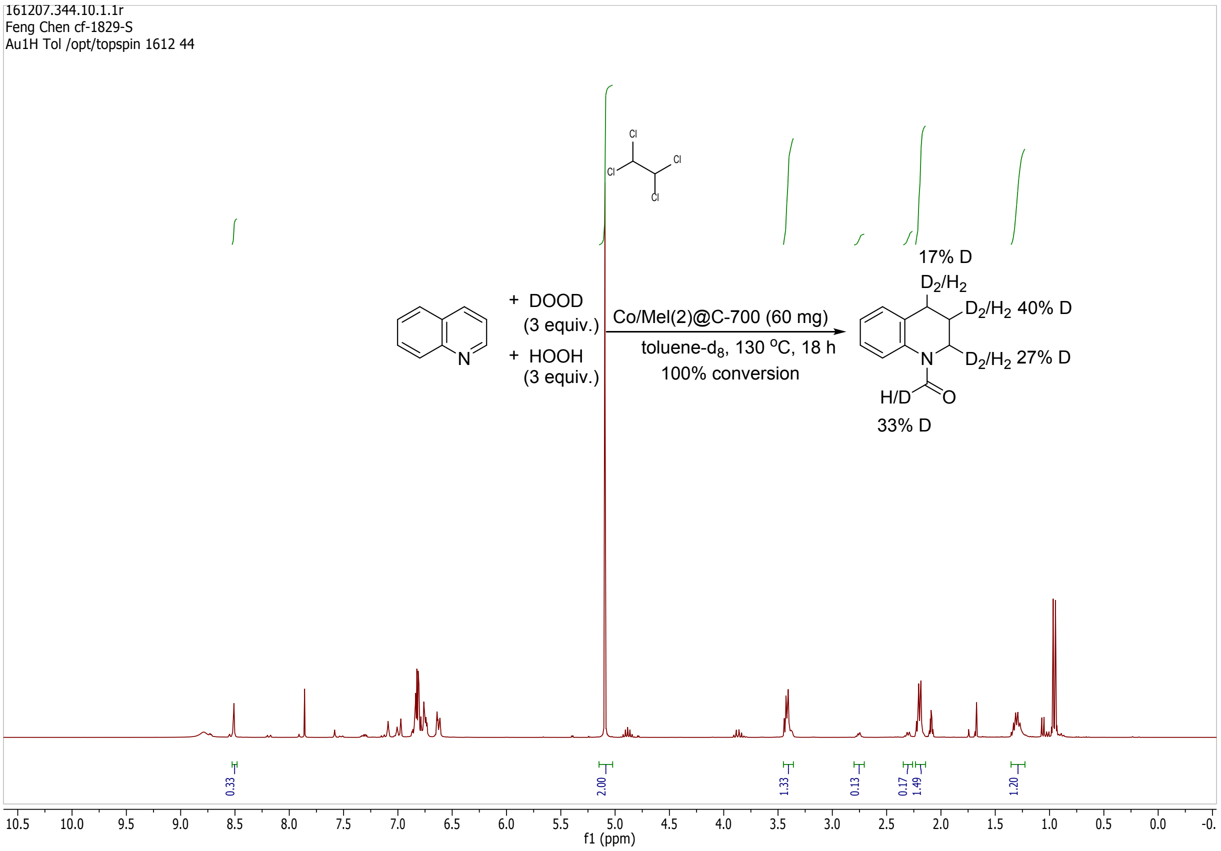

Figure S15. NMR spectrum of 1a with DCOOD/HCOOH mixture in toluene-d_8 after reaction

9. Characterization Data for the Substrate and Products

Synthesis of Substrate 1w:

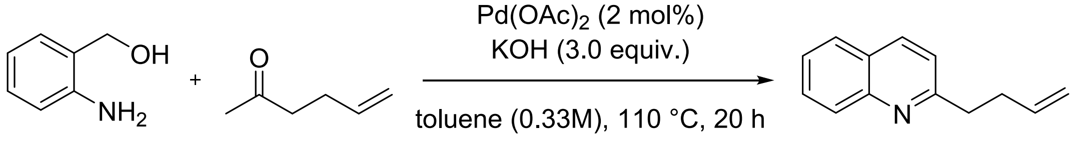

In a sealed Schlenk tube equipped with a magnetic stir bar, (2-aminophenyl)methanol (616 mg, 5.00 mmol), hex-5-en-2-one (1.16 mL, 10.0 mmol, 2.00 equiv.), palladium acetate (22.5 mg, 0.10 mmol, 0.02 equiv.) and potassium hydroxide (842 mg, 15.0 mmol, 3.00 equiv.) were dissolved in toluene (15 mL). The resulting reaction mixture was heated at 110°C for 20 h. After cooling to rt, the reaction mixture was concentrated under reduced pressure and purified by flash column chromatography over silica gel (eluent: hexane/ethylacetate = 9/1) to afford pure product **1w** as a pale yellow liquid (559 mg, 3.05 mmol, 61%).

^{1}H NMR (400 MHz, CDCl$_3$): δ (ppm): 8.06 (d, *J* = 8.3 Hz, 2H), 7.77 (dd, *J* = 8.0, 1.15 Hz, 1H), 7.68 (ddd, *J* = 8.5, 6.9, 1.5 Hz, 1H), 7.48 (ddd, *J* = 8.1, 6.9, 1.2 Hz, 1H), 7.29 (d, *J* = 8.4 Hz, 1H), 5.93 (ddt, *J* = 16.9, 10.1, 6.6 Hz, 1H), 5.06 - 5.11 (m, 1H), 4.98 - 5.01 (m, 1H), 3.06 – 3.10 (m, 2H), 2.56 – 2.63 (m, 2H); **^{13}C NMR (101 MHz, CDCl$_3$):** δ (ppm): 162.1, 148.0, 137.8, 136.4, 129.5, 128.9, 126.9, 125.9, 121.5, 115.4, 38.6, 33.9; **HR-MS (EI):** *m/z* calculated for $[C_{13}H_{13}N]^+$ ($[M]^+$): 183.1043, found: 183.1039.

8-fluoro-3,4-dihydroquinoline-1(2H)-carbaldehyde (2b)

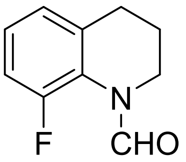

Reaction Conditions: 8-fluoroquinoline (75.8 mg, 0.50 mmol), HCOOH (10.0 equiv.), Co/Melamine-2@C-700 (60 mg), Toluene (1.5 mL), 130 °C, 24 h; **Isolated Yield:** 86.2 mg, 95% (purified as white solid by flash column chromatography over silica gel with hexane/ethyl acetate = 4/1); **^{1}H NMR (300 MHz, CDCl$_3$):** δ (ppm): 8.63 (d, *J* = 4.8 Hz, 1H), 6.84-6.99 (m, 3H), 3.74 (t, *J* = 6.3 Hz, 2H), 2.74 (t, *J* = 6.6 Hz, 2H), 1.89 (quintet, *J* = 6.3 Hz, 2H); **^{13}C NMR (75 MHz, CDCl$_3$):** δ (ppm): 163.2 (d, *J* = 12.8 Hz),154.2 (d, *J* = 243.8 Hz), 133.4, 124.9 (d, *J* = 8.3 Hz), 124.6 (d, *J* = 3.2 Hz), 114.6 (d, *J* = 21.6 Hz), 40.1, 27.1, 22.7; **HR-MS (ESI):** *m/z* calculated for $[C_{10}H_{10}FNONa]^+$ ($[M+Na]^+$): 202.0639, found: 202.0639.

6-chloro-3,4-dihydroquinoline-1(2H)-carbaldehyde (2c)

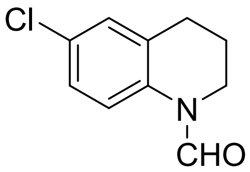

Reaction Conditions: 6-chloroquinoline (82.6 mg, 0.50 mmol), HCOOH (10.0 equiv.), Co/Melamine-2@C-700 (60 mg), Toluene (1.5 mL), 130 °C, 24 h; **Isolated Yield:** 95.2 mg, 97% (purified as colorless oil by flash column chromatography over silica gel with hexane/ethyl acetate = 4/1); **^{1}H NMR (300 MHz, CDCl$_3$) (major conformer):** δ (ppm): 8.67 (s, 1H), 6.98-7.11 (m, 3H), 3.72 (t, *J* = 6.0 Hz, 2H), 2.72 (t, *J* = 6.3 Hz, 2H), 1.87 (quintet, *J* = 6.3 Hz, 2H); **^{13}C NMR (75 MHz, CDCl$_3$) (major conformer):** δ (ppm): 160.7, 135.8, 130.5, 129.8, 129.4, 127.1, 118.1, 40.2, 27.0, 21.9; **HR-MS (ESI):** *m/z* calculated for $[C_{10}H_{10}ClNONa]^+$ ($[M+Na]^+$): 218.0343, found: 218.0346.

6-bromo-3,4-dihydroquinoline-1(2H)-carbaldehyde (2d)

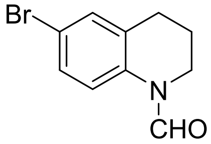

Reaction Conditions: 6-bromoquinoline (107.2 mg, 0.5 mmol), TEA (1 equiv.), HCOOH (15.0 equiv.), Co/Melamine-2@C-700 (60 mg), Toluene (1.5 mL), 130 °C, 48 h; **Isolated Yield:** 115.0 mg, 95% (purified as colorless solid by flash column chromatography over silica gel with hexane/ethyl acetate = 3/1); **^{1}H NMR (300 MHz, CDCl$_3$) (major conformer):** δ (ppm): 8.74 (s, 1H), 7.27-7.31 (m, 2H), 6.98-7.05 (m, 1H), 3.75-3.80 (m, 2H), 2.78 (t, *J* = 6.3 Hz, 2H), 1.93 (quintet, *J* = 6.3 Hz, 2H); **^{13}C NMR (75 MHz, CDCl$_3$) (major conformer):** δ (ppm): 160.7, 136.3, 132.3, 130.9, 130.0, 118.4, 117.4, 40.2, 26.9, 21.9; **HR-MS (EI):** *m/z* calculated for $[C_{10}H_{10}NOBr]^+$ ($[M]^+$): 238.99403, found: 238.99485.

8-Chloro-3,4-dihydroquinoline-1(2H)-carbaldehyde (2e)

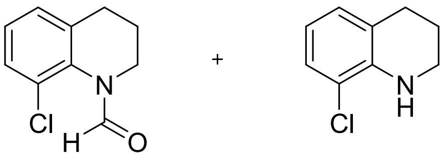

Reaction Conditions: 8-chloroquinoline (81.8 mg, 0.50 mmol), HCOOH (20.0 equiv.), Co/Melamine-2@C-700 (80 mg), Toluene (1.5 mL), 130 °C, 40 h; **Isolated Yield:** 8-chloro-3,4-

dihydroquinoline-1(2*H*)-carbaldehyde (colorless oil, 80 mg, 0.41 mmol, 82%) and 8-chloro-1,2,3,4-tetrahydroquinoline (colorless oil, 11 mg, 0.07 mmol, 13%) were obtained (purified by flash column chromatography over silica gel with heptane/ethyl acetate = 4/1)

8-Chloro-3,4-dihydroquinoline-1(2*H*)-carbaldehyde: ^{1}H NMR (300 MHz, $CDCl_3$) (major conformer): δ (ppm): 8.61 (s, 1H), 7.25 - 7.30 (m, 1H), 7.05 - 7.09 (m, 2H), 3.76 (t, *J* = 6.7 Hz, 2H), 2.70 (t, *J* = 6.6 Hz, 2H), 1.94 (quin., *J* = 6.6 Hz, 2H); **^{13}C NMR (75 MHz, $CDCl_3$) (major conformer):** δ (ppm): 163.5, 136.0, 135.0, 128.8, 127.2, 126.5, 126.3, 40.0, 27.1, 23.4); **HR-MS (ESI):** *m/z* calculated for $[C_{10}H_{10}ClNONa]^+$ ($[M+Na]^+$): 218.0343, found: 218.0342.

8-Chloro-1,2,3,4-tetrahydroquinoline: ^{1}H NMR (300 MHz, $CDCl_3$): δ (ppm): 7.02 - 7.12 (m, 1H), 6.86 (dq, *J* = 7.5, 1.1 Hz, 1H), 6.52 (t, *J* = 7.7 Hz, 1H), 4.47 (s, 1H), 3.40 (t, *J* = 5.3 Hz, 2H), 2.78 (t, *J* = 6.4 Hz, 2H), 1.90 - 1.98 (m, 2H); **^{13}C NMR (75 MHz, $CDCl_3$):** δ (ppm): 140.7, 127.8, 126.9, 122.8, 118.2, 116.5, 41.9, 27.3, 21.7); **HR-MS (ESI):** *m/z* calculated for $[C_9H_{11}ClN]^+$ ($[M+H]^+$): 168.0575, found: 168.0574.

5-fluoro-8-methoxy-3,4-dihydroquinoline-1(2H)-carbaldehyde (2f)

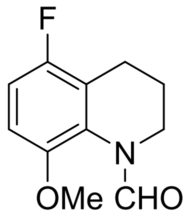

Reaction Conditions: 5-fluoro-8-methoxyquinoline (90.3 mg, 0.50 mmol), HCOOH (15.0 equiv.), Co/Melamine-2@C-700 (60 mg), Toluene (1.5 mL), 130 °C, 24 h; **Isolated Yield:** 90.2 mg, 86% (purified as white solid by flash column chromatography over silica gel with hexane/ethyl acetate = 3/1); **^{1}H NMR (300 MHz, $CDCl_3$):** δ (ppm): 8.74 (s, 1H), 6.73-6.83 (m, 3H), 3.78 (s, 3H), 3.73-3.78 (m, 2H), 2.75 (td, *J* = 6.6, 1.2 Hz, 2H), 1.91 (quintet, *J* = 6.6 Hz, 2H); **^{13}C NMR (75 MHz, $CDCl_3$):** δ (ppm): 164.0, 154.8 (d, *J* = 236.6 Hz), 147.4, 128.0 (d, *J* = 7.2 Hz), 119.6 (d, *J* = 21.5 Hz), 110.6, 110.2 (d, *J* = 9.5 Hz), 56.3, 39.3, 22.0, 20.2 (d, *J* = 3.5 Hz); **^{19}F NMR (282.4 MHz, $CDCl_3$):** δ (ppm): -126.02; **HR-MS (ESI):** *m/z* calculated for $[C_{11}H_{12}FNO_2Na]^+$ ($[M+Na]^+$): 232.0744, found: 232.0745.

8-methyl-3,4-dihydroquinoline-1(2H)-carbaldehyde (2g)

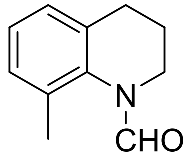

Reaction Conditions: 8-methylquinoline (72.2 mg, 0.50 mmol), HCOOH (10.0 equiv.), Co/Melamine-2@C-700 (60 mg), Toluene (1.5 mL), 130 °C, 24 h; **Isolated Yield:** 70.0 mg, 80% (purified as colorless oil by flash column chromatography over silica gel with hexane/ethyl acetate = 4/1); **^{1}H NMR (300 MHz, CDCl$_3$):** δ (ppm): 8.40 (s, 1H), 7.05-7.20 (m, 3H), 3.85 (t, *J* = 6.9 Hz, 2H), 2.74 (t, *J* = 6.6 Hz, 1H), 2.39 (s, 3H), 2.00 (quintet, *J* = 6.6 Hz, 2H); **^{13}C NMR (75 MHz, CDCl$_3$):** δ (ppm): 163.5, 136.7, 134.5, 130.0, 129.8, 126.1, 125.6, 40.1, 27.0, 23.5, 18.7; **HR-MS (ESI):** *m/z* calculated for $[C_{11}H_{13}NONa]^+$ ($[M+Na]^+$): 198.0889, found: 198.0891.

8-ethoxy-3,4-dihydroquinoline-1(2H)-carbaldehyde (2h)

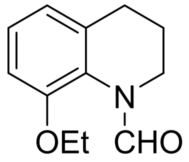

Reaction Conditions: 8-ethoxyquinoline (87.4 mg, 0.50 mmol), HCOOH (10.0 equiv.), Co/Melamine-2@C-700 (60 mg), Toluene (1.5 mL), 130 °C, 24 h; **Isolated Yield:** 98.0 mg, 95% (purified as colorless oil by flash column chromatography over silica gel with hexane/ethyl acetate = 3/1); **^{1}H NMR (300 MHz, CDCl$_3$):** δ (ppm): 8.81 (s, 1H), 7.09 (t, *J* = 7.8 Hz, 1H), 6.76-6.93 (m, 2H), 4.08 (q, *J* = 6.9 Hz, 2H), 3.83 (t, *J* = 6.3 Hz, 2H), 2.78 (t, *J* = 6.3 Hz, 2H), 1.98 (quintet, *J* = 6.3 Hz, 2H), 1.45(t, *J* = 6.9 Hz, 2H); **^{13}C NMR (75 MHz, CDCl$_3$):** δ (ppm): 164.4, 150.7, 133.4, 126.8, 125.2, 121.0, 111.1, 64.5, 40.0, 27.1, 23.2, 14.7; **HR-MS (ESI):** *m/z* calculated for $[C_{12}H_{15}NO_2Na]^+$ ($[M+Na]^+$): 228.0995, found: 228.0996.

6-Hydroxy-3,4-dihydroquinoline-1(2*H*)-carbaldehyde (2i)

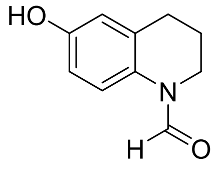

Reaction Conditions: quinolin-6-ol (72.6 mg, 0.50 mmol), HCOOH (10.0 equiv.), Co/Melamine-2@C-700 (60 mg), Toluene (1.5 mL), 130 °C, 24 h; **Isolated Yield:** 73 mg, 0.41 mmol, 82% (purified as white solid by flash column chromatography over silica gel with heptane/ethyl acetate = 2/1 to 1/1); **^{1}H NMR (300 MHz, DMSO-d$_6$) (major conformer):** δ (ppm): 9.23 (s, 1H), 8.65 (s, 1H), 7.13 - 7.16 (m, 1H), 6.56 - 6.59 (m, 2H), 3.62 (t, *J* = 6.1 Hz, 2H), 2.68 (t, *J* = 6.4 Hz, 2H), 1.79 (quin, *J* = 6.3 Hz, 2H); **^{13}C NMR (75 MHz, DMSO-d$_6$) (major conformer):** δ (ppm): 160.7, 154.0, 129.7, 129.0, 118.4, 115.5, 113.7, 40.1, 26.6, 22.0 (N.B.: δ (ppm) = 40.1 was detected by DEPT analysis); **HR-MS (ESI):** *m/z* calculated for $[C_{10}H_{11}NO_2Na]^+$ ($[M+Na]^+$): 200.0682, found: 200.0680.

8-Hydroxy-3,4-dihydroquinoline-1(2H)-carbaldehyde (2j)

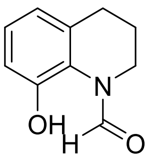

Reaction Conditions: quinolin-8-ol (72.6 mg, 0.50 mmol), HCOOH (15.0 equiv.), Co/Melamine-2@C-700 (60 mg), Toluene (1.5 mL), 130 °C, 40 h; **Isolated Yield:** 76 mg, 0.43 mmol, 86% (purified as white solid by flash column chromatography over silica gel with heptane/ethyl acetate = 2/1); **^{1}H NMR (300 MHz, DMSO-d$_6$) (major conformer):** δ (ppm): 9.83 (s, 1H), 8.67 (s, 1H), 6.90 (t, *J* = 7.7 Hz, 1H), 6.79 (dd, *J* = 8.1, 1.5 Hz, 1H), 6.65 (d, *J* = 7.3 Hz, 1H), 3.60 (t, *J* = 6.4 Hz, 2H), 2.65 (t, *J* = 6.5 Hz, 2H), 1.80 (quin, *J* = 6.4 Hz, 2H); **^{13}C NMR (75 MHz, DMSO-d$_6$) (major conformer):** δ (ppm): 163.4, 148.8, 133.1, 125.2, 124.8, 119.4, 114.4, 39.5, 26.6, 22.9 (N.B.: δ (ppm) = 39.5 was detected by DEPT analysis); **HR-MS (ESI):** *m/z* calculated for $[C_{10}H_{11}NO_2Na]^+$ ($[M+Na]^+$): 200.0682, found: 200.0681.

Methyl 1-formyl-1,2,3,4-tetrahydroquinoline-6-carboxylate (2k)

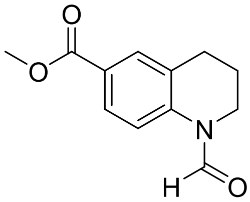

Reaction Conditions: methyl quinoline-6-carboxylate (72.6 mg, 0.50 mmol), HCOOH (10.0 equiv.), Co/Melamine-2@C-700 (60 mg), Toluene (1.5 mL), 130 °C, 24 h; **Isolated Yield:** 100 mg, 0.45 mmol, 90% (purified as white solid by flash column chromatography over silica gel with heptane/ethyl acetate = 4/1); **^{1}H NMR (300 MHz, CDCl$_3$) (major conformer):** δ (ppm): 8.83 (s, 1H), 7.78 - 7.79 (m, 2H), 7.15 - 7.18 (m, 1H), 3.84 (s, 3H), 3.76 (t, *J* = 6.0 Hz, 2H), 2.79 (t, *J* = 6.4 Hz, 2H), 1.91 (quin, *J* = 6.3 Hz, 2H); **^{13}C NMR (75 MHz, CDCl$_3$) (major conformer):** δ (ppm): 166.4, 160.8, 141.2, 131.1, 128.6, 128.3, 125.8, 116.1, 52.0, 40.5, 27.2, 21.7; **HR-MS (ESI):** *m/z* calculated for $[C_{12}H_{13}NO_3Na]^+$ ($[M+Na]^+$): 242.0788, found: 242.0785.

7-Fluoro-2-methyl-3,4-dihydroquinoline-1(2*H*)-carbaldehyde (2l)

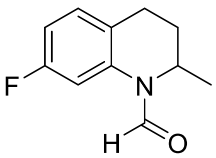

Reaction Conditions: 7-fluoro-2-methylquinoline (80.6 mg, 0.50 mmol), HCOOH (10.0 equiv.), Co/Melamine-2@C-700 (60 mg), Toluene (1.5 mL), 130 °C, 24 h; **Isolated Yield:** 90 mg, 0.47 mmol, 93% (purified as colorless oil by flash column chromatography over silica gel with heptane/ethyl acetate = 4/1); **^{1}H NMR (300 MHz, CDCl$_3$) (major conformer):** δ (ppm): 8.63 (s, 1H), 7.06 - 7.11 (m, 1H), 6.74 - 6.85 (m, 2H), 4.77 (sextet, *J* = 6.2 Hz, 1H), 2.72 - 2.83 (m, 1H), 2.58 - 2.67 (m, 1H), 1.97 – 2.08 (m, 1H), 1.62 – 1.72 (m, 1H), 1.16 (d, *J* = 6.6 Hz, 3H); **^{13}C NMR (75 MHz, CDCl$_3$) (major conformer):** δ (ppm): 161.5 (d, *J* = 244.5 Hz), 160.7, 137.2 (d, *J* = 9.7 Hz), 130.3 (d, *J* = 9.0 Hz), 124.7 (d, *J* = 3.2 Hz), 111.3 (d, *J* = 21.2 Hz), 105.3 (d, *J* = 25.2 Hz), 44.9, 28.6, 23.4, 17.9; **^{19}F NMR (282.4 MHz, CDCl$_3$):** δ (ppm): -113.85; **HR-MS (ESI):** *m/z* calculated for $[C_{11}H_{12}FNONa]^+$ ($[M+Na]^+$): 216.0795, found: 216.0796.

1-formyl-1,2,3,4-tetrahydroquinoline-6-carbonitrile (2m)

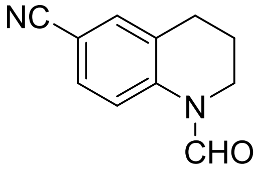

Reaction Conditions: quinoline-6-carbonitrile (81.0 mg, 0.50 mmol), HCOOH (10.0 equiv.), Co/Melamine-2@C-700 (60 mg), Toluene (1.5 mL), 130 °C, 24 h; **Isolated Yield:** 67.0 mg, 72% (purified as white solid by flash column chromatography over silica gel with hexane/ethyl acetate = 2/1); **^{1}H NMR (400 MHz, CDCl$_3$) (major conformer):** δ (ppm): 8.91 (s, 1H), 7.47-7.54 (m, 2H), 7.30 (d, *J* = 8.4 Hz, 1H),3.86 (t, *J* = 6.0 Hz, 2H), 2.88 (t, *J* = 6.4 Hz, 2H), 2.01 (t, *J* = 6.4 Hz, 2H); **^{13}C NMR (100 MHz, CDCl$_3$) (major conformer):** δ (ppm): 160.6, 141.2, 133.4, 131.1, 129.4, 118.5, 116.8, 107.5, 40.5, 27.0, 21.3; **HR-MS (ESI):** *m/z* calculated for $[C_{11}H_{10}N_2ONa]^+$ ($[M+Na]^+$): 209.0685, found: 209.0686.

1-formyl-1,2,3,4-tetrahydroquinoline-6-carboxylic acid (2n)

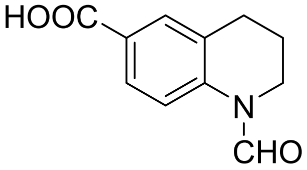

Reaction Conditions: quinoline-6-carboxylic acid (86.5 mg, 0.50 mmol), HCOOH (10.0 equiv.), Co/Melamine-2@C-700 (60 mg), Toluene (1.5 mL), 130 °C, 24 h; **Isolated Yield:** 70.8 mg, 65% (purified as white solid by flash column chromatography over silica gel with ethyl acetate); **^{1}H NMR (300 MHz, DMSO-d$_6$) (major conformer):** δ (ppm): 12.8 (s, 1H), 8.98 (s, 1H), 7.71-7.79 (m, 2H),

7.54 (d, *J* = 8.7 Hz, 1H), 3.70 (t, *J* = 6.0 Hz, 2H), 2.82 (t, *J* = 6.3 Hz, 2H), 1.85 (quintet, *J* = 6.3 Hz, 2H); **^{13}C NMR (75 MHz, DMSO-d$_6$) (major conformer):** δ (ppm): 166.9, 161.5, 141.1, 130.7, 128.2, 127.9, 125.7, 116.4, 40.0, 26.9, 21.2; **HR-MS (EI):** *m/z* calculated for $[C_{11}H_{11}NO_3]^+$ ($[M]^+$): 205.07334, found: 205.07452.

1-formyl-1,2,3,4-tetrahydroquinolin-6-yl trifluoromethanesulfonate (2o)

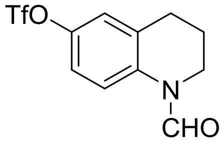

Reaction Conditions: quinolin-6-yl trifluoromethanesulfonate (141.5 mg, 0.5 mmol), HCOOH (15.0 equiv.), Co/Melamine-2@C-700 (100 mg), Toluene (1.5 mL), 130 °C, 50 h; **Isolated Yield:** 108.5 mg, 70% (purified as colorless oil by flash column chromatography using Combi Flash Rf+); **^{1}H NMR (300 MHz, CDCl$_3$) (major conformer):** δ (ppm): 8.78 (s, 1H), 7.16-7.23 (m, 1H), 7.08-7.16 (m, 2H), 3.77-3.88 (m, 2H), 2.85 (t, *J* = 6.3 Hz, 2H), 1.98 (quintet, *J* = 6.3 Hz, 2H); **^{13}C NMR (75 MHz, CDCl$_3$) (major conformer):** δ (ppm): 160.8, 145.8, 137.4, 131.1, 122.4, 120.0, 118.2, 40.2, 27.2, 21.6; **HR-MS (EI):** *m/z* calculated for $[C_{11}H_{10}F_3NO_4S]^+$ ($[M]^+$): 309.02771, found: 309.02705.

2-Methyl-3,4-dihydroquinoline-1(2*H*)-carbaldehyde (2p)

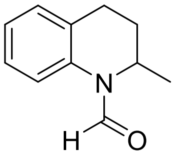

Reaction Conditions: 2-methylquinoline (71.6 mg, 0.50 mmol), HCOOH (10.0 equiv.), Co/Melamine-2@C-700 (60 mg), Toluene (1.5 mL), 130 °C, 24 h; **Isolated Yield:** 87 mg, 0.50 mmol, 99% (purified as colorless oil by flash column chromatography over silica gel with heptane/ethyl acetate = 4/1); **^{1}H NMR (300 MHz, CDCl$_3$) (major conformer):** δ (ppm): 8.67 (s, 1H), 7.09 - 7.24 (m, 4H), 4.80 (sextet, *J* = 6.3 Hz, 1H), 2.83 (ddd, *J* = 16.4, 9.1, 5.5 Hz, 1H), 2.69 (ddd, *J* = 16.3, 6.4, 5.3 Hz, 1H), 2.07 – 2.18 (m, 1H), 1.64 – 1.74 (m, 1H), 1.21 (d, *J* = 6.6 Hz, 3H); **^{13}C NMR (75 MHz, CDCl$_3$) (major conformer):** δ (ppm): 161.2, 136.3, 129.9, 129.2, 127.3, 124.9, 118.5, 45.3, 29.3, 24.3, 18.2; **GC-MS: EI-MS [*m/z* (%)]:** (M^+ = 175) major peaks found: 176 (10), 175 (81), 160 (62), 146 (19), 133 (11), 132 (100), 131 (14), 130 (24), 117 (23), 104 (104), 77 (19). The obtained spectroscopic data were in agreement with the reported data for this compound [ref.1]

4-methyl-3,4-dihydroquinoline-1(2H)-carbaldehyde (2q)

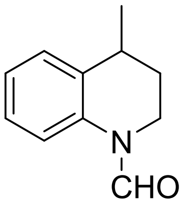

Reaction Conditions: 4-methylquinoline (73.8 mg, 0.50 mmol), HCOOH (15.0 equiv.), Co/Melamine-2@C-700 (120 mg), Toluene (1.5 mL), 130 °C, 24 h; **Isolated Yield:** 87.2 mg, 98% (purified as colorless oil by flash column chromatography over silica gel with hexane/ethyl acetate = 3/1); **^{1}H NMR (300 MHz, $CDCl_3$):** δ (ppm): 8.82 (s, 1H), 7.15-7.33 (m, 4H), 3.76-3.95 (m, 2H), 2.88-3.03 (m, 1H), 2.03-2.15 (m, 1H), 1.65-1.78 (m, 1H), 1.37 (d, J = 6.9 Hz, 3H); **^{13}C NMR (75 MHz, $CDCl_3$):** δ (ppm): 161.1, 136.6, 134.0, 128.1, 127.1, 124.7, 117.2, 38.2, 30.7, 30.0, 20.8; **HR-MS (ESI):** *m/z* calculated for $[C_{11}H_{13}NONa]^+$ ($[M+Na]^+$): 198.0889, found: 198.0889.

3-methyl-3,4-dihydroquinoline-1(2H)-carbaldehyde (2r)

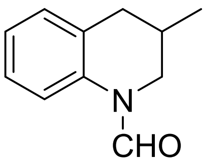

Reaction Conditions: 3-methylquinoline (73.0 mg, 0.50 mmol), HCOOH (10.0 equiv.), Co/Melamine-2@C-700 (60 mg), Toluene (1.5 mL), 130 °C, 24 h; **Isolated Yield:** 87.8 mg, 99% (purified as colorless oil by flash column chromatography over silica gel with hexane/ethyl acetate = 4/1); **^{1}H NMR (300 MHz, $CDCl_3$):** δ (ppm): 8.74 (s, 1H), 6.92-7.18 (m, 4H), 4.10 (ddd, J = 12.9, 4.5, 1.5 Hz, 1H), 3.02 (dd, J = 12.6, 10.2 Hz, 1H), 2.83 (ddd, J = 16.2, 5.1, 1.5 Hz, 1H), 2.40 (dd, J = 16.2, 9.9 Hz, 1H), 1.84-2.06 (m, 1H), 1.02 (d, J = 6.6 Hz, 3H); **^{13}C NMR (75 MHz, $CDCl_3$):** δ (ppm): 161.1, 136.9, 129.8, 128.3, 127.0, 124.6, 116.7, 46.4, 35.5, 27.9, 18.7; **HR-MS (ESI):** *m/z* calculated for $[C_{11}H_{13}NONa]^+$ ($[M+Na]^+$): 198.0889, found: 198.0891.

3-oxo-3,4-dihydroquinoline-1(2H)-carbaldehyde (2s)

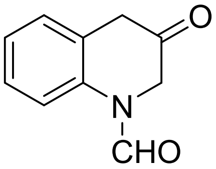

Reaction Conditions: quinolin-3-ol (72.0 mg, 0.50 mmol), HCOOH (10.0 equiv.), Co/Melamine-2@C-700 (60 mg), Toluene (1.5 mL), 130 °C, 24 h; **Isolated Yield:** 56.0 mg, 64% (purified as white solid by flash column chromatography using Combi Flash Rf+); **^{1}H NMR (300 MHz, $CDCl_3$):** δ (ppm): 8.59 (s, 1H), 7.14-7.33 (m, 4H), 4.26 (s, 2H), 3.57 (s, 2H); **^{13}C NMR (75 MHz, $CDCl_3$):** δ (ppm): 204.1, 160.7, 136.5, 129.3, 128.6, 127.1, 126.4, 120.2, 49.0, 43.9; **HR-MS (ESI):** *m/z* calculated for $[C_{10}H_9NO_2Na]^+$ ($[M+Na]^+$): 198.0526, found: 198.0529.

3-acetyl-3,4-dihydroquinoline-1(2H)-carbaldehyde (2t)

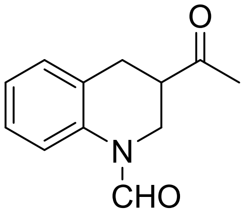

Reaction Conditions: 1-(quinolin-3-yl)ethan-1-one (88.2 mg, 0.50 mmol), HCOOH (10.0 equiv.), Co/Melamine-2@C-700 (60 mg), Toluene (1.5 mL), 130 °C, 24 h; **Isolated Yield:** 73.0 mg, 72% (purified as white solid by flash column chromatography over silica gel with hexane/ethyl acetate = 3/1); **^{1}H NMR (400 MHz, $CDCl_3$):** δ (ppm): 8.79 (s, 1H), 7.13-7.28 (m, 4H), 4.29-4.42 (m, 1H), 3.64-3.73 (m, 1H), 2.99-3.12 (m, 3H), 2.33 (s, 3H),; **^{13}C NMR (100 MHz, $CDCl_3$):** δ (ppm): 207.1, 160.9, 136.5, 129.8, 127.3, 126.5, 125.0, 117.0, 45.9, 40.4, 28.6, 28.5; **HR-MS (ESI):** *m/z* calculated for $[C_{12}H_{13}NO_2Na]^+$ ($[M+Na]^+$): 226.0839, found: 226.0839.

N-(1-formyl-1,2,3,4-tetrahydroquinolin-3-yl)formamide (2u)

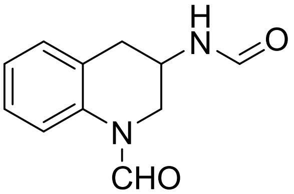

Reaction Conditions: quinolin-3-amine (73.5 mg, 0.50 mmol), HCOOH (10.0 equiv.), Co/Melamine-2@C-700 (60 mg), Toluene (1.5 mL), 130 °C, 24 h; **Isolated Yield:** 68.0 mg, 66% (purified as white solid by flash column chromatography over silica gel with ethyl acetate); **^{1}H NMR (300 MHz, CDCl$_3$) (major conformer):** δ (ppm): 8.66 (s, 1H), 7.97 (s, 1H), 7.04-7.16 (m, 4H), 6.53 (d, *J* = 8.1 Hz, 1H), 4.35-4.46 (m, 1H), 3.91 (dd, *J* = 13.2, 6.0 Hz, 1H), 3.61 (dd, *J* = 13.2, 3.9 Hz, 1H), 3.05 (dd, *J* = 16.8, 5.1 Hz, 1H), 2.77 (dd, *J* = 16.8, 5.7 Hz, 1H); **^{13}C NMR (75 MHz, CDCl$_3$) (major conformer):** δ (ppm): 161.3, 161.0, 136.2, 130.4, 127.6, 125.1, 116.7, 43.4, 41.5, 32.8; **HR-MS (ESI):** *m/z* calculated for $[C_{11}H_{12}N_2O_2Na]^+$ ($[M+Na]^+$): 227.0791, found: 227.0793.

2-Phenyl-3,4-dihydroquinoline-1(2H)-carbaldehyde (2v)

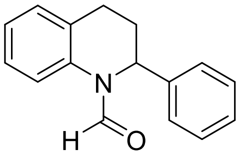

Reaction Conditions: 2-phenylquinoline (102.6 mg, 0.50 mmol), HCOOH (15.0 equiv.), CoOx-Melamine@C-700 (60 mg), Toluene (1.5 mL), 130 °C, 40 h; **Isolated Yield:** 114 mg, 0.48 mmol, 96% (purified as colorless oil by flash column chromatography over silica gel with heptane/ethyl acetate = 4/1); **^{1}H NMR (300 MHz, CDCl$_3$) (major conformer):** δ (ppm): 8.84 (s, 1H), 7.11 - 7.28 (m, 9H), 5.75 (t, *J* = 6.2 Hz, 1H), 2.64 - 2.69 (m, 2H), 2.30 - 2.41 (m, 1H), 2.06 - 2.16 (m, 1H); **^{13}C NMR (75 MHz, CDCl$_3$) (major conformer):** δ (ppm): 161.5, 141.0, 137.1, 130.1, 129.1, 128.4, 127.4, 126.9, 125.8, 124.7, 117.8, 53.3, 30.0, 24.6; **HR-MS (ESI):** *m/z* calculated for $[C_{16}H_{15}NONa]^+$ ($[M+Na]^+$): 260.1046, found: 260.1045.

2-(but-3-en-1-yl)-3,4-dihydroquinoline-1(2H)-carbaldehyde (2w)

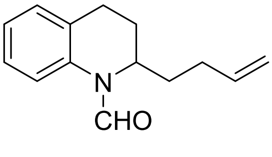

Reaction Conditions: 2-(but-3-en-1-yl)quinoline (85.0 mg, 0.465mmol), HCOOH (10.0 equiv.), Co/Melamine-2@C-700 (60 mg), Toluene (1.5 mL), 130 °C, 24 h; **Isolated Yield:** 68.2 mg, 68% (purified as colorless oil by flash column chromatography over silica gel with hexane/ethyl acetate = 3/1); **^{1}H NMR (400 MHz, CDCl$_3$) (major conformer):** δ (ppm): 8.68 (s, 1H), 7.09-7.21 (m, 4H), 5.68-5.86 (m, 1H), 4.90-5.04 (m, 2H), 4.75-4.83 (m, 1H), 2.71-2.85 (m, 2H), 2.00-2.12 (m, 3H), 1.80-1.86 (m, 1H), 1.64-1.72 (m, 1H), 1.45-1.54 (m, 1H); **^{13}C NMR (100 MHz, CDCl$_3$) (major

conformer): δ (ppm): 161.4, 137.7, 136.2, 129.4, 129.1, 127.1, 124.9, 118.6, 114.9, 47.9, 30.6, 30.1, 26.5, 23.6; **HR-MS (EI):** *m/z* calculated for $[C_{14}H_{17}NO]^+$ ($[M]^+$): 215.13047, found: 215.13037.

phenanthridine-5(6H)-carbaldehyde (5)

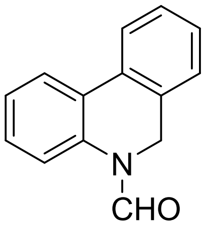

Reaction Conditions: phenanthridine (91.4 mg, 0.50 mmol), HCOOH (20.0 equiv.), Co/Melamine-2@C-700 (80 mg), Toluene (1.5 mL), 130 °C, 24 h; **Isolated Yield:** 100.0 mg, 95% (purified as white solid by flash column chromatography over silica gel with hexane/ethyl acetate = 3/1); **^{1}H NMR (300 MHz, $CDCl_3$) (major conformer):** δ (ppm): 8.50 (s, 1H), 7.68-7.82 (m, 2H), 7.11-7.32 (m, 6H), 4.84 (s, 2H); **^{13}C NMR (75 MHz, $CDCl_3$) (major conformer):** δ (ppm): 160.6, 136.2, 132.7, 130.7, 128.8, 128.3, 128.2, 127.8, 126.4, 126.3, 124.4, 123.3, 119.2, 42.1; **HR-MS (ESI):** *m/z* calculated for $[C_{14}H_{11}NONa]^+$ ($[M+Na]^+$): 232.0733, found: 232.0733.

3,4-dihydrobenzo[h]quinoline-1(2H)-carbaldehyde (7)

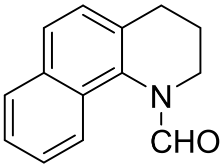

Reaction Conditions: benzo[h]quinoline (90.5 mg, 0.50 mmol), HCOOH (20.0 equiv.), Co/Melamine-2@C-700 (60 mg), Toluene (1.5 mL), 130 °C, 24 h; **Isolated Yield:** 94.0 mg, 89% (purified as white solid by flash column chromatography over silica gel with hexane/ethyl acetate = 3/1); **^{1}H NMR (300 MHz, $CDCl_3$) (major conformer):** δ (ppm): 8.65 (s, 1H), 7.94 (dd, J = 7.8, 1.5 Hz, 1H), 7.85 (dd, J = 7.5, 1.8 Hz, 1H), 7.67 (d, J = 8.1 Hz, 1H), 7.43-7.54 (m, 2H), 7.29 (d, J = 8.4 Hz, 1H), 3.94 (brs, 2H), 2.92 (t, J = 6.6 Hz, 2H), 2.07 (quintet, J = 6.6 Hz, 2H); **^{13}C NMR (75 MHz, $CDCl_3$) (major conformer):** δ (ppm): 164.3, 133.4, 133.0, 129.4, 128.4, 127.4, 126.8, 126.6, 125.7, 125.5, 122.0, 40.4, 27.0, 23.6; **HR-MS (ESI):** *m/z* calculated for $[C_{14}H_{13}NONa]^+$ ($[M+Na]^+$): 234.0889, found: 234.0890.

Phthalazine-2(1H)-carbaldehyde (9)

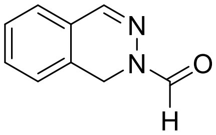

Reaction Conditions: phthalazine (65.1 mg, 0.50 mmol), HCOOH (15.0 equiv.), Co/Melamine-2@C-700 (60 mg), Toluene (1.5 mL), 130 °C, 27 h; **Isolated Yield:** 65 mg, 0.41 mmol, 81% (purified as white semisolid by flash column chromatography over silica gel with heptane/ethyl acetate = 4/1); **^{1}H NMR (300 MHz, CDCl$_3$):** δ (ppm): 8.66 (s, 1H), 7.48 (s, 1H), 7.39 (td, *J* = 7.5, 1.5 Hz, 1H), 7.27 - 7.33 (m, 1H), 7.20 (dd, *J* = 7.5, 1.2 Hz, 1H), 7.09 - 7.13 (m, 1H), 4.87 (s, 2H); **^{13}C NMR (75 MHz, CDCl$_3$):** δ (ppm): 164.7, 143.0, 132.0, 128.9, 128.4, 126.4, 126.1, 124.1, 41.0; **HR-MS (ESI):** *m/z* calculated for $[C_9H_8N_2ONa]^+$ ($[M+Na]^+$): 183.0529, found: 183.0527.

3,4-Dihydro-1,5-naphthyridine-1(2*H*)-carbaldehyde (11)

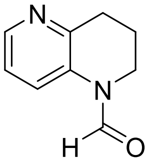

Reaction Conditions: 1,5-naphthyridine (67.0 mg, 0.50 mmol), HCOOH (10.0 equiv.), NEt_3 (2.5 equiv.), Co/Melamine-2@C-700 (60 mg), Toluene (1.5 mL), 130 °C, 24 h; **Isolated Yield:** 31 mg, 0.19 mmol, 38% (purified as colorless liquid by flash column chromatography over silica gel with dichloromethene/methanol = 97/3); **^{1}H NMR (400 MHz, CDCl$_3$) (major conformer):** δ (ppm): 8.74 (s, 1H), 8.29 - 8.32 (m, 1H), 7.47 (dd, *J* = 8.2, 1.3 Hz, 1H), 7.12 - 7.15 (m, 1H), 3.79 - 3.82 (m, 2H), 3.01 (t, *J* = 6.6 Hz, 2H), 2.02 (quin, *J* = 6.4 Hz, 2H); **^{13}C NMR (101 MHz, CDCl$_3$) (major and minor conformer):** δ (ppm): 161.9, 160.6, 149.1, 147.4, 145.2, 144.1, 133.9, 132.2, 130.1, 123.8, 122.1, 121.8, 45.8, 40.1, 30.4, 30.0, 22.3, 21.6; **HR-MS (ESI):** *m/z* calculated for $[C_9H_{11}N_2O]^+$ ($[M+H]^+$): 163.0866, found: 163.0868.

10. ^{1}H NMR, ^{13}C NMR and ^{19}F NMR Spectra of Substrate and Products

Substrate

1w

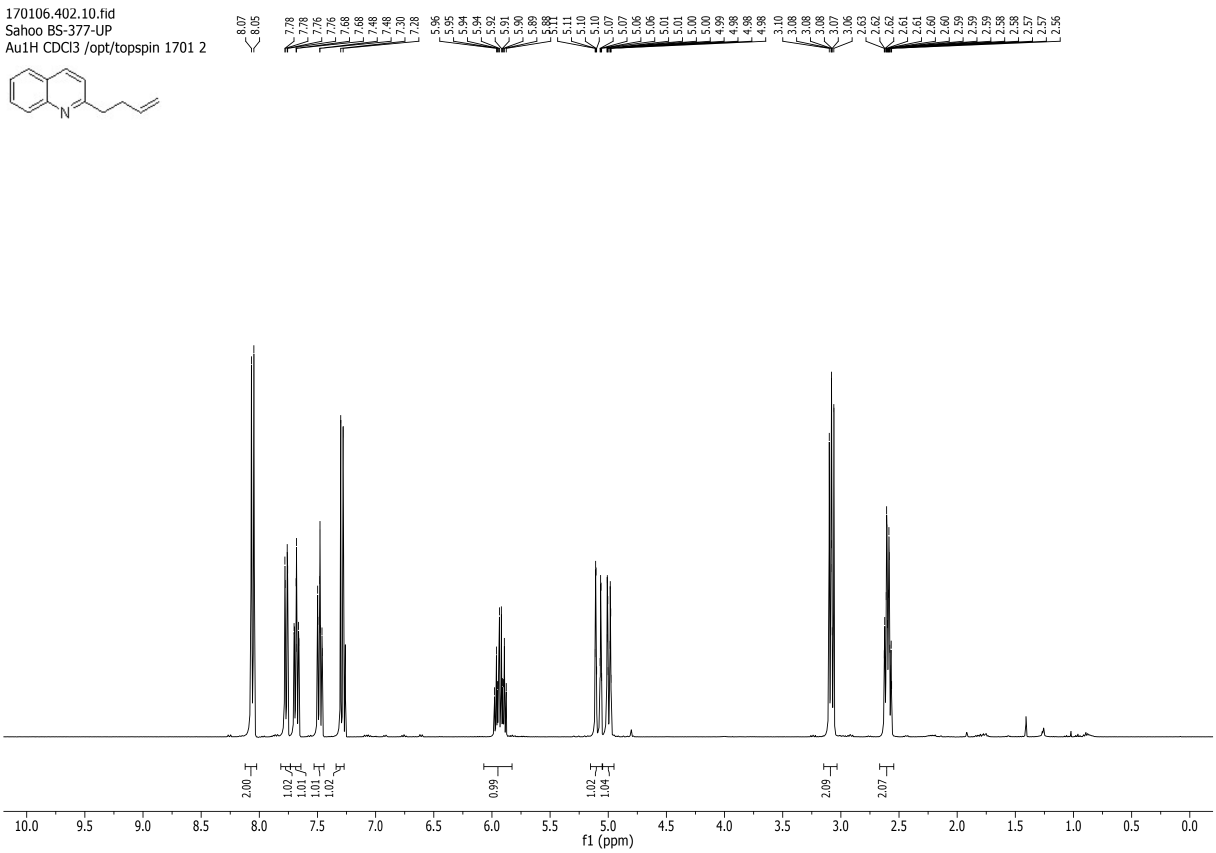

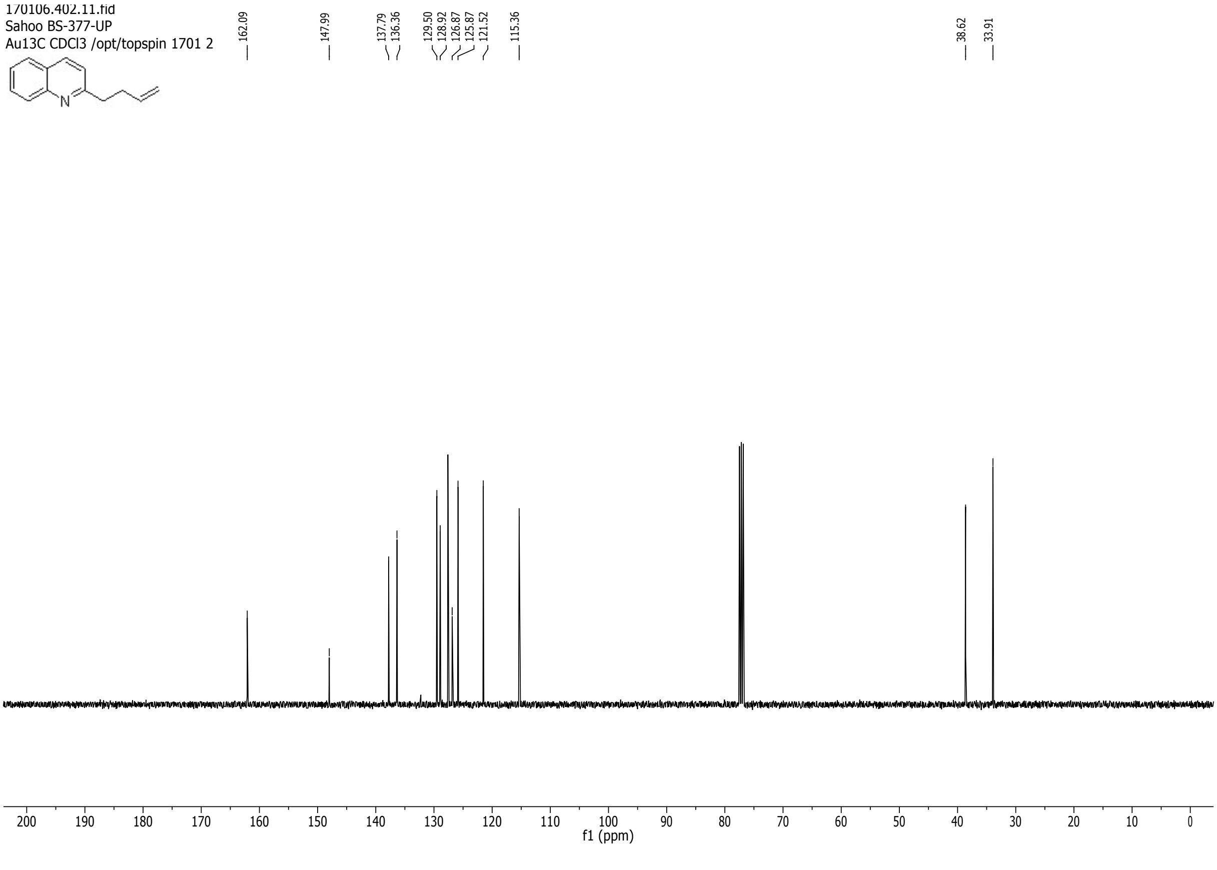

Products

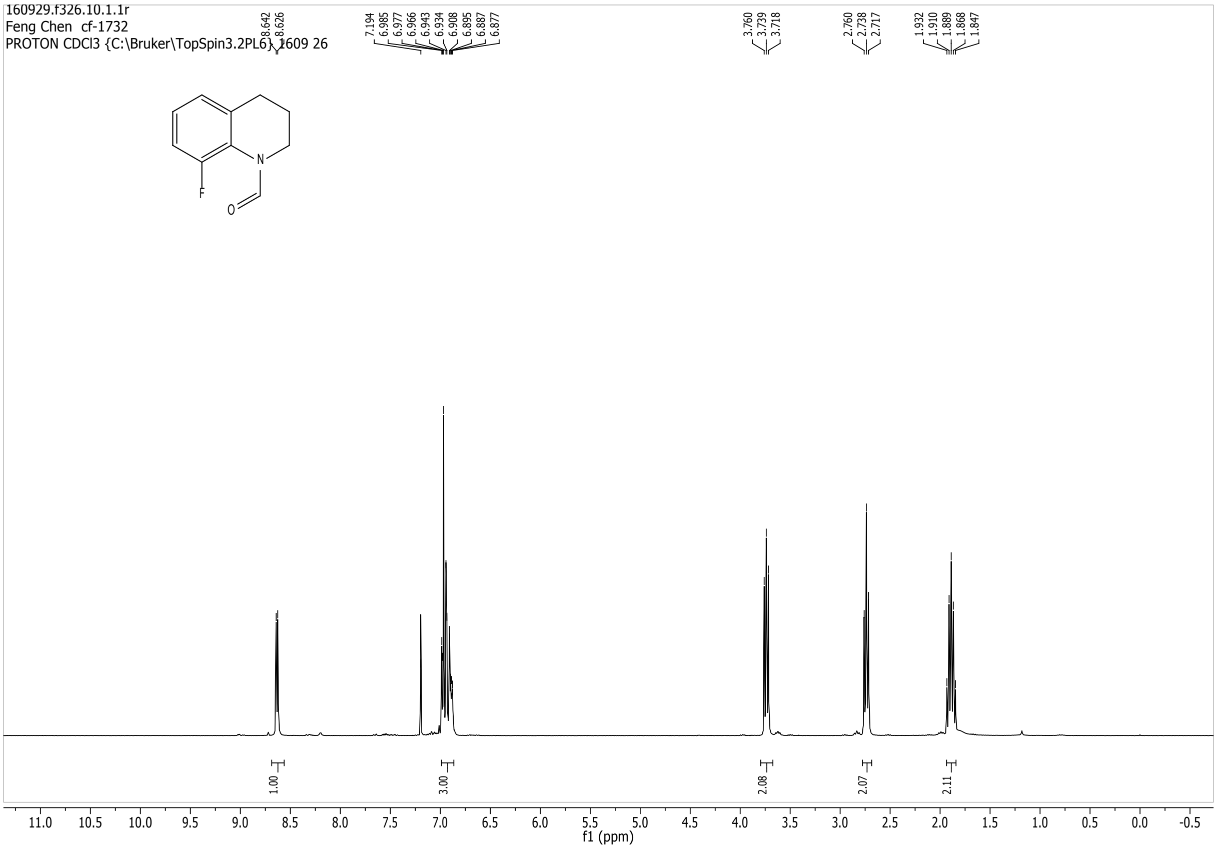
160929.f326.10.1.1r
Feng Chen cf-1732
PROTON CDCl3 {C:\Bruker\TopSpin3.2PL6} 1609 26
8.642
8.626
7.194
6.985
6.977
6.966
6.943
6.934
6.908
6.895
6.887
6.877
3.760
3.739
3.718
2.760
2.738
2.717
1.932
1.910
1.889
1.868
1.847
N
F
O
1.00
3.00
2.08
2.07
2.11
11.0 10.5 10.0 9.5 9.0 8.5 8.0 7.5 7.0 6.5 6.0 5.5 5.0 4.5 4.0 3.5 3.0 2.5 2.0 1.5 1.0 0.5 0.0 -0.5
f1 (ppm)

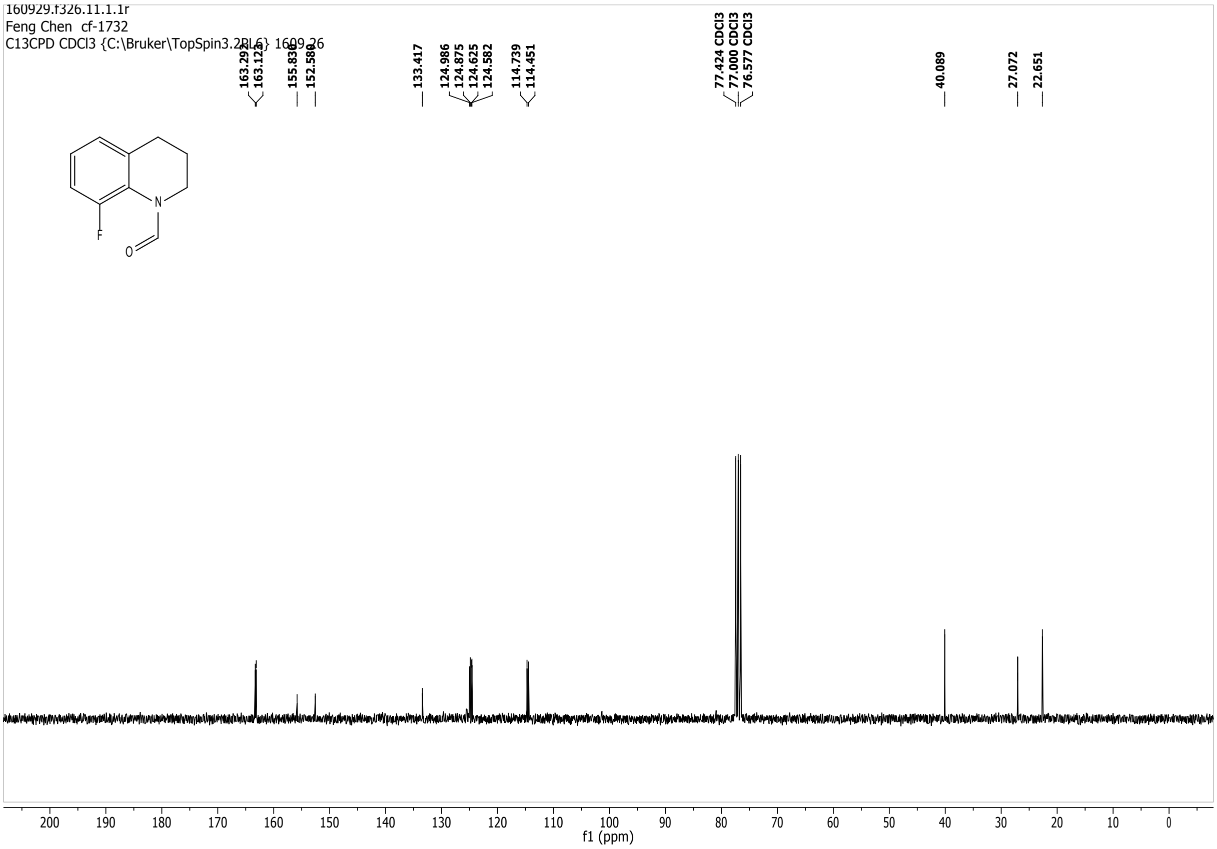
160929.f326.11.1.1r
Feng Chen cf-1732
C13CPD CDCl3 {C:\Bruker\TopSpin3.2PL6} 1609 26
163.29
163.12
155.83
152.58
133.417
124.986
124.875
124.625
124.582
114.739
114.451
77.424 CDCl3
77.000 CDCl3
76.577 CDCl3
40.089
27.072
22.651
N
F
O
200 190 180 170 160 150 140 130 120 110 100 90 80 70 60 50 40 30 20 10 0
f1 (ppm)

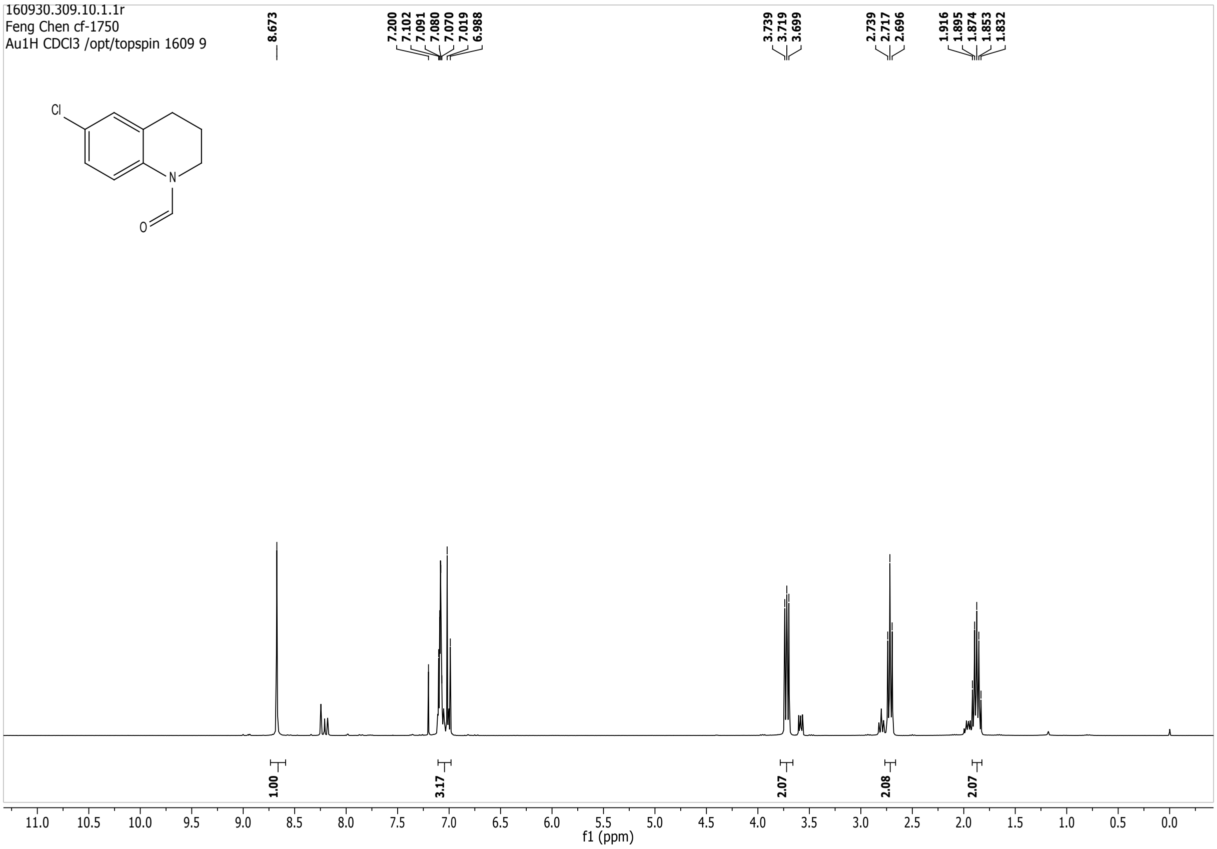
160930.309.10.1.1r
Feng Chen cf-1750
Au1H CDCl3 /opt/topspin 1609 9
Cl
N
O
8.673
7.200
7.102
7.091
7.080
7.070
7.019
6.988
3.739
3.719
3.699
2.739
2.717
2.696
1.916
1.895
1.874
1.853
1.832
1.00
3.17
2.07
2.08
2.07
11.0 10.5 10.0 9.5 9.0 8.5 8.0 7.5 7.0 6.5 6.0 5.5 5.0 4.5 4.0 3.5 3.0 2.5 2.0 1.5 1.0 0.5 0.0
f1 (ppm)

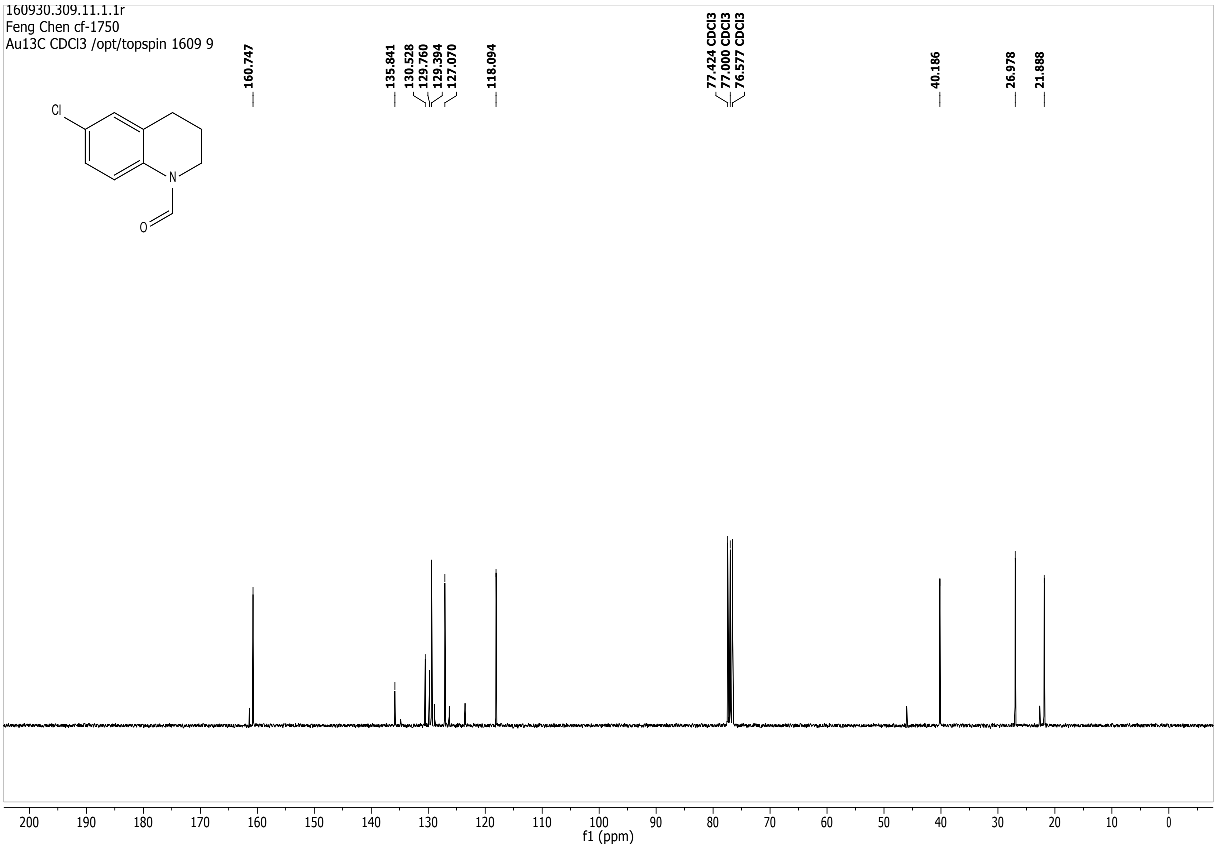
160930.309.11.1.1r
Feng Chen cf-1750
Au13C CDCl3 /opt/topspin 1609 9
Cl
N
O
160.747
135.841
130.528
129.760
129.394
127.070
118.094
77.424 CDCl3
77.000 CDCl3
76.577 CDCl3
40.186
26.978
21.888
200 190 180 170 160 150 140 130 120 110 100 90 80 70 60 50 40 30 20 10 0
f1 (ppm)

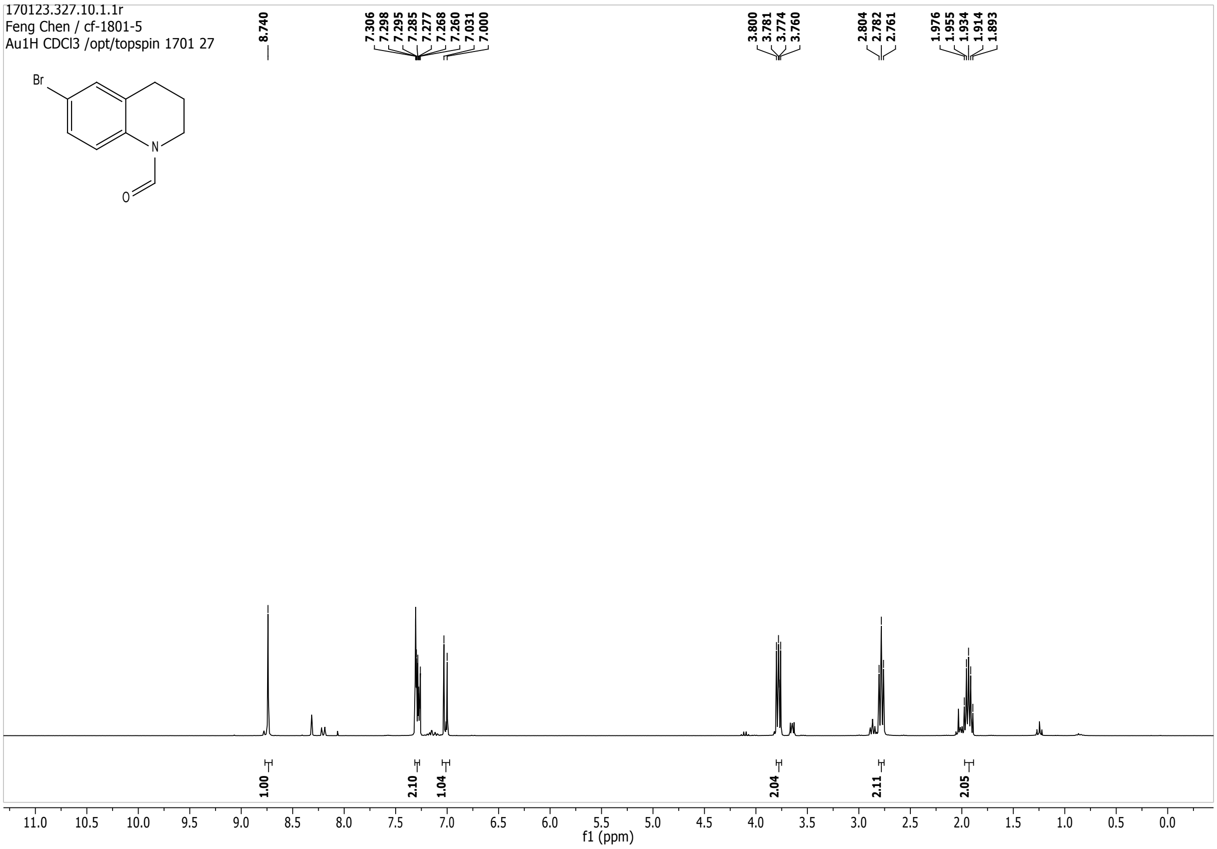
170123.327.10.1.1r
Feng Chen / cf-1801-5
Au1H CDCl3 /opt/topspin 1701 27
Br
N
O
8.740
7.306
7.298
7.295
7.285
7.277
7.268
7.260
7.031
7.000
3.800
3.781
3.774
3.760
2.804
2.782
2.761
1.976
1.955
1.934
1.914
1.893
1.00
2.10
1.04
2.04
2.11
2.05
f1 (ppm)

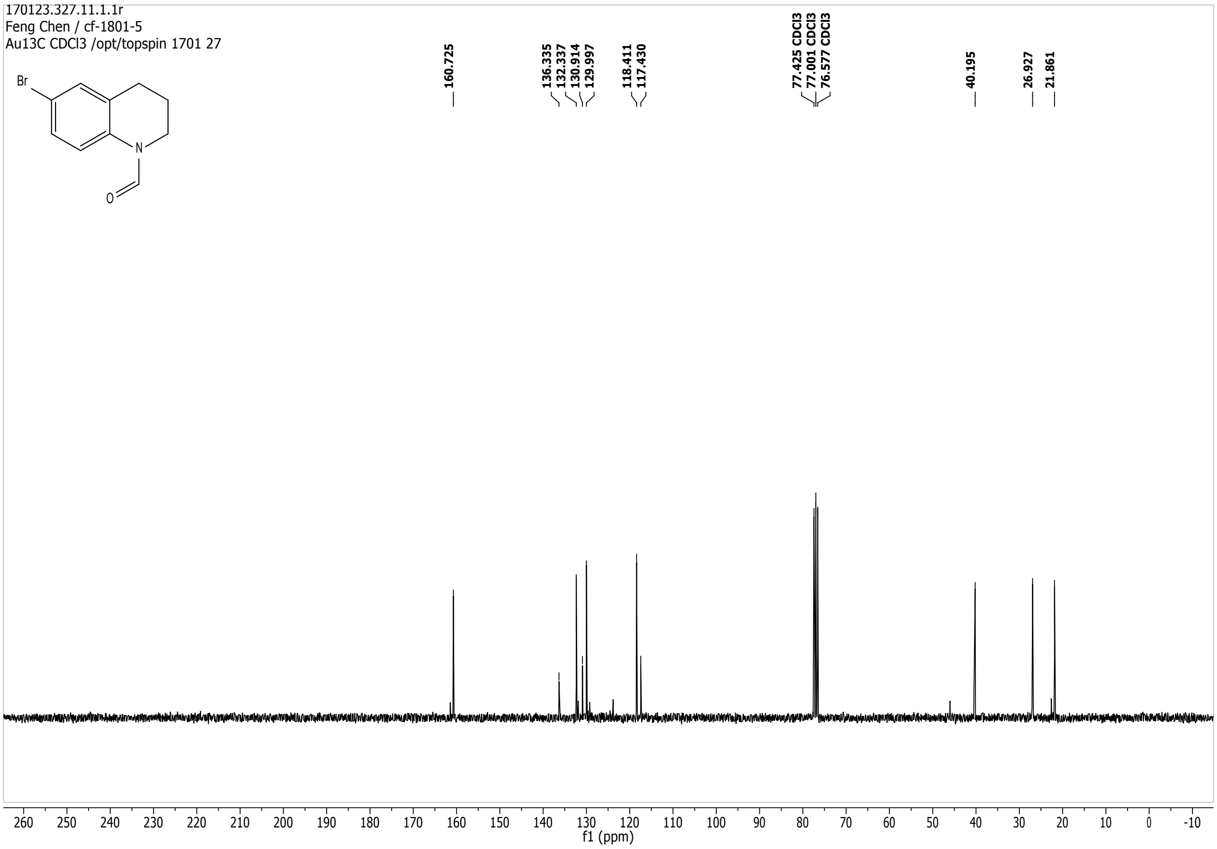
170123.327.11.1.1r
Feng Chen / cf-1801-5
Au13C CDCl3 /opt/topspin 1701 27
Br
N
O
160.725
136.335
132.337
130.914
129.997
118.411
117.430
77.425 CDCl3
77.001 CDCl3
76.577 CDCl3
40.195
26.927
21.861
f1 (ppm)

160930.f330.10.fid
Sahoo BS-344-PA
PROTON CDCl3 {C:\Bruker\TopSpin3.2PL6} 1609 30

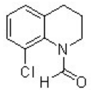

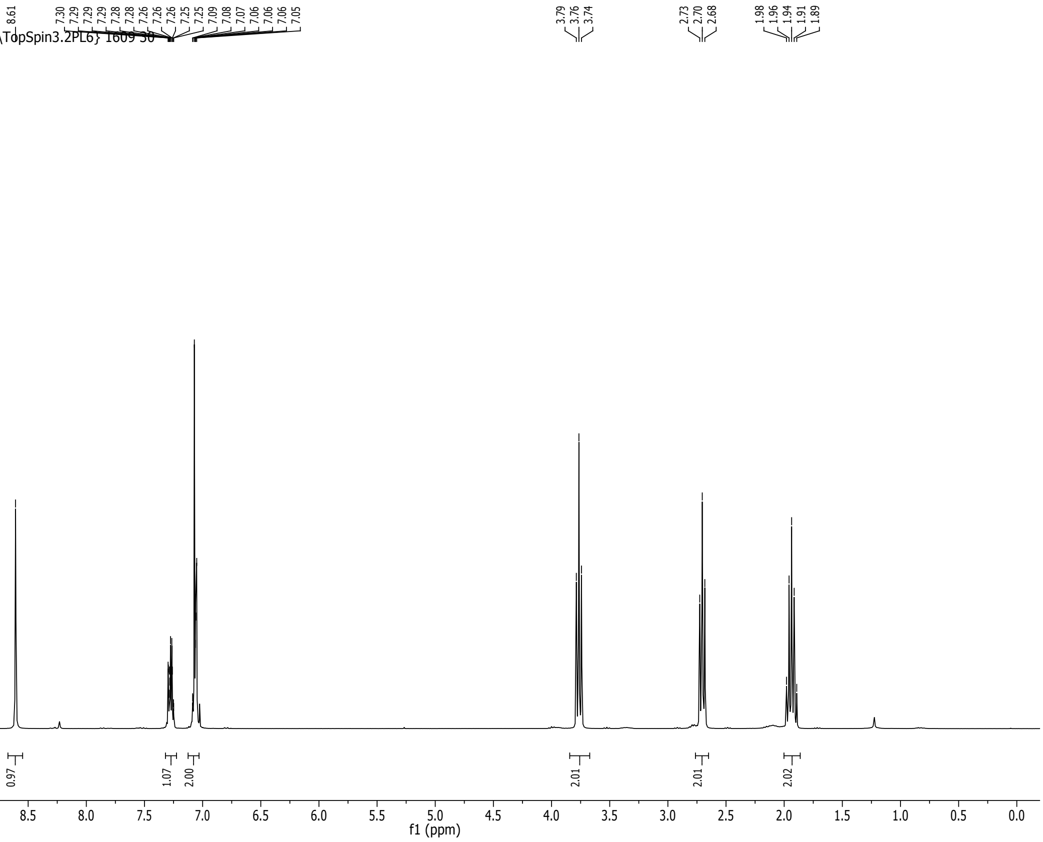

160930.f330.11.fid
Sahoo BS-344-PA
C13CPD CDCl3 {C:\Bruker\TopSpin3.2PL6} 1609 30

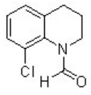

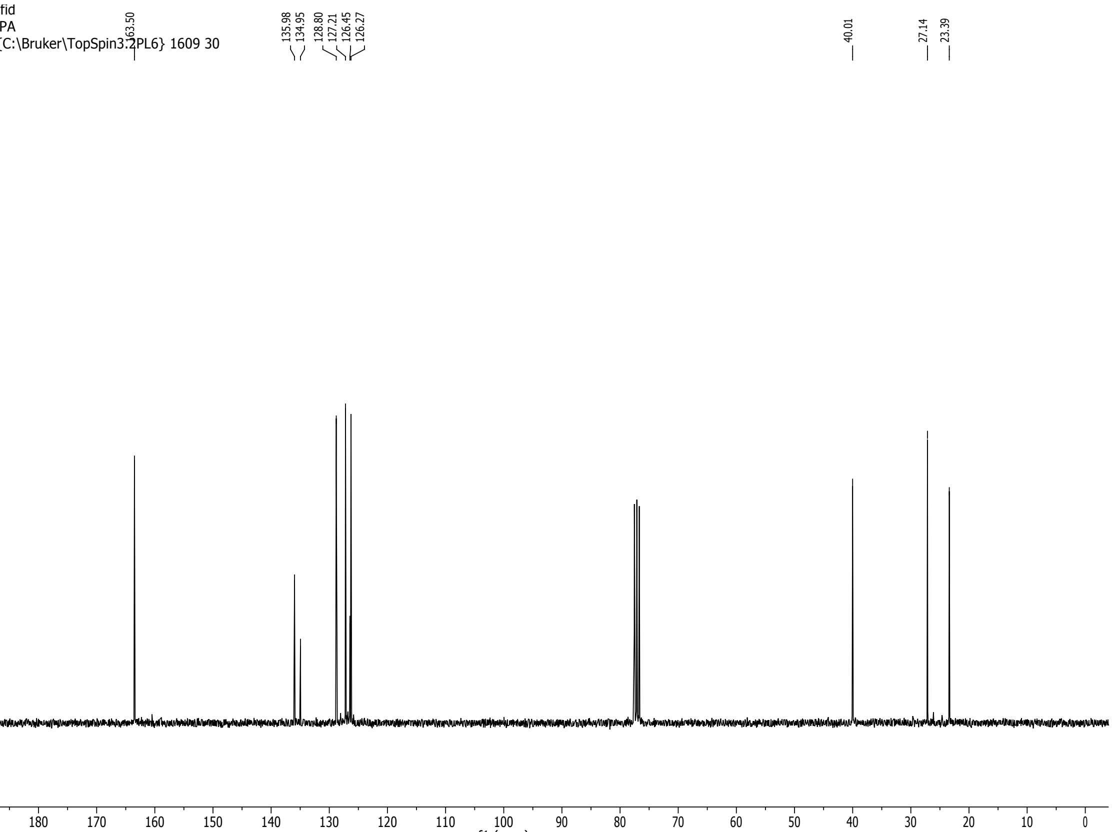

160930.f329.10.fid
Sahoo BS-344-BP
PROTON CDCl3 {C:\Bruker\TopSpin3.2PL6} 1609 29

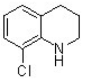

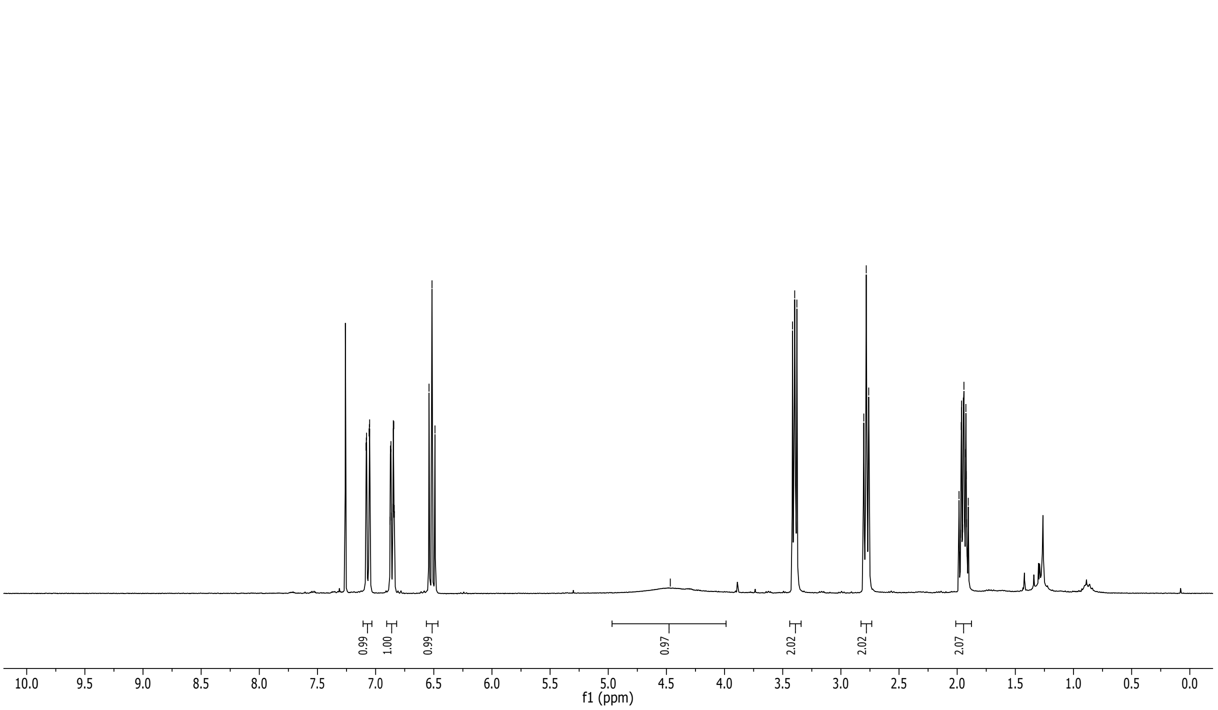

160930.f329.11.fid
Sahoo BS-344-BP
C13CPD CDCl3 {C:\Bruker\TopSpin3.2PL6} 1609 29

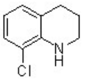

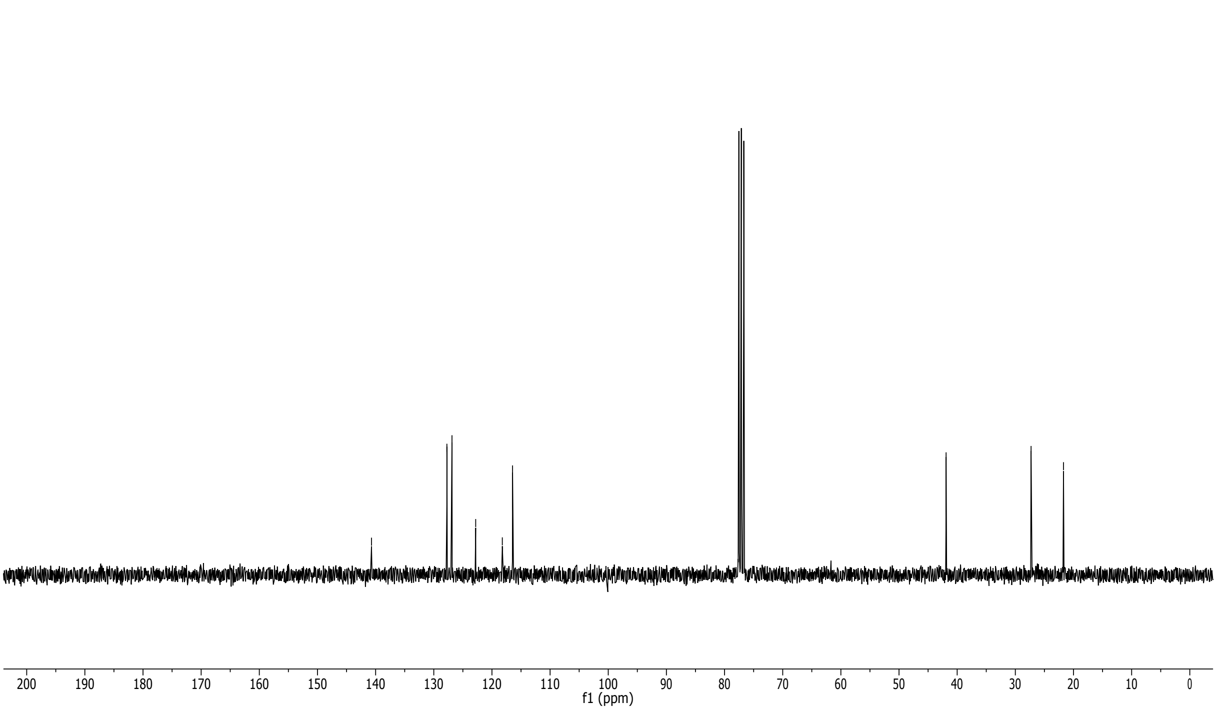

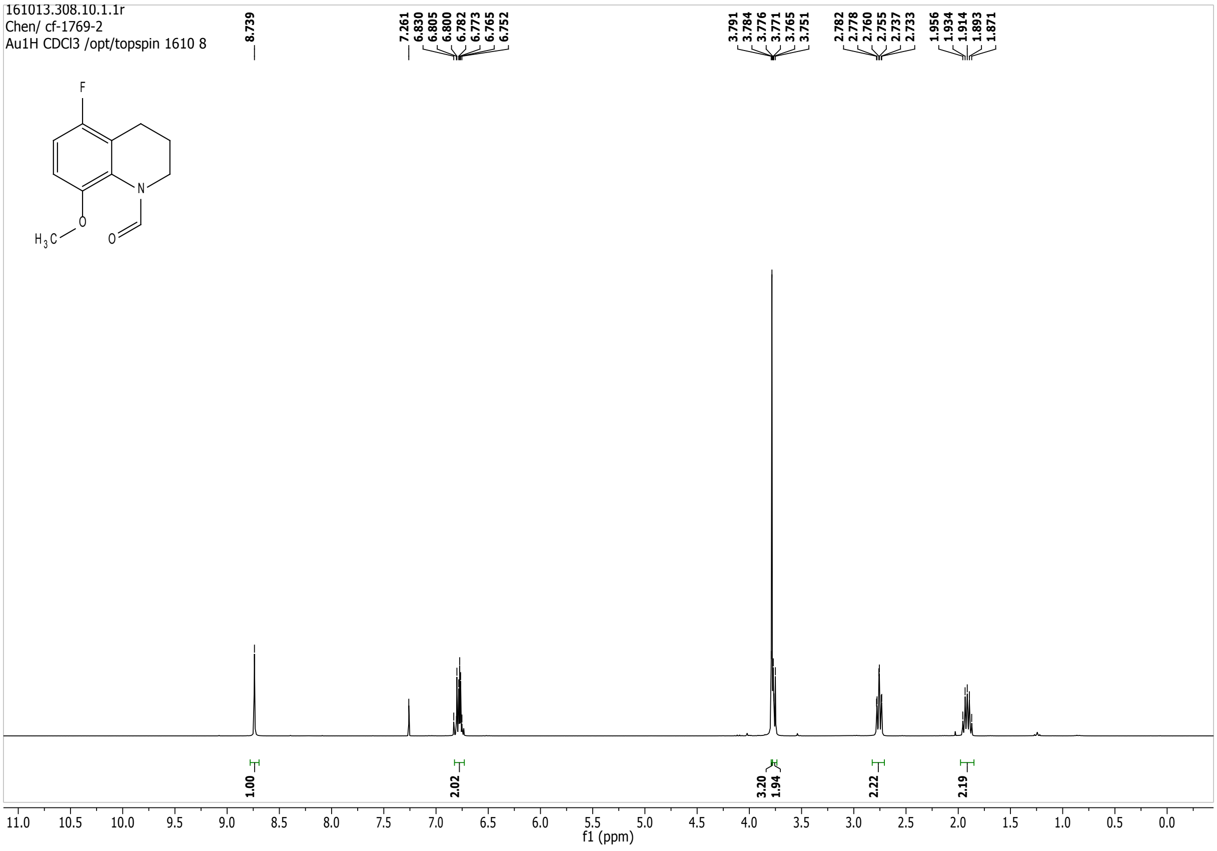
161013.308.10.1.1r
Chen/ cf-1769-2
Au1H CDCl3 /opt/topspin 1610 8
8.739
7.261
6.830
6.805
6.800
6.782
6.773
6.765
6.752
3.791
3.784
3.776
3.771
3.765
3.751
2.782
2.778
2.760
2.755
2.737
2.733
1.956
1.934
1.914
1.893
1.871
F
N
O
H3C
O
1.00
2.02
3.20
1.94
2.22
2.19
11.0 10.5 10.0 9.5 9.0 8.5 8.0 7.5 7.0 6.5 6.0 5.5 5.0 4.5 4.0 3.5 3.0 2.5 2.0 1.5 1.0 0.5 0.0
f1 (ppm)

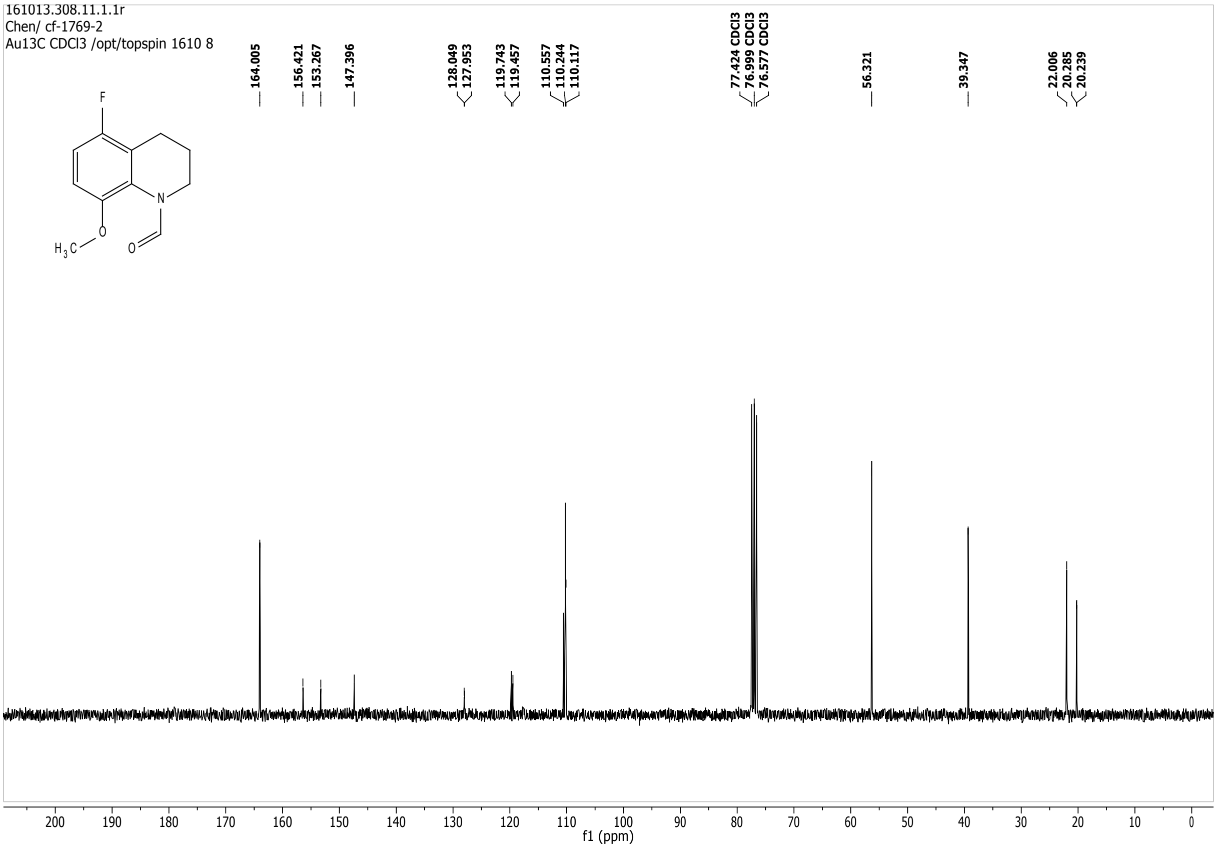
161013.308.11.1.1r
Chen/ cf-1769-2
Au13C CDCl3 /opt/topspin 1610 8
164.005
156.421
153.267
147.396
128.049
127.953
119.743
119.457
110.557
110.244
110.117
77.424 CDCl3
76.999 CDCl3
76.577 CDCl3
56.321
39.347
22.006
20.285
20.239
F
N
O
H3C
O
200 190 180 170 160 150 140 130 120 110 100 90 80 70 60 50 40 30 20 10 0
f1 (ppm)

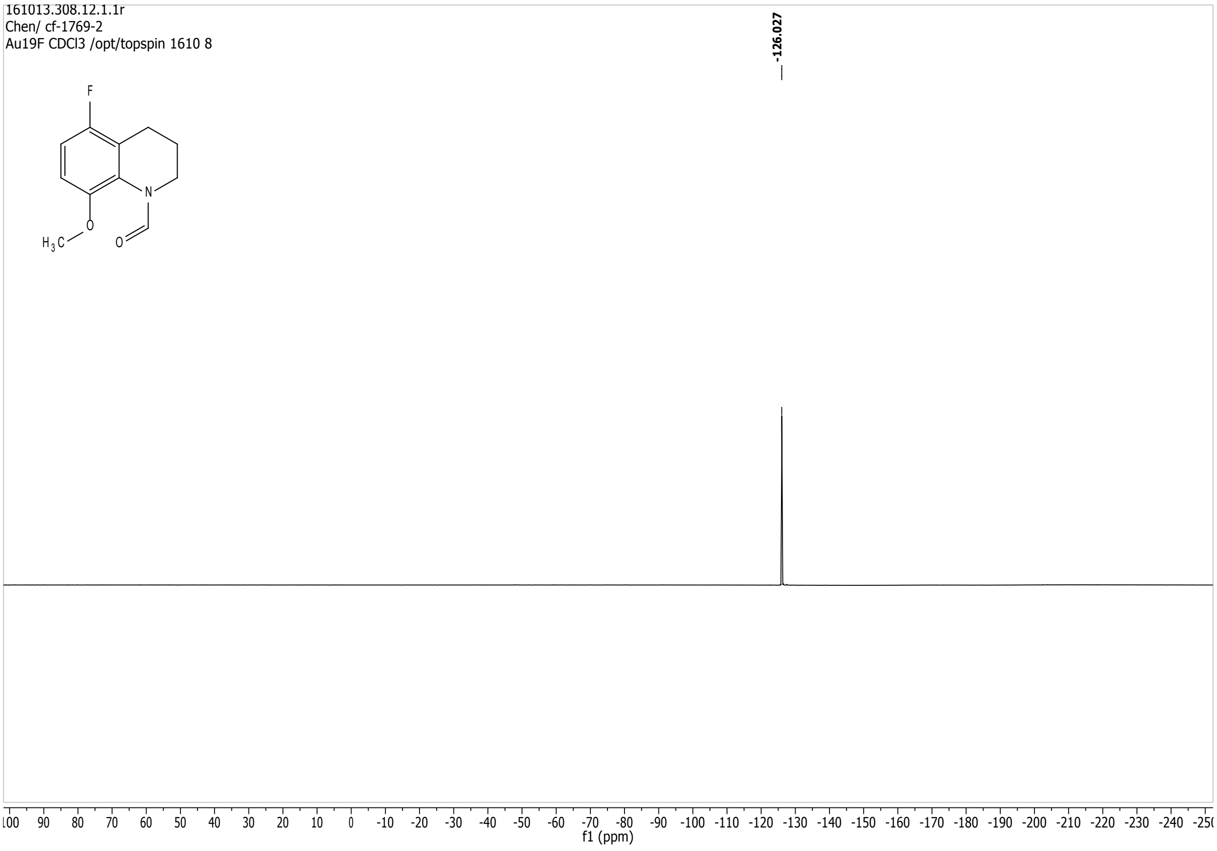
161013.308.12.1.1r
Chen/ cf-1769-2
Au19F CDCl3 /opt/topspin 1610 8
-126.027
F
N
O
H3C
O
f1 (ppm)

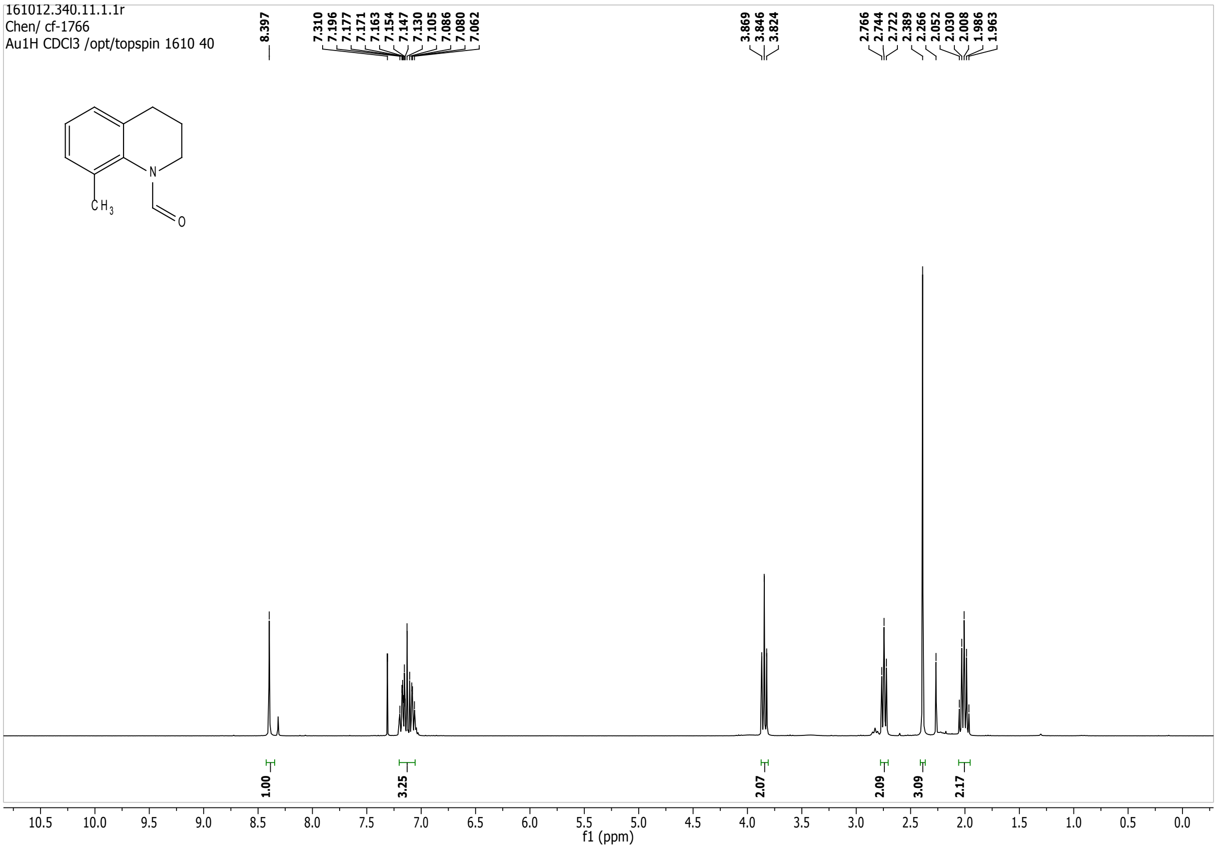
161012.340.11.1.1r
Chen/ cf-1766
Au1H CDCl3 /opt/topspin 1610 40
8.397
7.310 7.196 7.177 7.171 7.163 7.154 7.147 7.130 7.105 7.086 7.080 7.062
3.869 3.846 3.824
2.766 2.744 2.722 2.389 2.266 2.052 2.030 2.008 1.986 1.963
CH3
N
O
1.00
3.25
2.07
2.09
3.09
2.17
10.5 10.0 9.5 9.0 8.5 8.0 7.5 7.0 6.5 6.0 5.5 5.0 4.5 4.0 3.5 3.0 2.5 2.0 1.5 1.0 0.5 0.0
f1 (ppm)

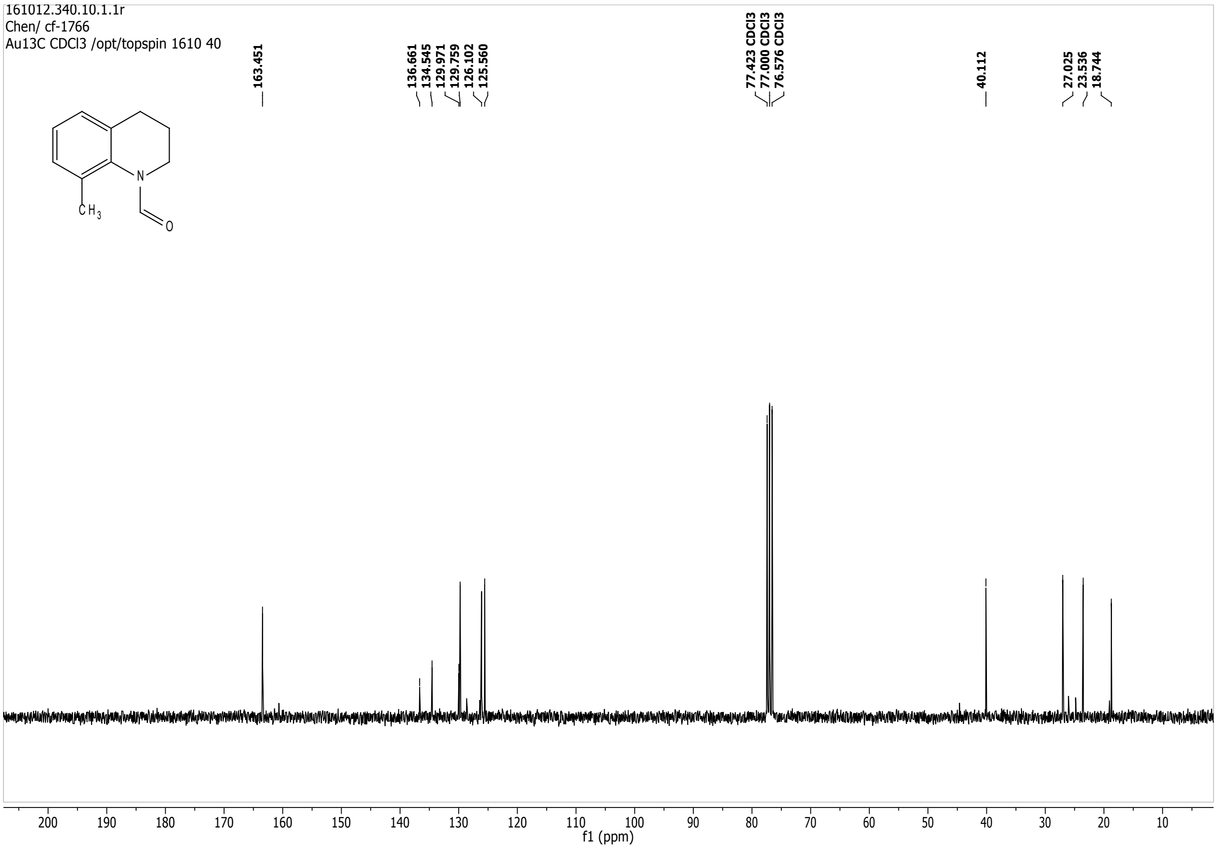
161012.340.10.1.1r
Chen/ cf-1766
Au13C CDCl3 /opt/topspin 1610 40
163.451
136.661 134.545 129.971 129.759 126.102 125.560
77.423 CDCl3 77.000 CDCl3 76.576 CDCl3
40.112
27.025 23.536 18.744
CH3
N
O
200 190 180 170 160 150 140 130 120 110 100 90 80 70 60 50 40 30 20 10
f1 (ppm)

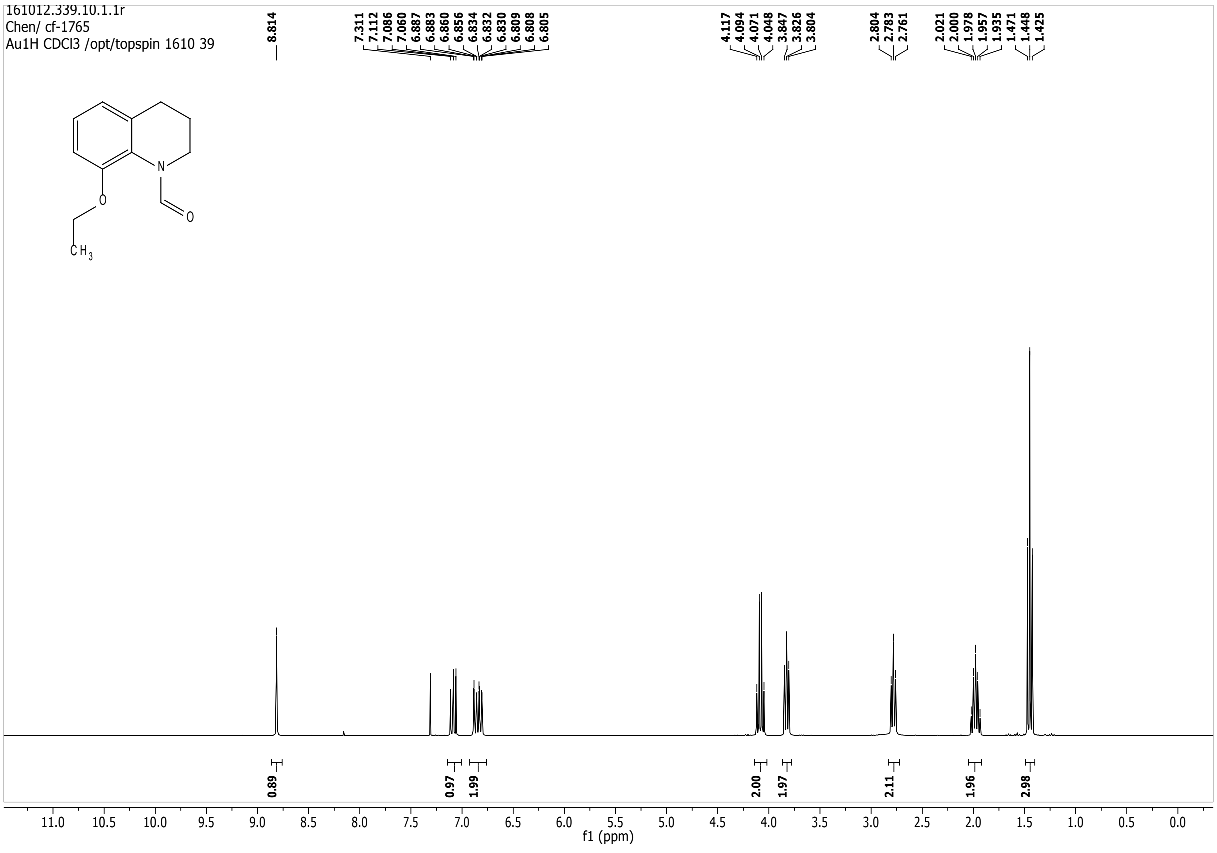
161012.339.10.1.1r
Chen/ cf-1765
Au1H CDCl3 /opt/topspin 1610 39
8.814
7.311
7.112
7.086
7.060
6.887
6.883
6.860
6.856
6.834
6.832
6.830
6.809
6.808
6.805
4.117
4.094
4.071
4.048
3.847
3.826
3.804
2.804
2.783
2.761
2.021
2.000
1.978
1.957
1.935
1.471
1.448
1.425
0.89
0.97
1.99
2.00
1.97
2.11
1.96
2.98
f1 (ppm)

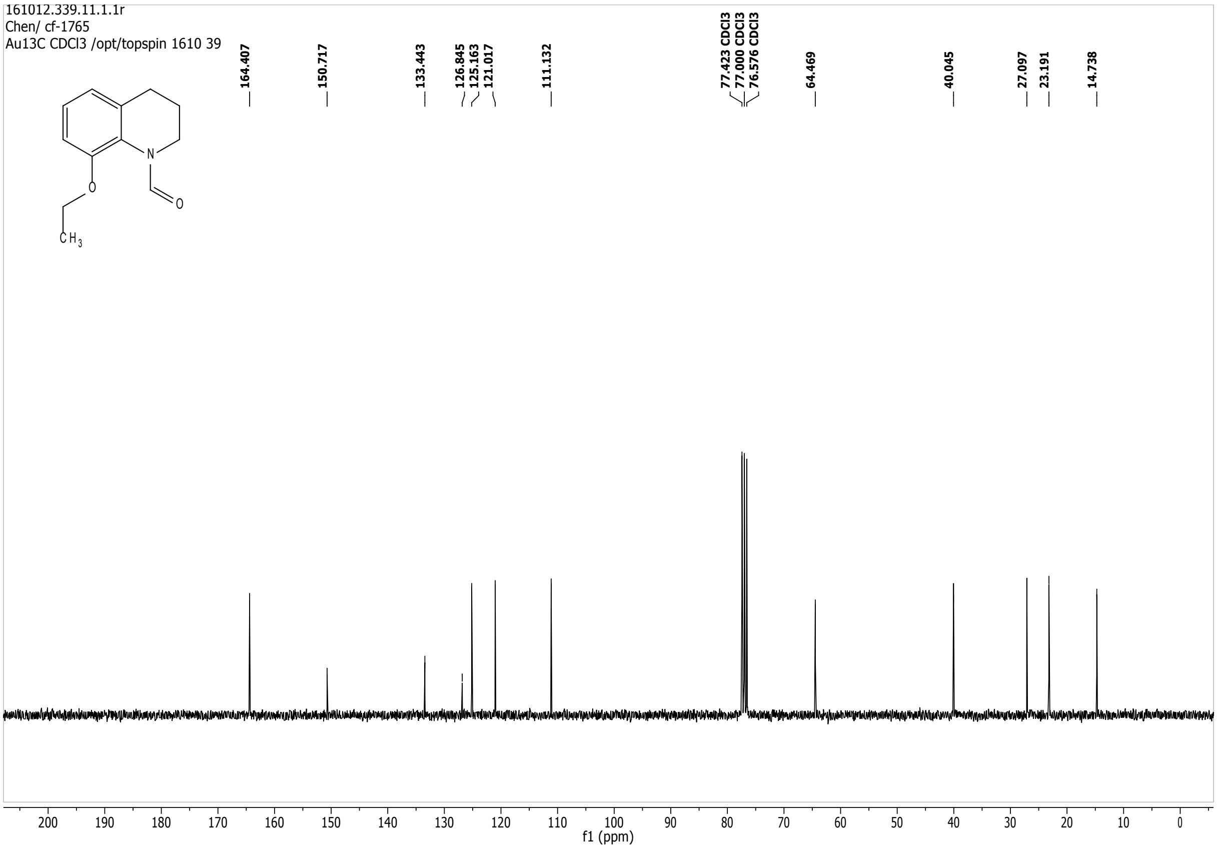
161012.339.11.1.1r
Chen/ cf-1765
Au13C CDCl3 /opt/topspin 1610 39
164.407
150.717
133.443
126.845
125.163
121.017
111.132
77.423 CDCl3
77.000 CDCl3
76.576 CDCl3
64.469
40.045
27.097
23.191
14.738
f1 (ppm)

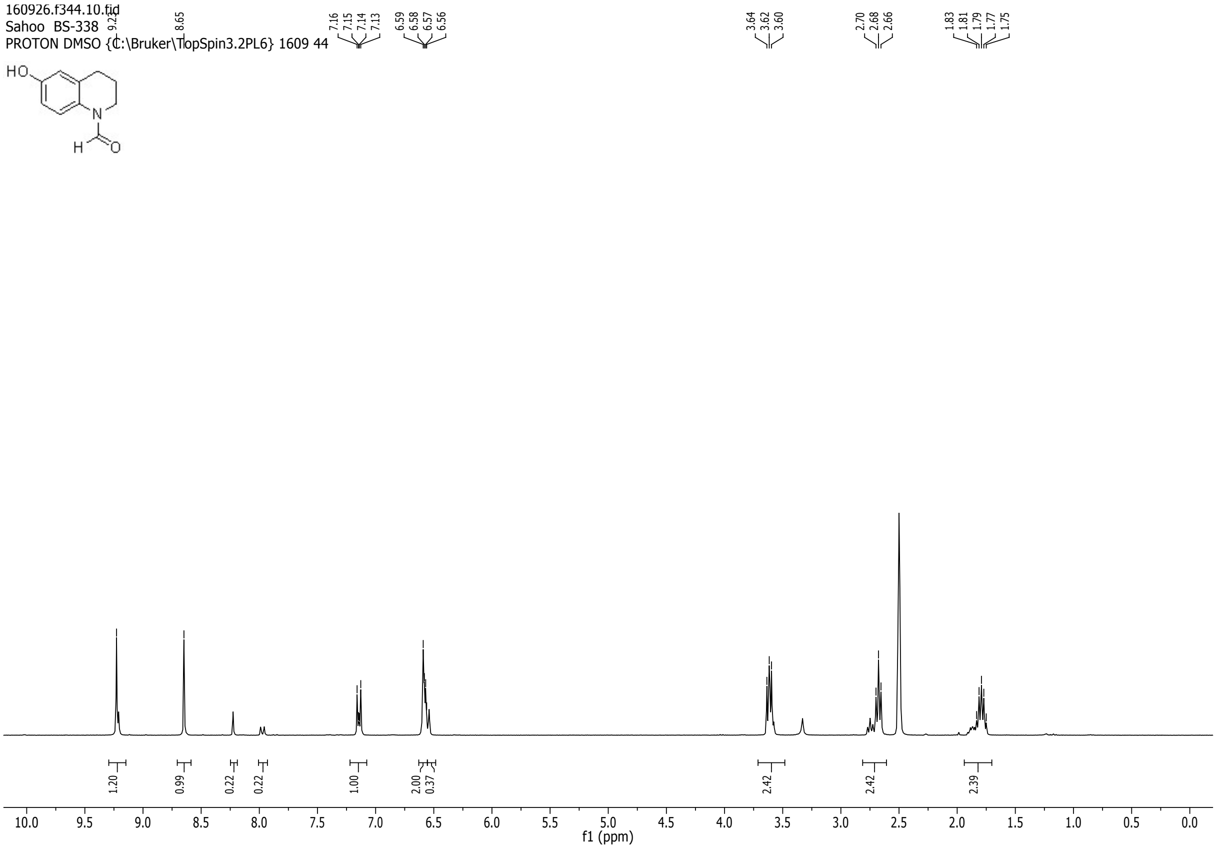
160926.f344.10.fid
Sahoo BS-338
PROTON DMSO {C:\Bruker\TopSpin3.2PL6} 1609 44
HO
N
H
O
8.65
7.16
7.15
7.14
7.13
6.59
6.58
6.57
6.56
3.64
3.62
3.60
2.70
2.68
2.66
1.83
1.81
1.79
1.77
1.75
1.20
0.99
0.22
0.22
1.00
2.00
0.37
2.42
2.42
2.39
10.0 9.5 9.0 8.5 8.0 7.5 7.0 6.5 6.0 5.5 5.0 4.5 4.0 3.5 3.0 2.5 2.0 1.5 1.0 0.5 0.0
f1 (ppm)

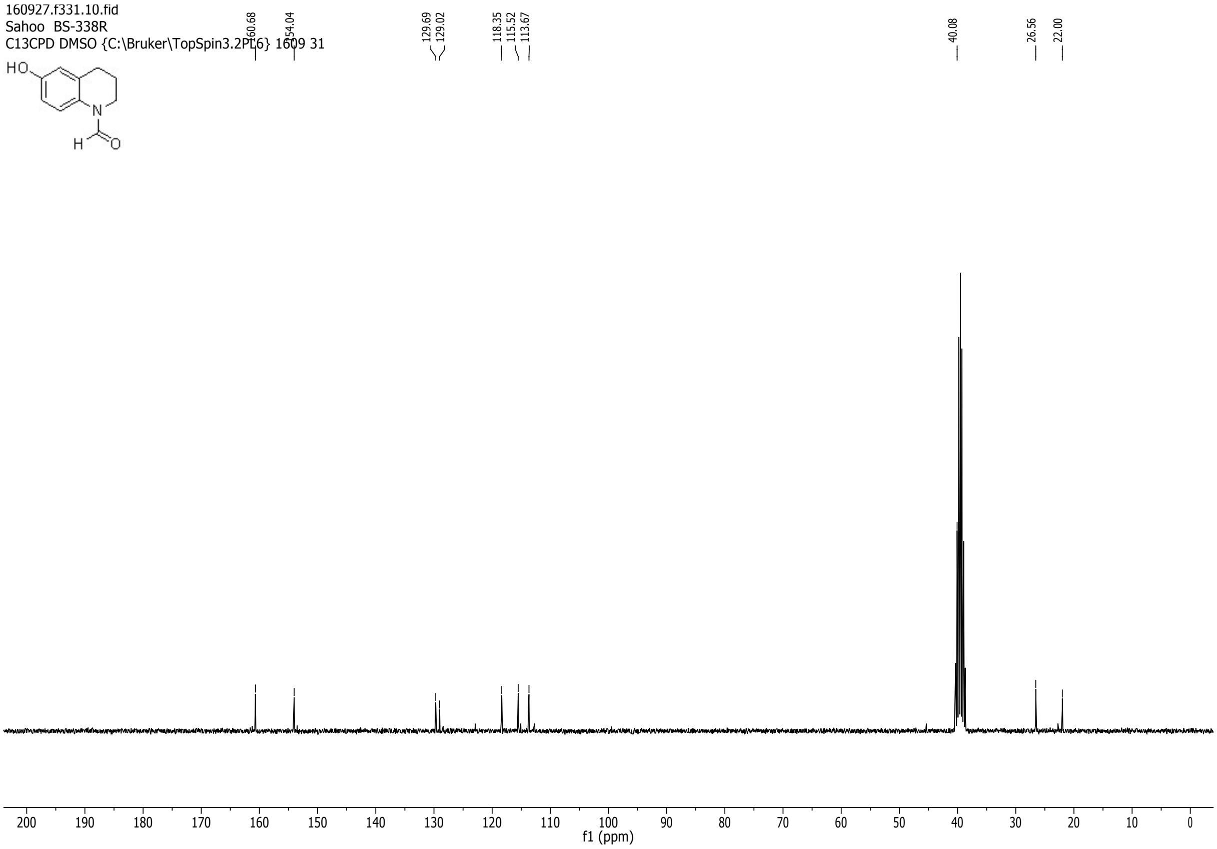
160927.f331.10.fid
Sahoo BS-338R
C13CPD DMSO {C:\Bruker\TopSpin3.2PL6} 1609 31
HO
N
H
O
154.04
129.69
129.02
118.35
115.52
113.67
40.08
26.56
22.00
200 190 180 170 160 150 140 130 120 110 100 90 80 70 60 50 40 30 20 10 0
f1 (ppm)

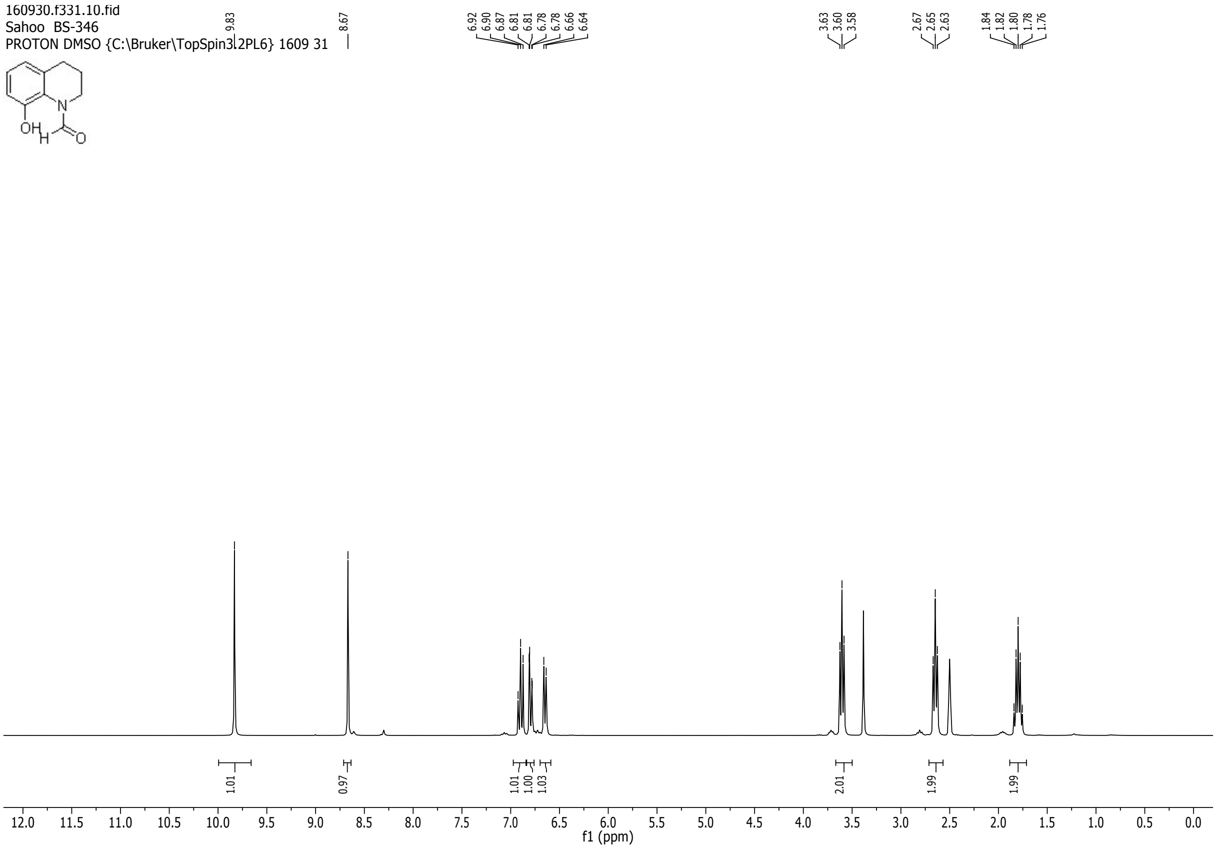
160930.f331.10.fid
Sahoo BS-346
PROTON DMSO {C:\Bruker\TopSpin3.2PL6} 1609 31
9.83
8.67
6.92 6.90 6.87 6.81 6.81 6.78 6.78 6.66 6.64
3.63 3.60 3.58
2.67 2.65 2.63
1.84 1.82 1.80 1.78 1.76
OH
H
N
O
1.01
0.97
1.01 1.00 1.03
2.01
1.99
1.99
f1 (ppm)

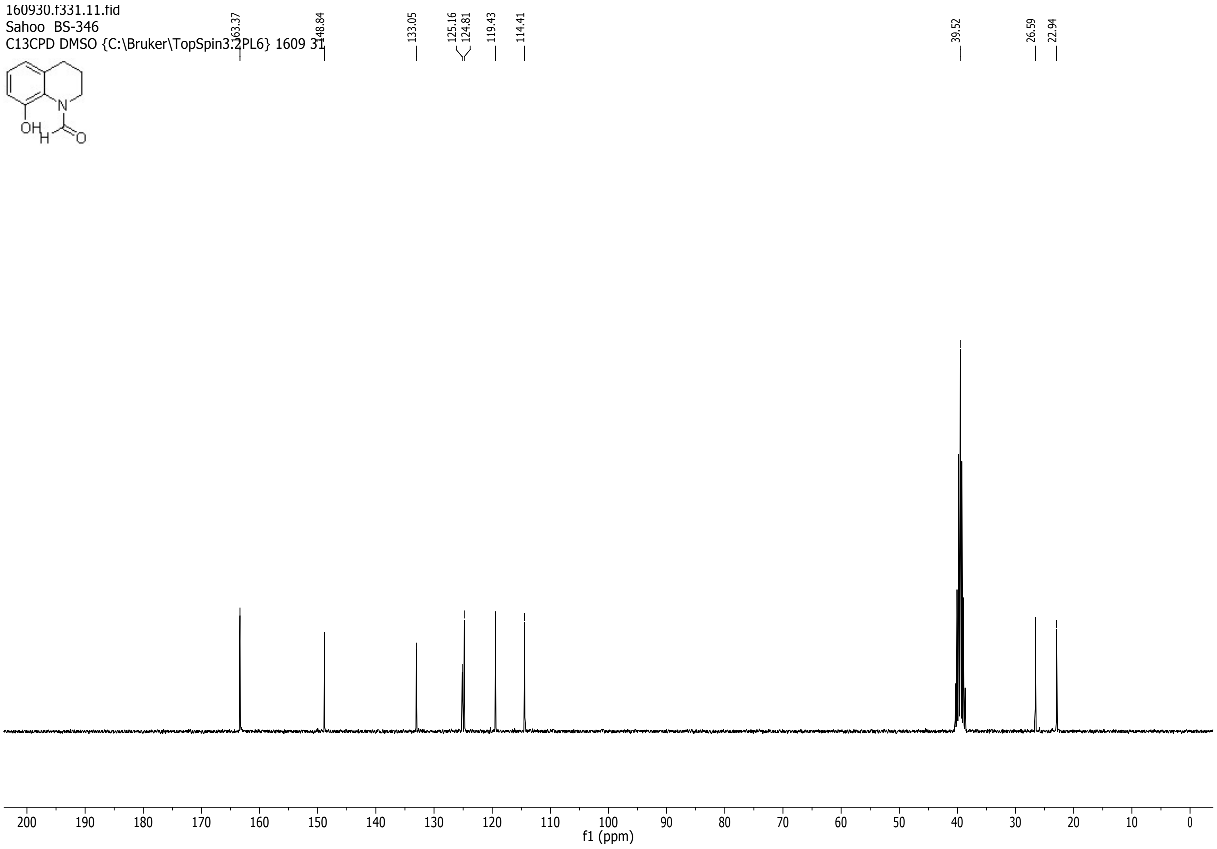
160930.f331.11.fid
Sahoo BS-346
C13CPD DMSO {C:\Bruker\TopSpin3.2PL6} 1609 31
163.37
148.84
133.05
125.16 124.81
119.43
114.41
39.52
26.59
22.94
OH
H
N
O
f1 (ppm)

160926.f343.10.fid
Sahoo BS-339
PROTON CDCl3 {C:\Bruker\TopSpin3.2PL6} 1609 43

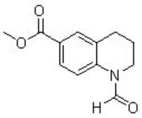

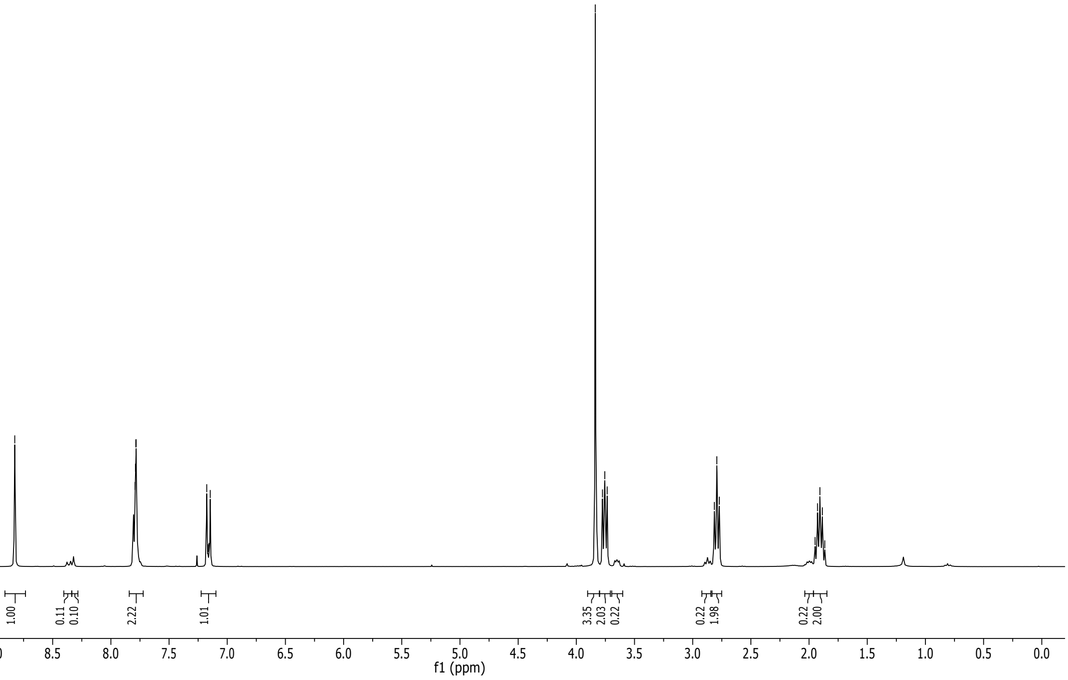

160926.f343.11.fid
Sahoo BS-339
C13CPD CDCl3 {C:\Bruker\TopSpin3.2PL6} 1609 43

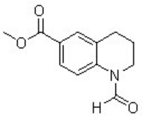

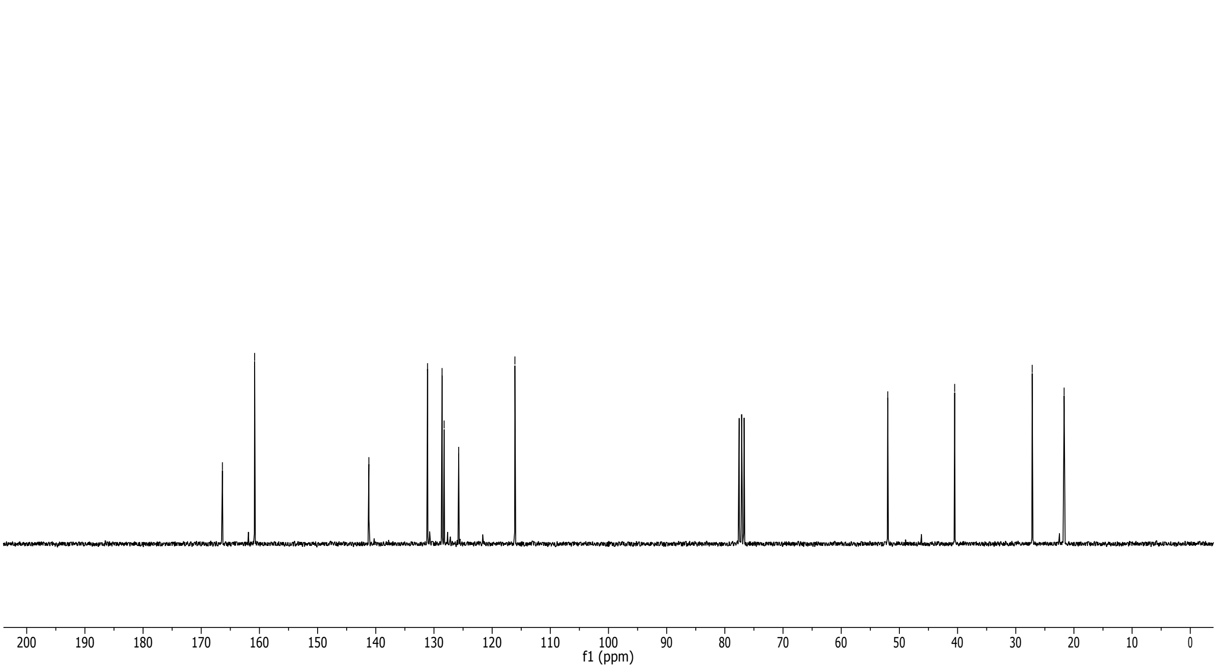

160919.308.10.fid
Sahoo BS-FC-334
Au1H CDCl3 /opt/topspin 1609 8

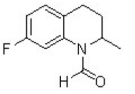

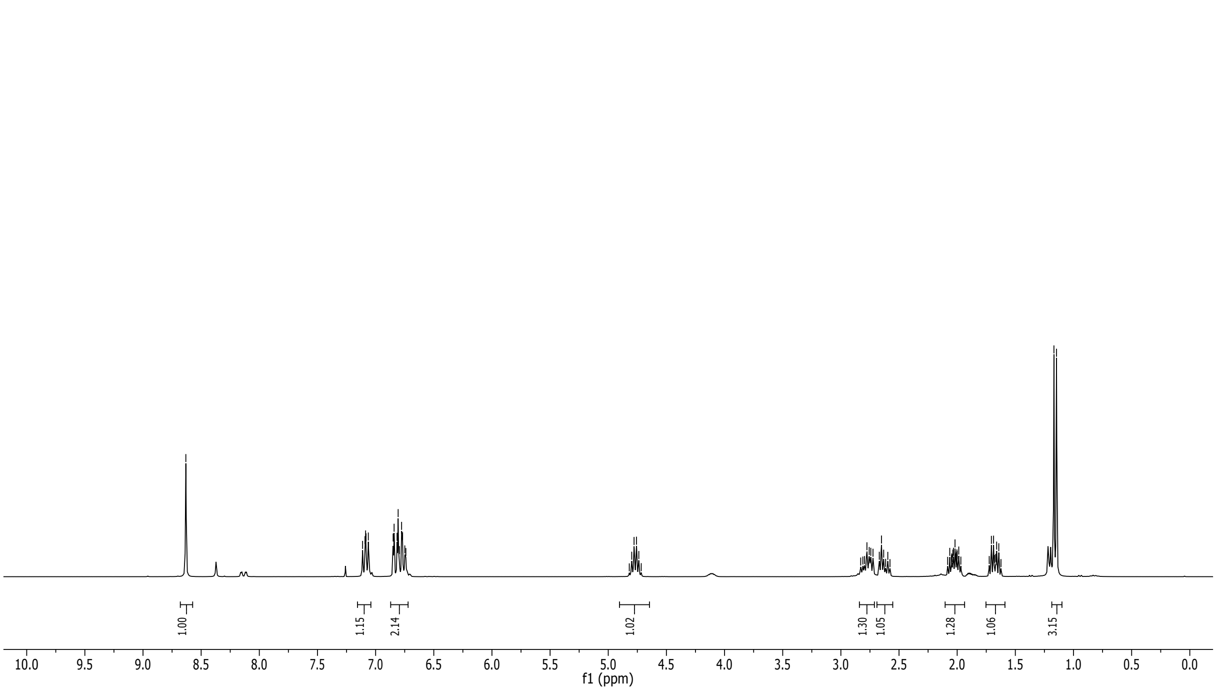

160919.308.11.fid
Sahoo BS-FC-334
Au13C CDCl3 /opt/topspin 1609 8

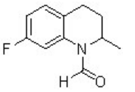

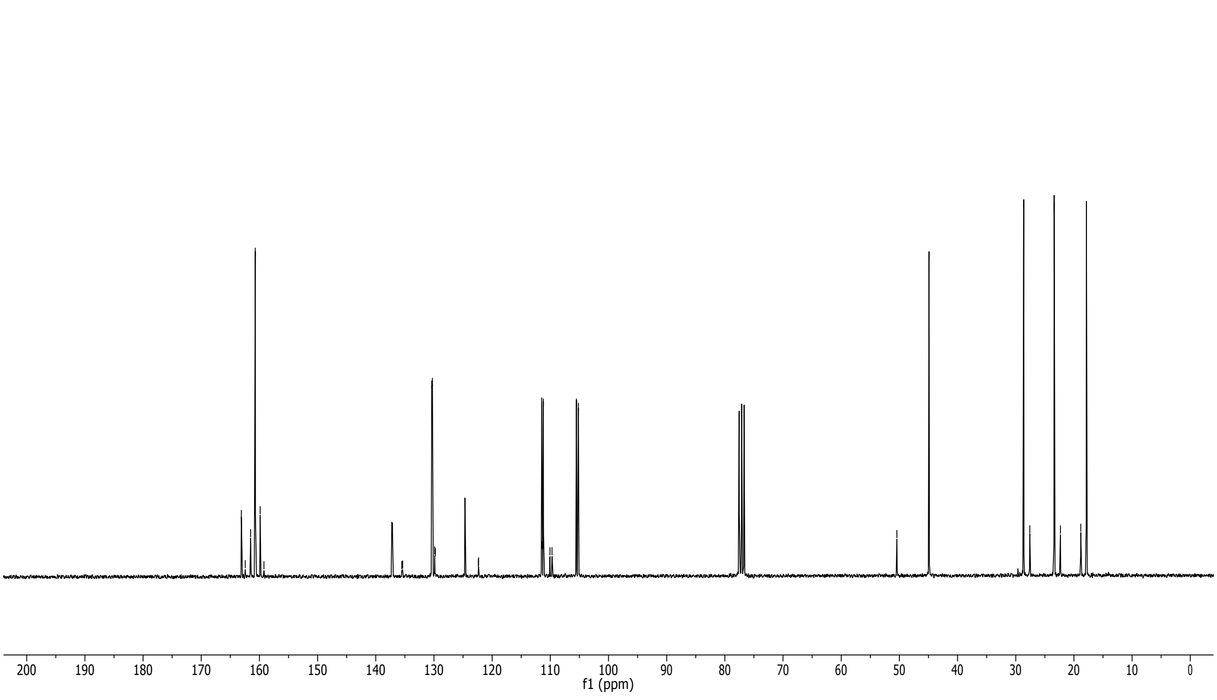

160927.322.10.fid
Sahoo, BS-334-R, 19F{1H}
Au19F CDCl3 /opt/topspin 1609 22

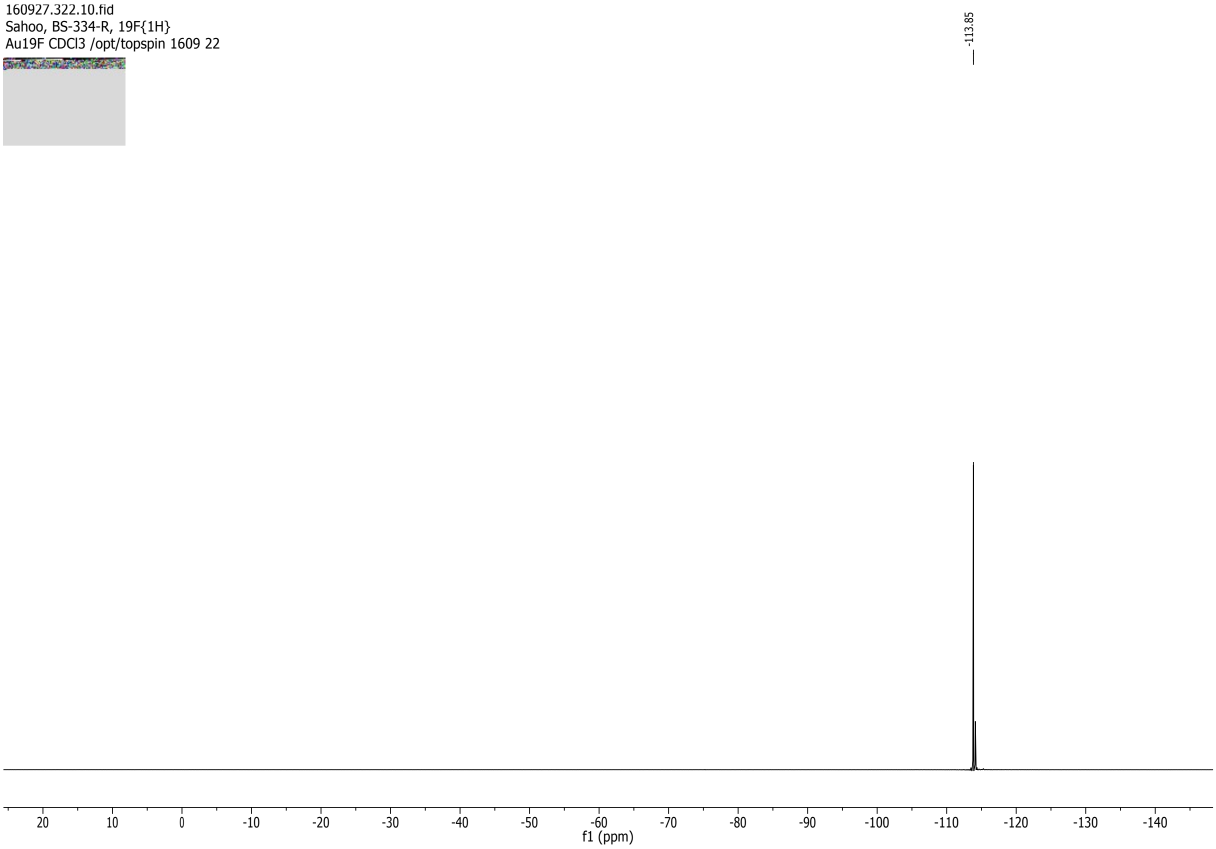

161028.430.10.1.1r
Feng Chen cf-1755
Au1H CDCl3 /opt/topspin 1610 30

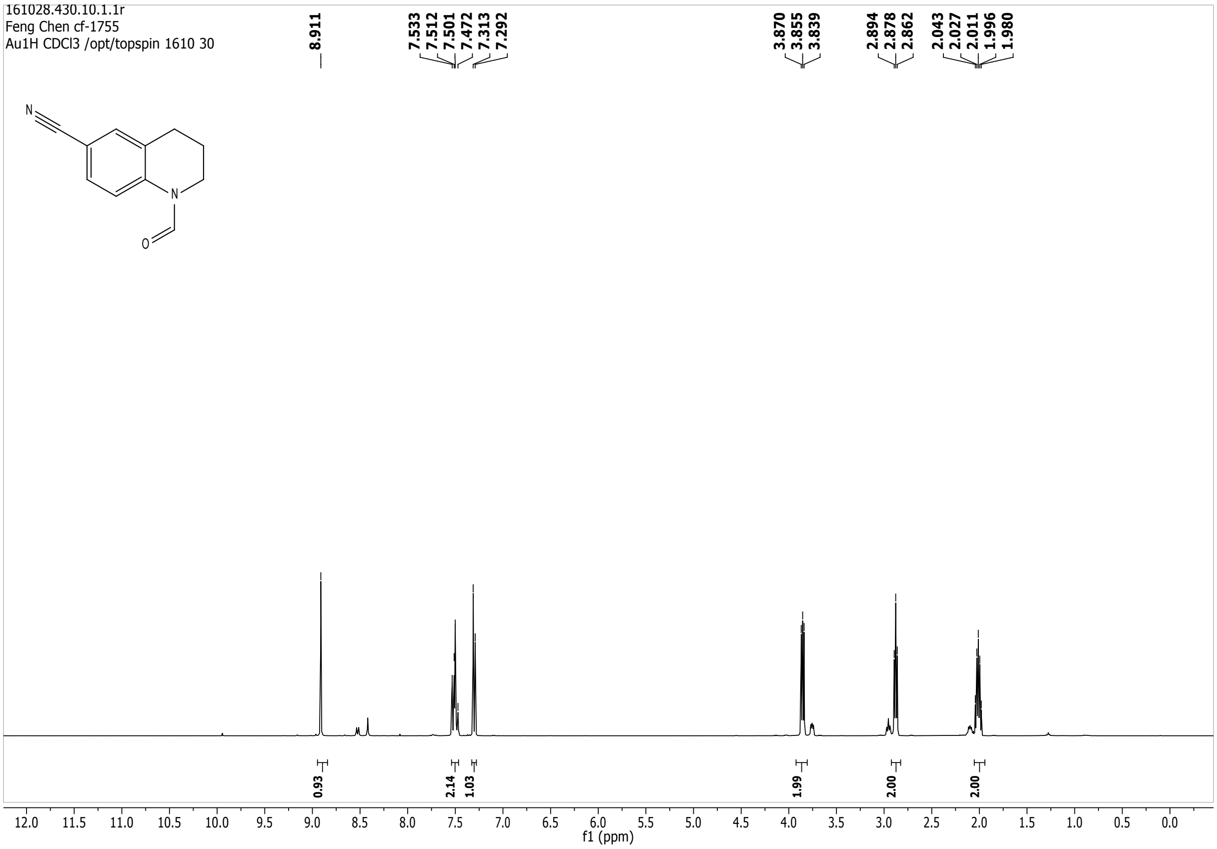

161028.430.11.1.1r
Feng Chen cf-1755
Au13C CDCl3 /opt/topspin 1610 30

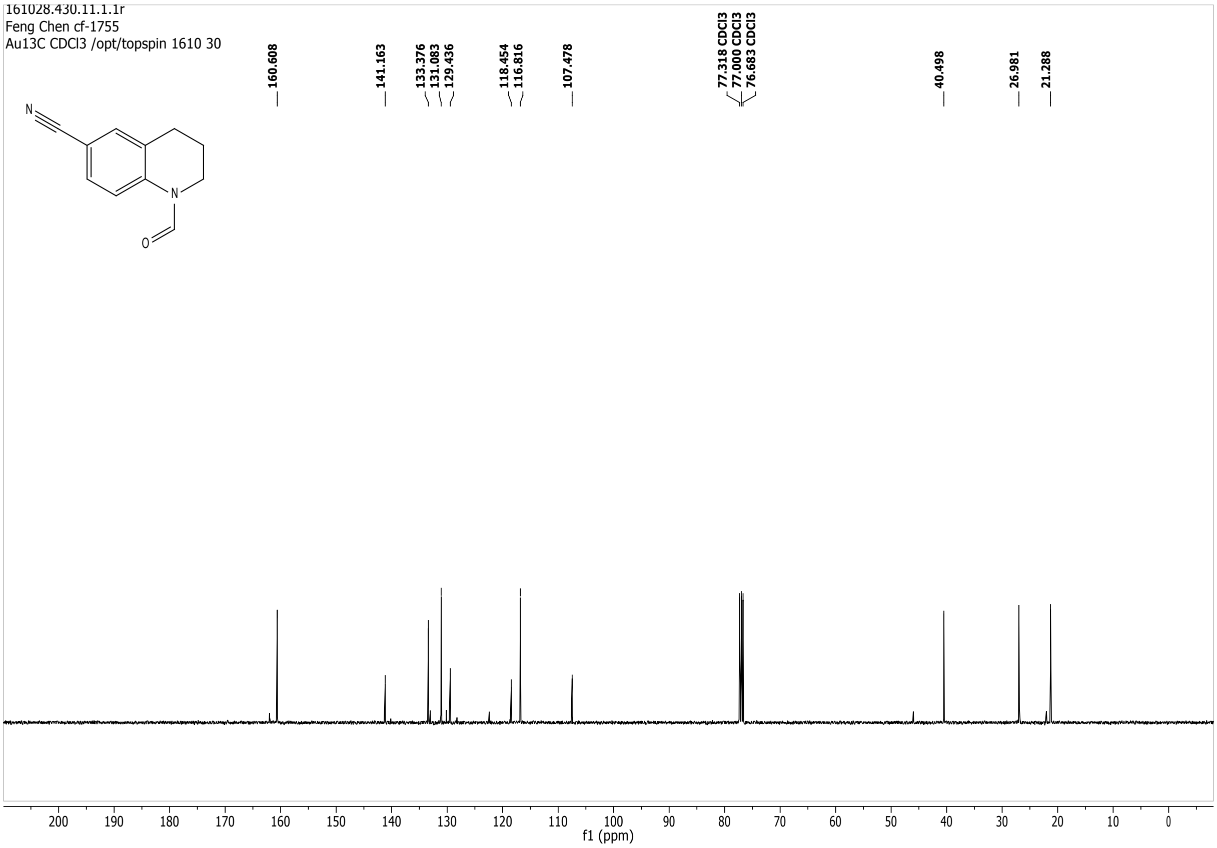

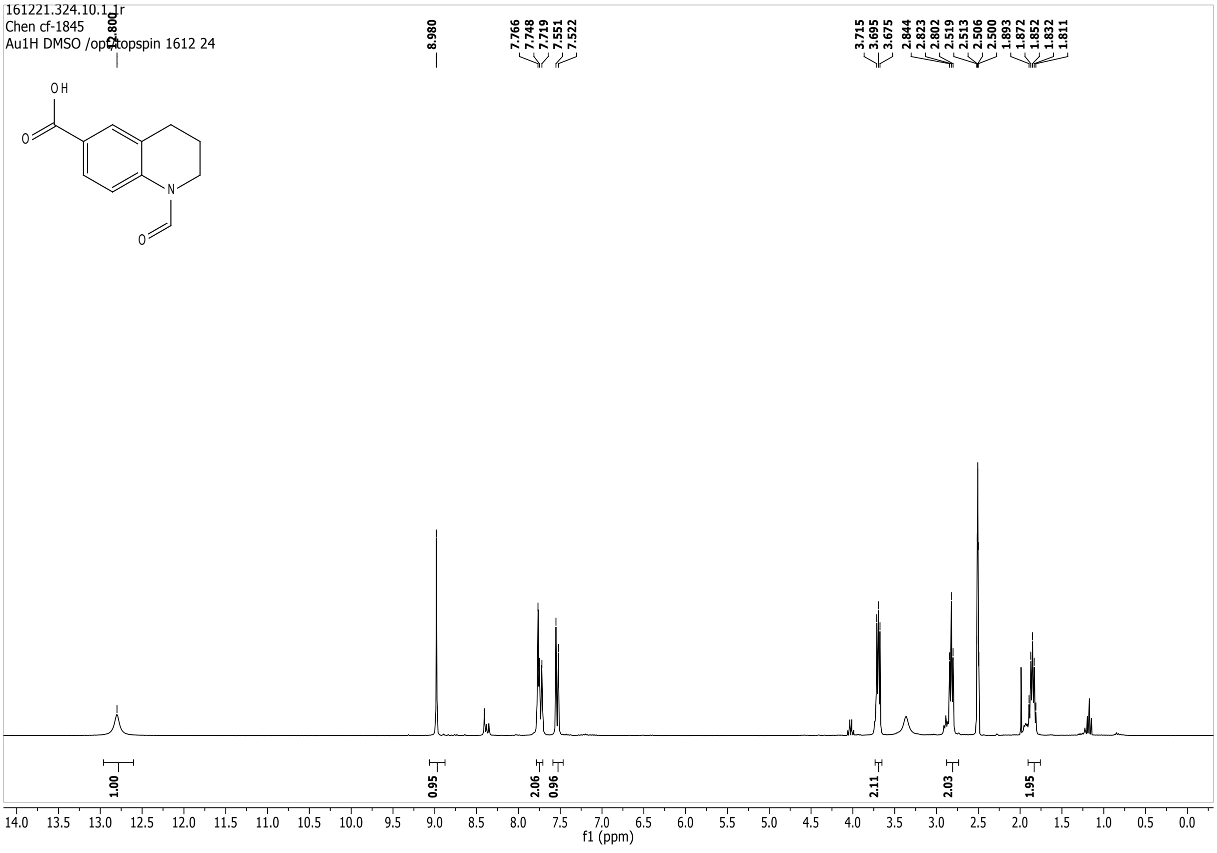
161221.324.10.1.1r
Chen cf-1845
Au1H DMSO /opt/topspin 1612 24
12.800
8.980
7.766
7.748
7.719
7.551
7.522
3.715
3.695
3.675
2.844
2.823
2.802
2.519
2.513
2.506
2.500
1.893
1.872
1.852
1.832
1.811
OH
O
N
O
1.00
0.95
2.06
0.96
2.11
2.03
1.95
14.0 13.5 13.0 12.5 12.0 11.5 11.0 10.5 10.0 9.5 9.0 8.5 8.0 7.5 7.0 6.5 6.0 5.5 5.0 4.5 4.0 3.5 3.0 2.5 2.0 1.5 1.0 0.5 0.0
f1 (ppm)

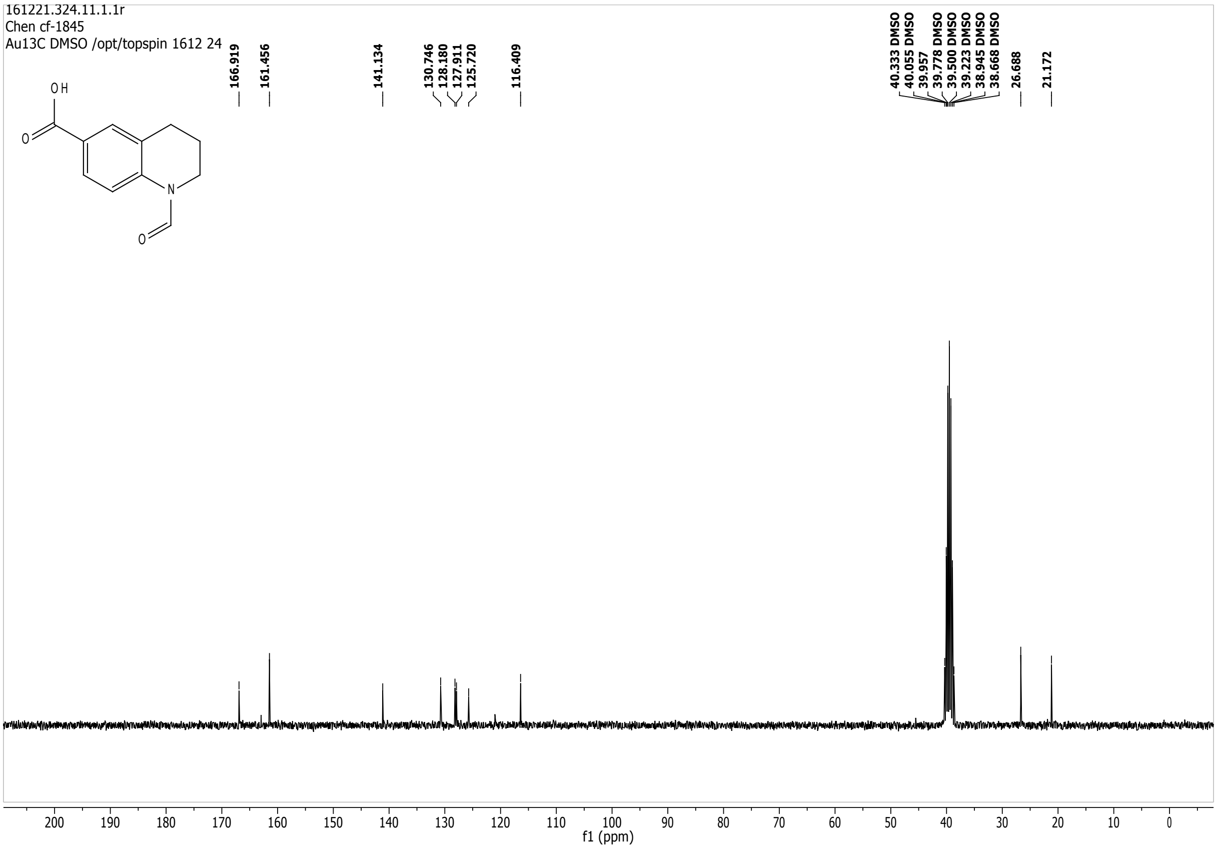
161221.324.11.1.1r
Chen cf-1845
Au13C DMSO /opt/topspin 1612 24
166.919
161.456
141.134
130.746
128.180
127.911
125.720
116.409
40.333 DMSO
40.055 DMSO
39.957
39.778 DMSO
39.500 DMSO
39.223 DMSO
38.945 DMSO
38.668 DMSO
26.688
21.172
OH
O
N
O
200 190 180 170 160 150 140 130 120 110 100 90 80 70 60 50 40 30 20 10 0
f1 (ppm)

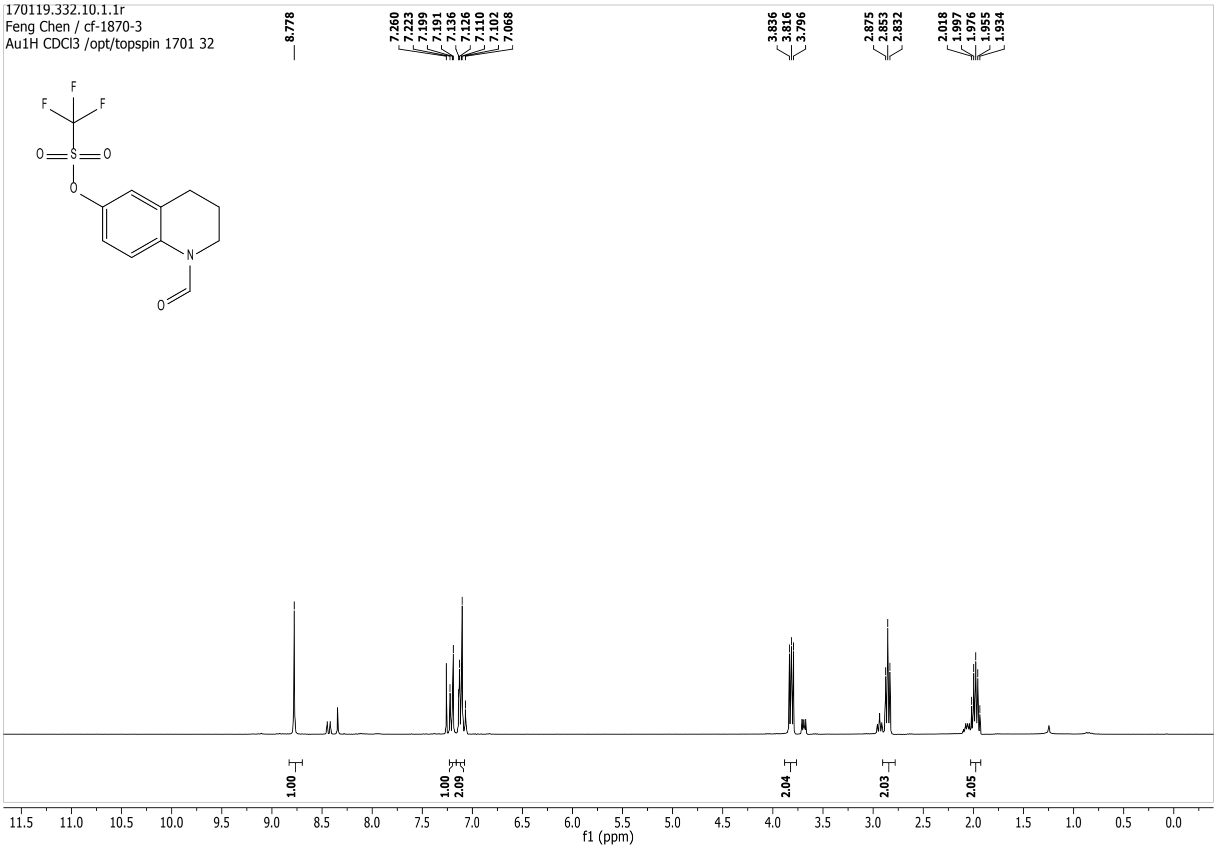
170119.332.10.1.1r
Feng Chen / cf-1870-3
Au1H CDCl3 /opt/topspin 1701 32
8.778
7.260
7.223
7.199
7.191
7.136
7.126
7.110
7.102
7.068
3.836
3.816
3.796
2.875
2.853
2.832
2.018
1.997
1.976
1.955
1.934
1.00
1.00
2.09
2.04
2.03
2.05
f1 (ppm)

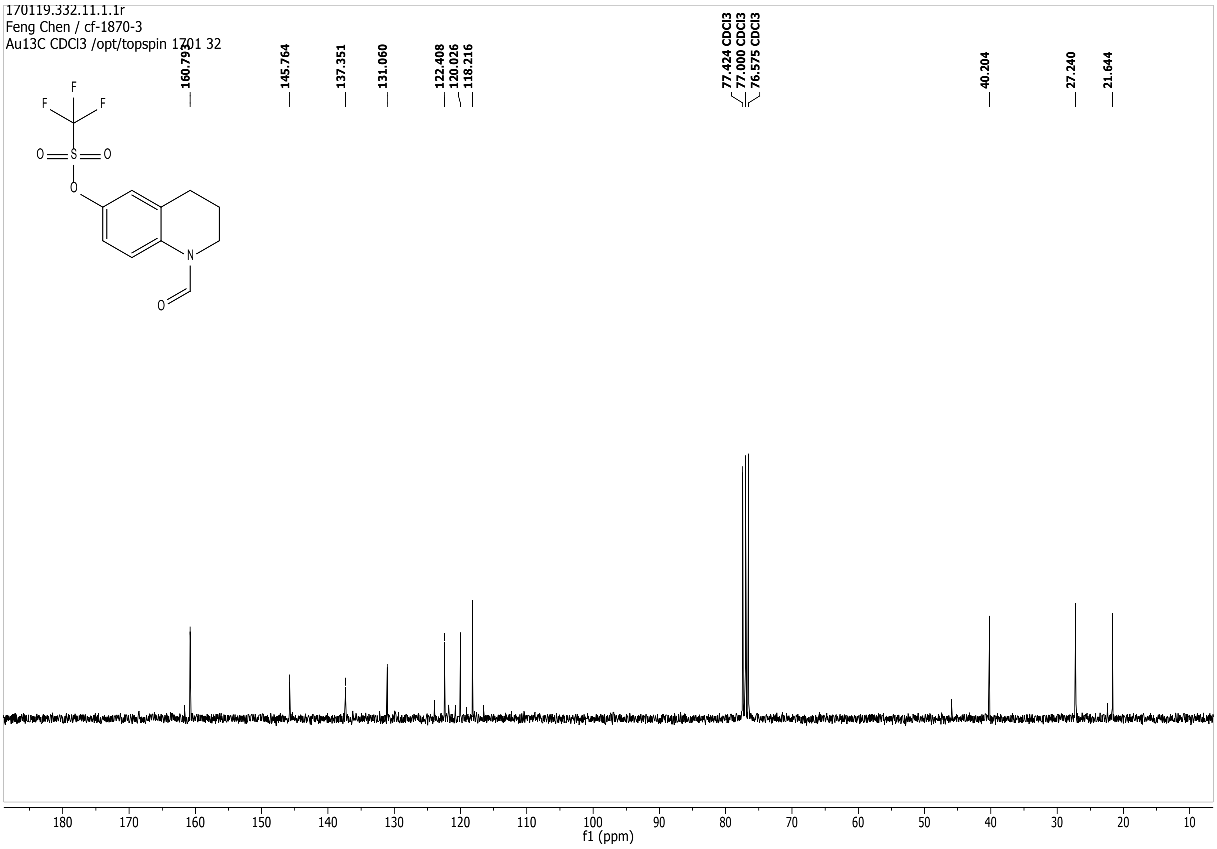
170119.332.11.1.1r
Feng Chen / cf-1870-3
Au13C CDCl3 /opt/topspin 1701 32
160.792
145.764
137.351
131.060
122.408
120.026
118.216
77.424 CDCl3
77.000 CDCl3
76.575 CDCl3
40.204
27.240
21.644
f1 (ppm)

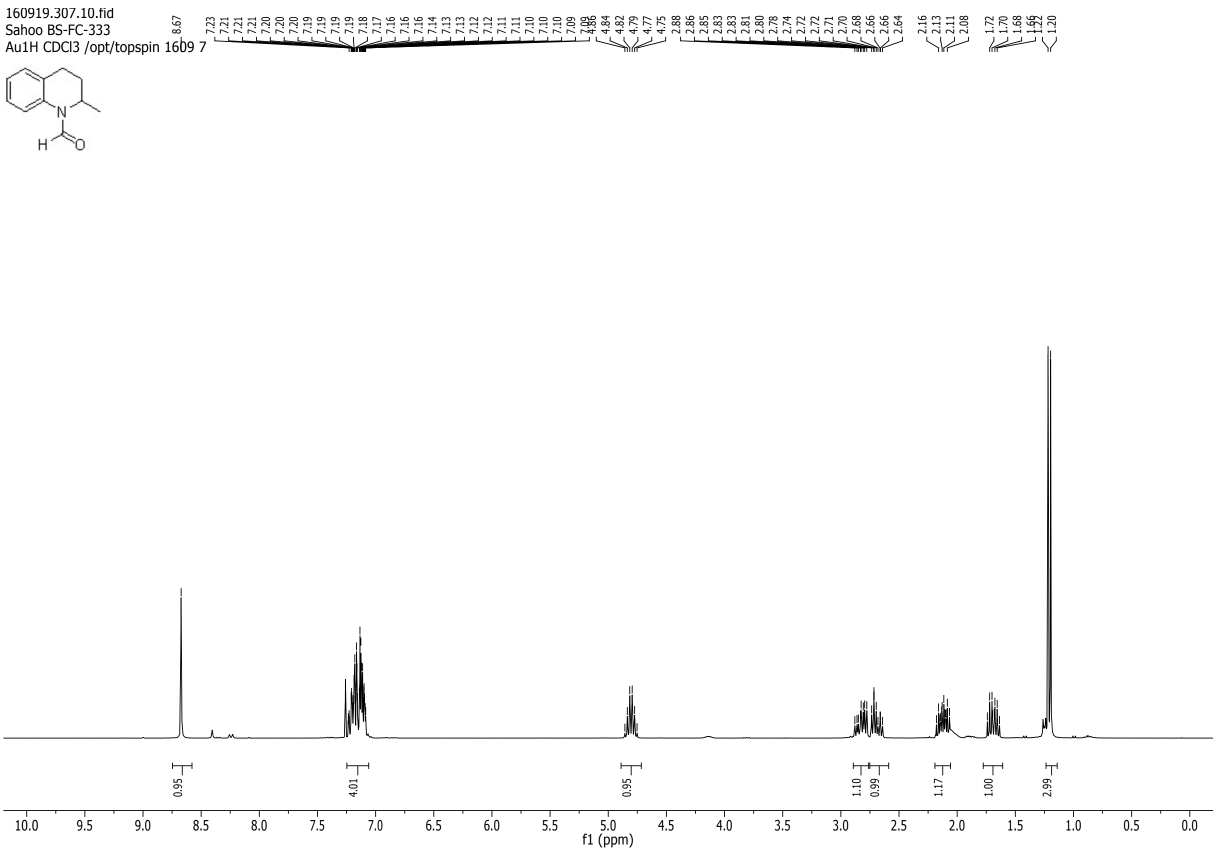
160919.307.10.fid
Sahoo BS-FC-333
Au1H CDCl3 /opt/topspin 1609 7
0.95
4.01
0.95
1.10
0.99
1.17
1.00
2.99
f1 (ppm)

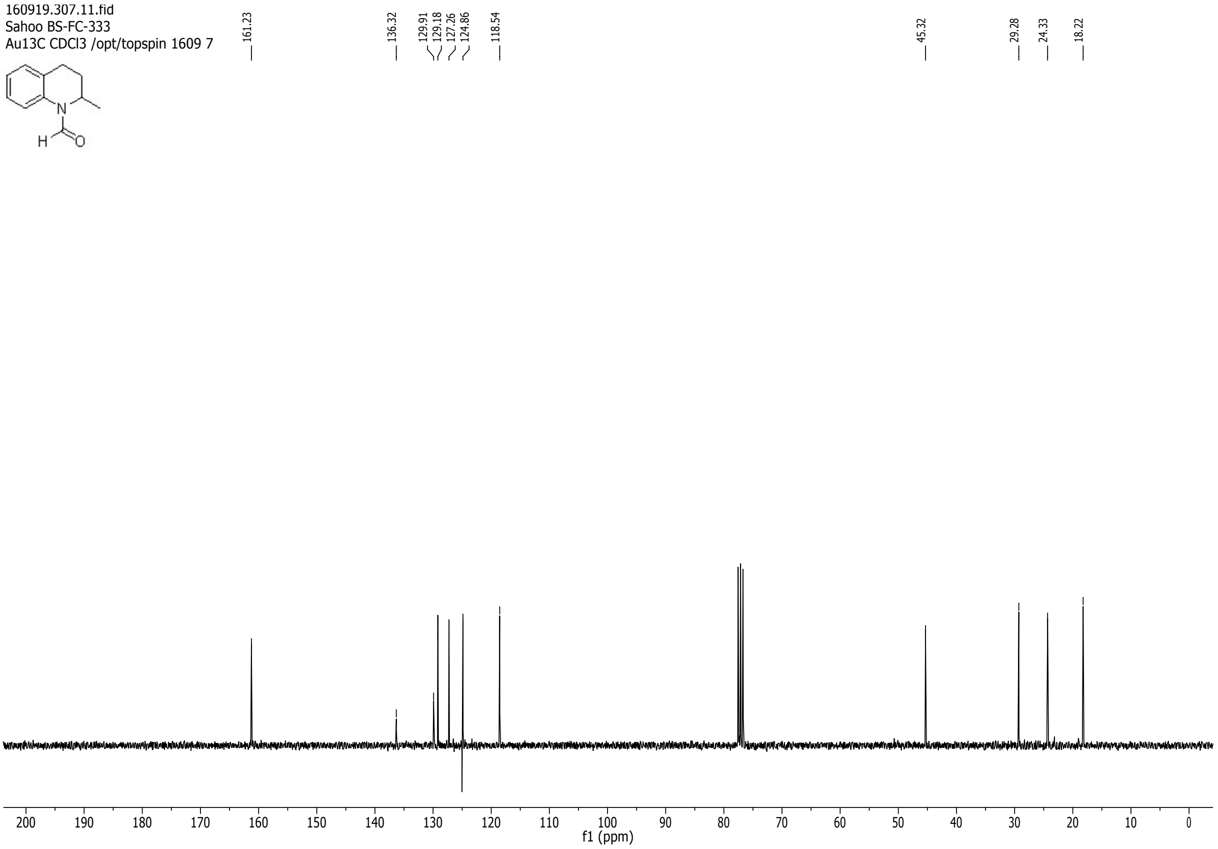
160919.307.11.fid
Sahoo BS-FC-333
Au13C CDCl3 /opt/topspin 1609 7
161.23
136.32
129.91
129.18
127.26
124.86
118.54
45.32
29.28
24.33
18.22
f1 (ppm)

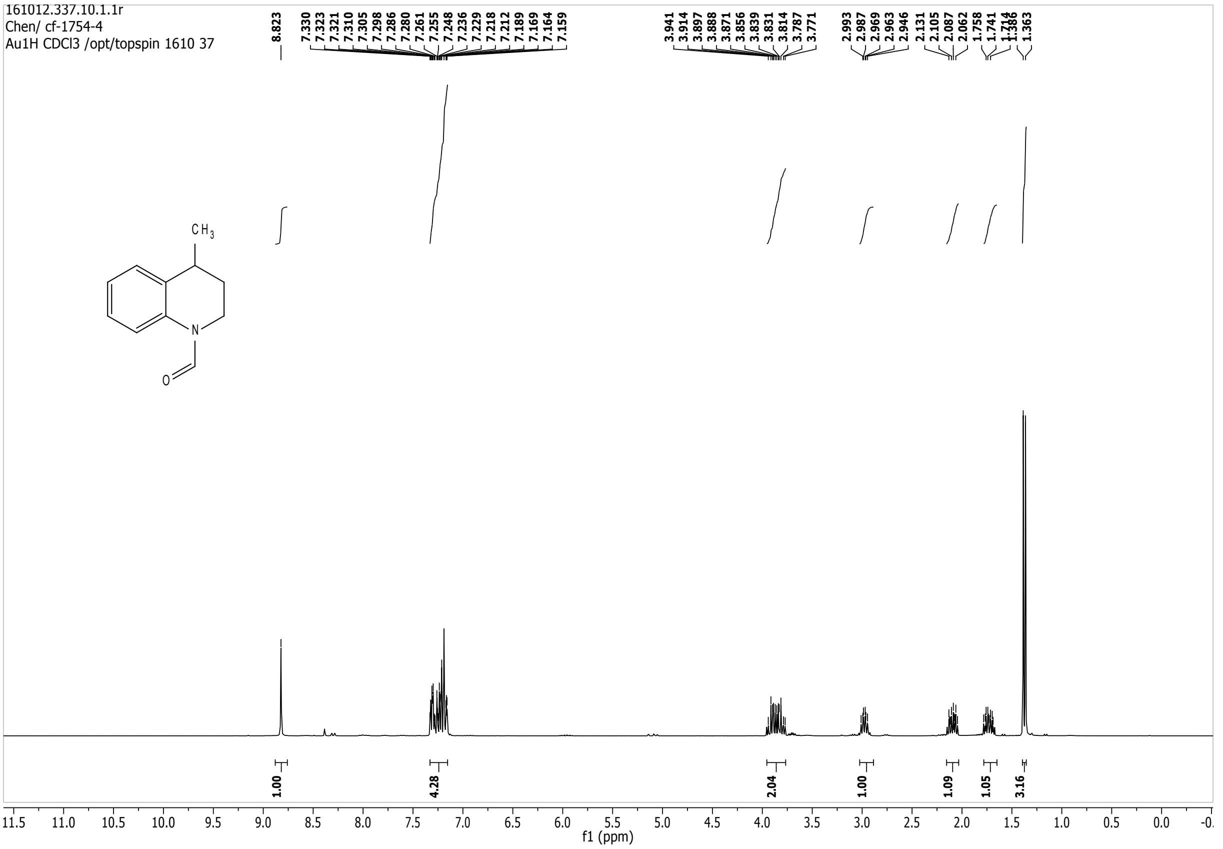
161012.337.10.1.1r
Chen/ cf-1754-4
Au1H CDCl3 /opt/topspin 1610 37
8.823
7.330 7.323 7.321 7.310 7.305 7.298 7.286 7.280 7.261 7.255 7.248 7.236 7.229 7.218 7.212 7.189 7.169 7.164 7.159
3.941 3.914 3.897 3.888 3.871 3.856 3.839 3.831 3.814 3.787 3.771
2.993 2.987 2.969 2.963 2.946 2.131 2.105 2.087 2.062 1.758 1.741 1.363
CH3
N
O
1.00
4.28
2.04
1.00
1.09
1.05
3.16
f1 (ppm)

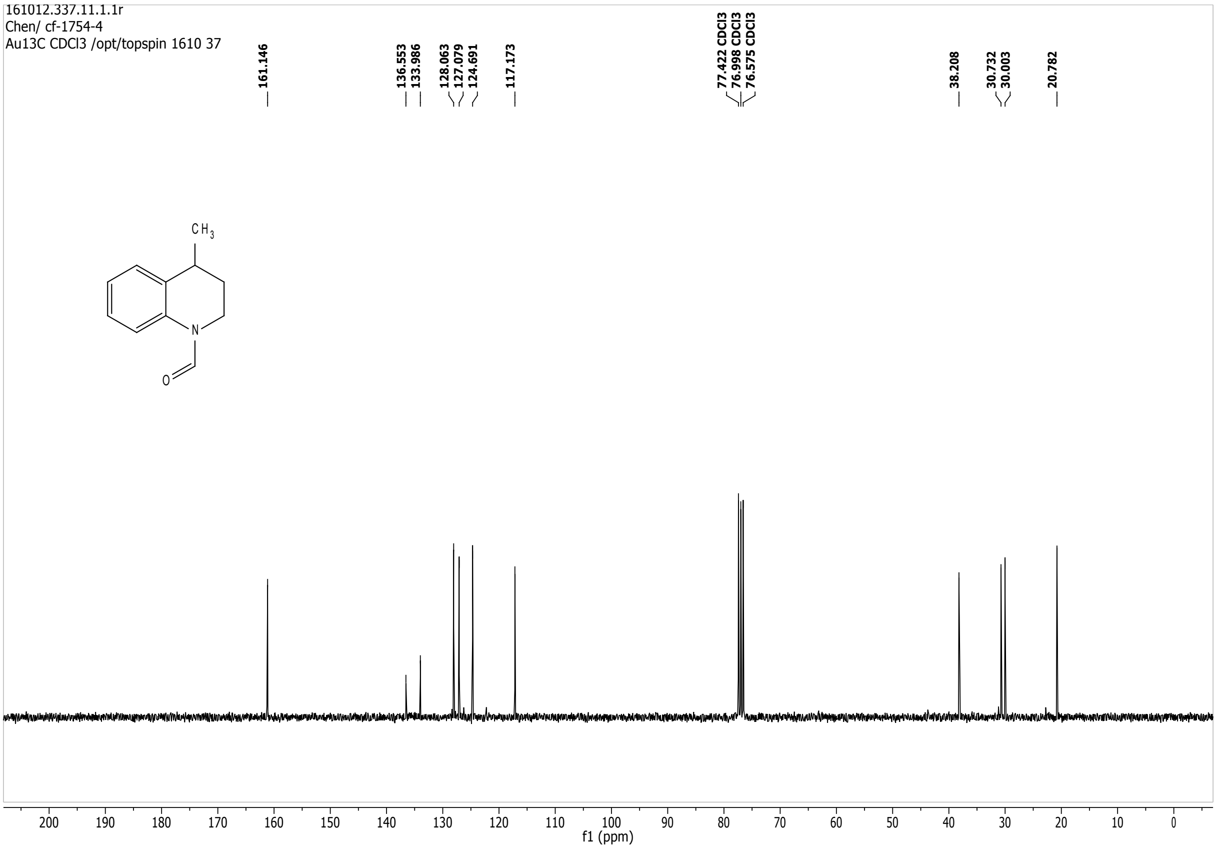
161012.337.11.1.1r
Chen/ cf-1754-4
Au13C CDCl3 /opt/topspin 1610 37
161.146
136.553
133.986
128.063
127.079
124.691
117.173
77.422 CDCl3
76.998 CDCl3
76.575 CDCl3
38.208
30.732
30.003
20.782
CH3
N
O
f1 (ppm)

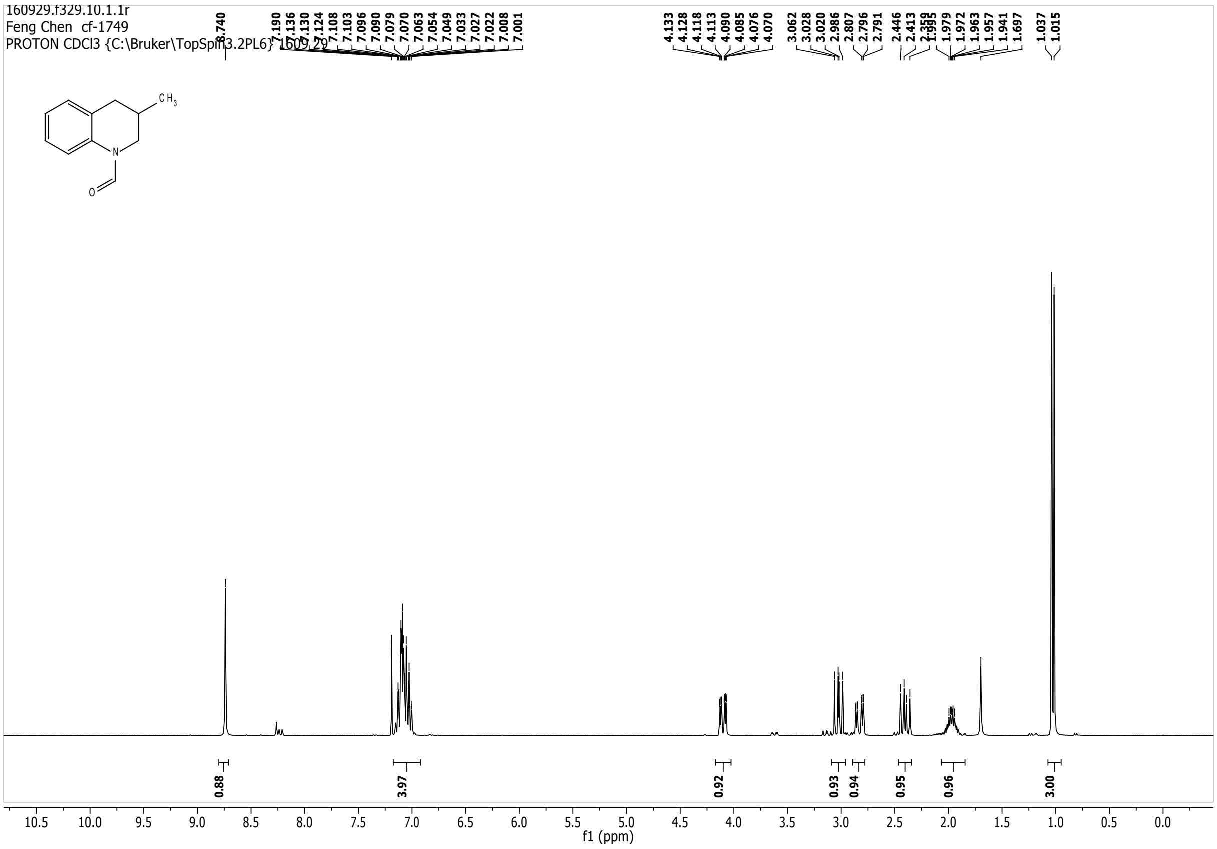
160929.f329.10.1.1r
Feng Chen cf-1749
PROTON CDCl3 {C:\Bruker\TopSpin3.2PL6} 1609 29
CH3
N
O
8.740
7.190 7.136 7.130 7.124 7.108 7.103 7.096 7.090 7.079 7.070 7.063 7.054 7.049 7.033 7.027 7.022 7.008 7.001
4.133 4.128 4.118 4.113 4.090 4.085 4.076 4.070
3.062 3.028 3.020 2.986 2.807 2.796 2.791
2.446 2.413
1.979 1.972 1.963 1.957 1.941 1.697
1.037 1.015
0.88 3.97 0.92 0.93 0.94 0.95 0.96 3.00
10.5 10.0 9.5 9.0 8.5 8.0 7.5 7.0 6.5 6.0 5.5 5.0 4.5 4.0 3.5 3.0 2.5 2.0 1.5 1.0 0.5 0.0
f1 (ppm)

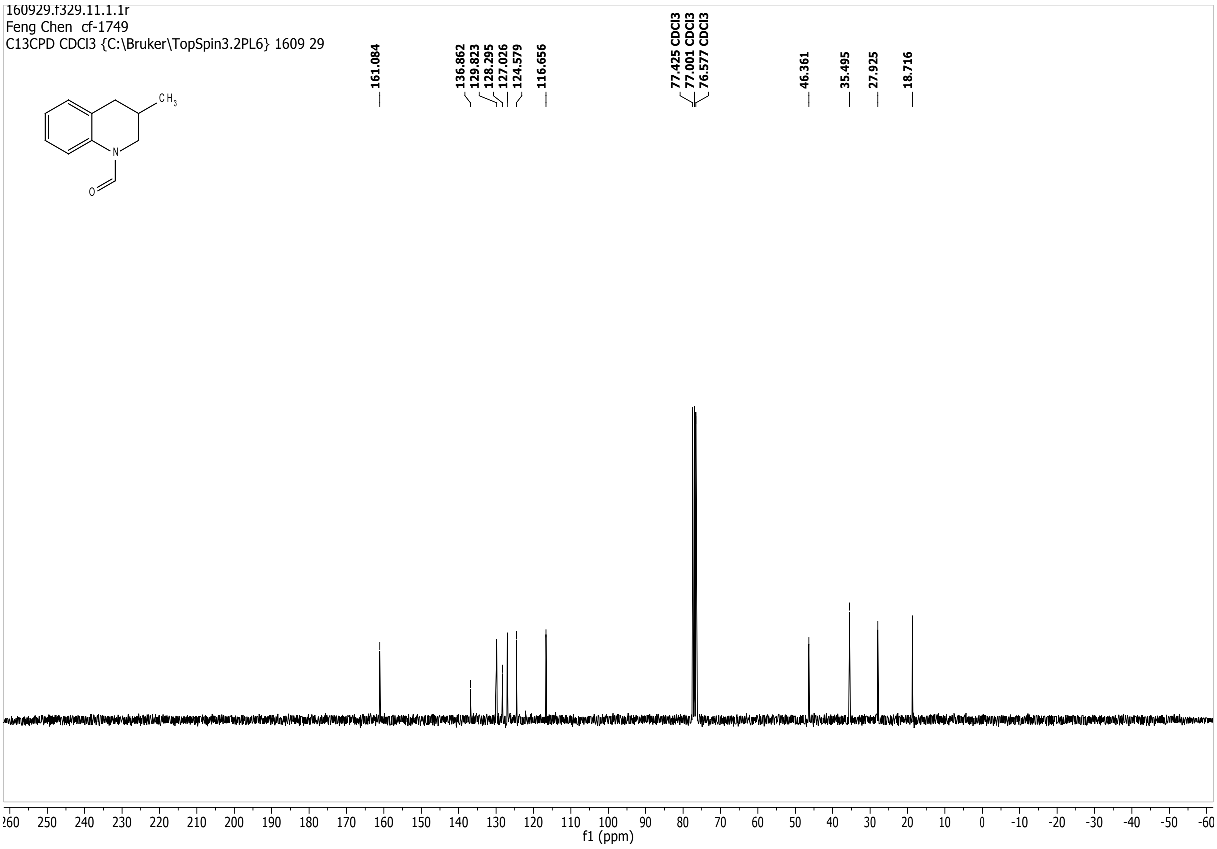
160929.f329.11.1.1r
Feng Chen cf-1749
C13CPD CDCl3 {C:\Bruker\TopSpin3.2PL6} 1609 29
CH3
N
O
161.084
136.862 129.823 128.295 127.026 124.579
116.656
77.425 CDCl3
77.001 CDCl3
76.577 CDCl3
46.361
35.495
27.925
18.716
260 250 240 230 220 210 200 190 180 170 160 150 140 130 120 110 100 90 80 70 60 50 40 30 20 10 0 -10 -20 -30 -40 -50 -60
f1 (ppm)

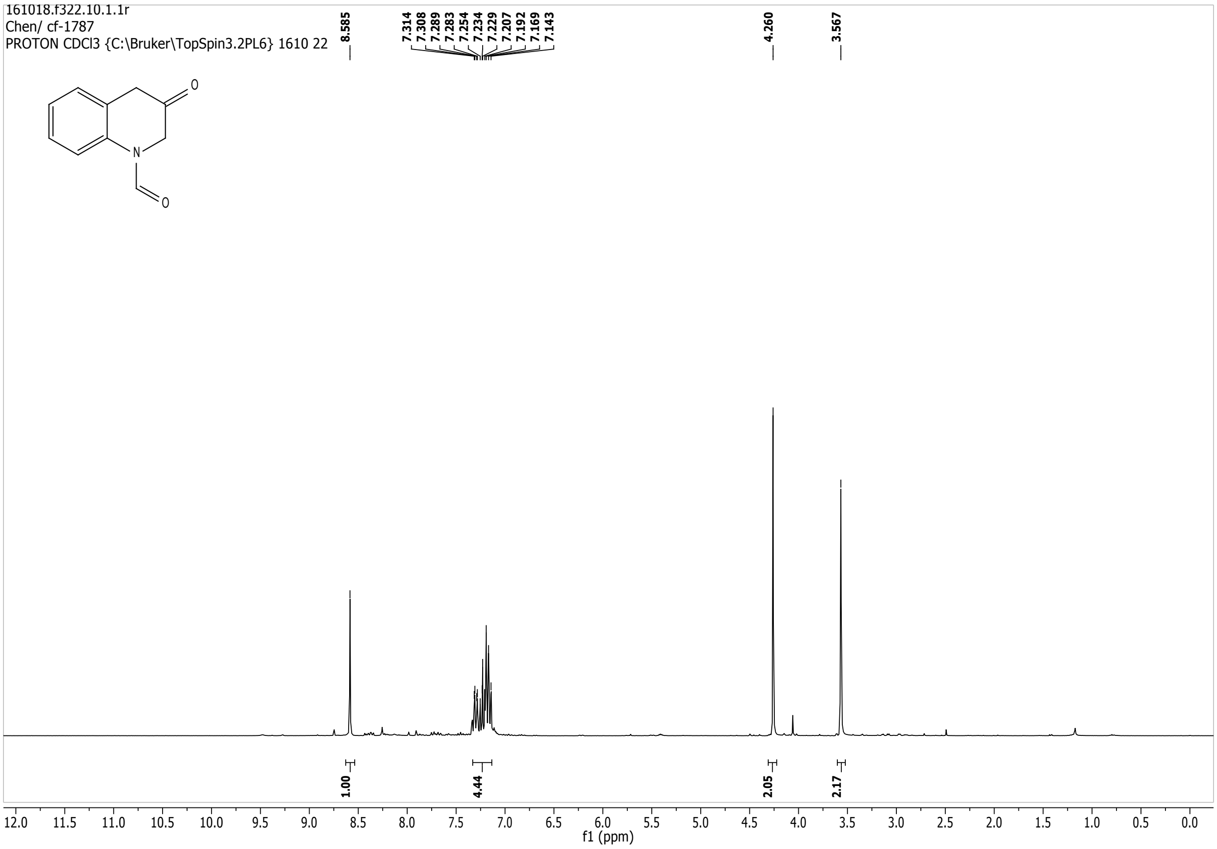
161018.f322.10.1.1r
Chen/ cf-1787
PROTON CDCl3 {C:\Bruker\TopSpin3.2PL6} 1610 22
8.585
7.314
7.308
7.289
7.283
7.254
7.234
7.229
7.207
7.192
7.169
7.143
4.260
3.567
O
N
O
1.00
4.44
2.05
2.17
12.0 11.5 11.0 10.5 10.0 9.5 9.0 8.5 8.0 7.5 7.0 6.5 6.0 5.5 5.0 4.5 4.0 3.5 3.0 2.5 2.0 1.5 1.0 0.5 0.0
f1 (ppm)

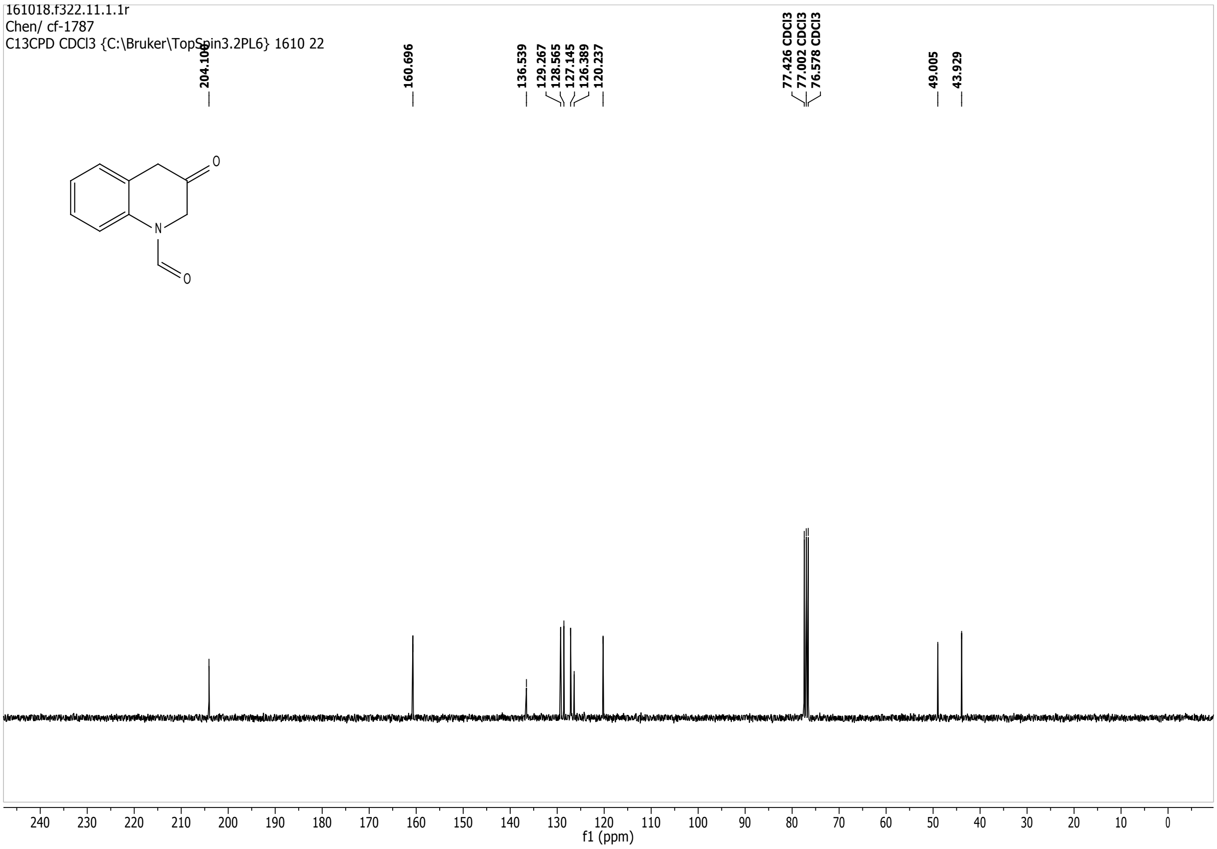
161018.f322.11.1.1r
Chen/ cf-1787
C13CPD CDCl3 {C:\Bruker\TopSpin3.2PL6} 1610 22
204.100
160.696
136.539
129.267
128.565
127.145
126.389
120.237
77.426 CDCl3
77.002 CDCl3
76.578 CDCl3
49.005
43.929
O
N
O
240 230 220 210 200 190 180 170 160 150 140 130 120 110 100 90 80 70 60 50 40 30 20 10 0
f1 (ppm)

161028.431.10.1.1r
Feng Chen cf-1759
Au1H CDCl3 /opt/topspin 1610 31

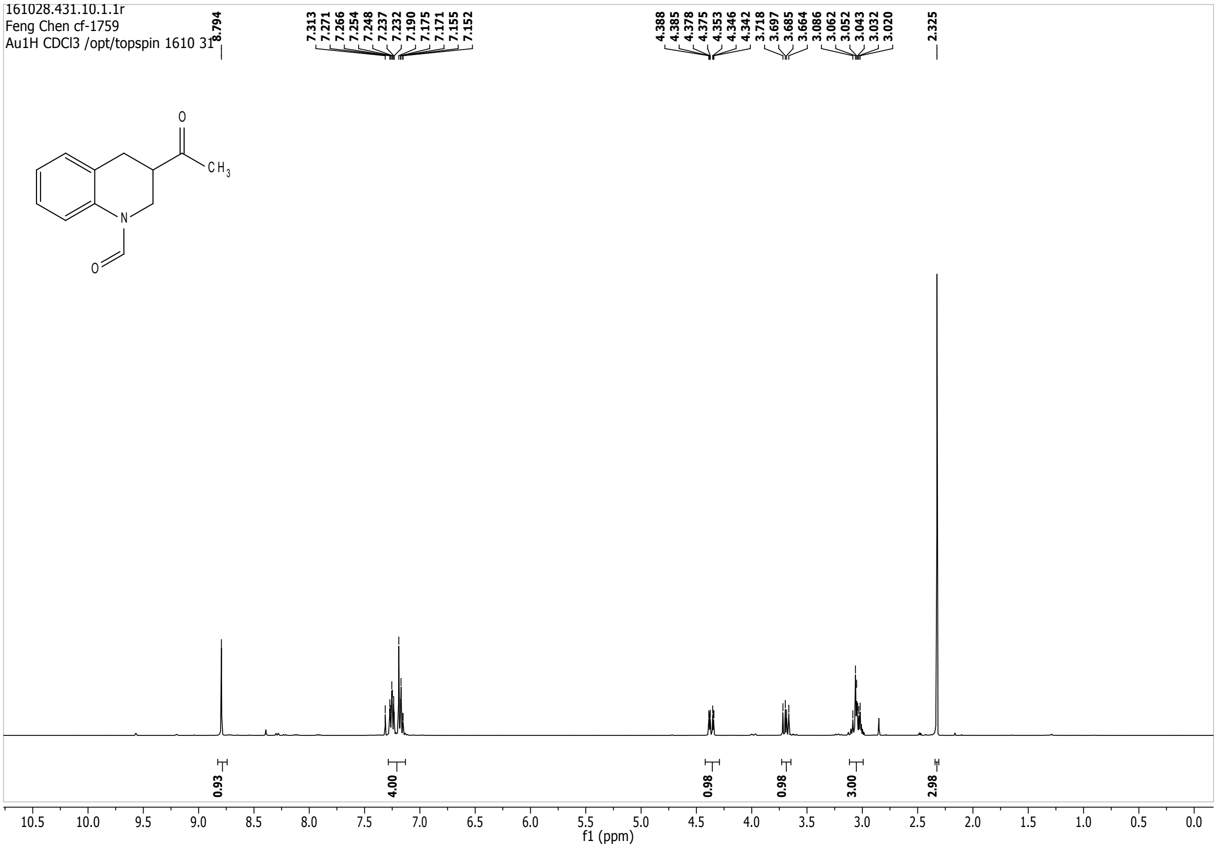

161028.431.11.1.1r
Feng Chen cf-1759
Au13C CDCl3 /opt/topspin 1610 31

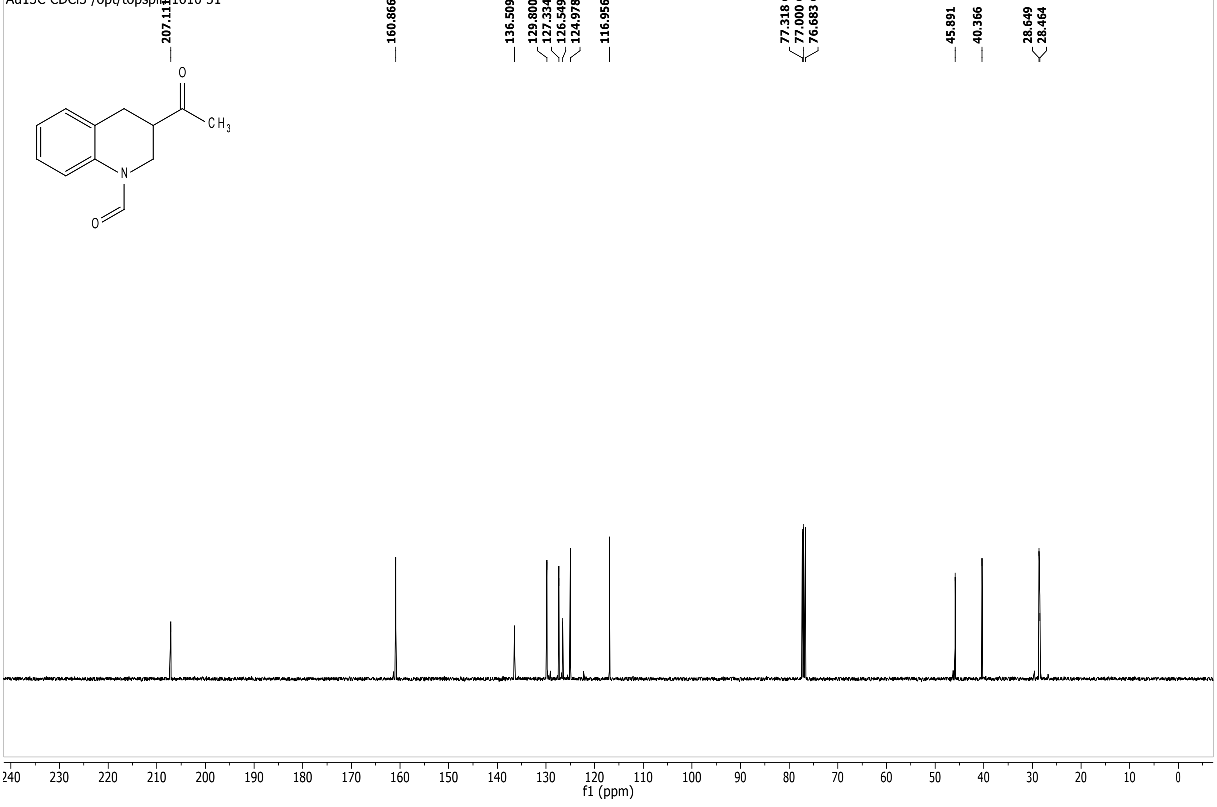

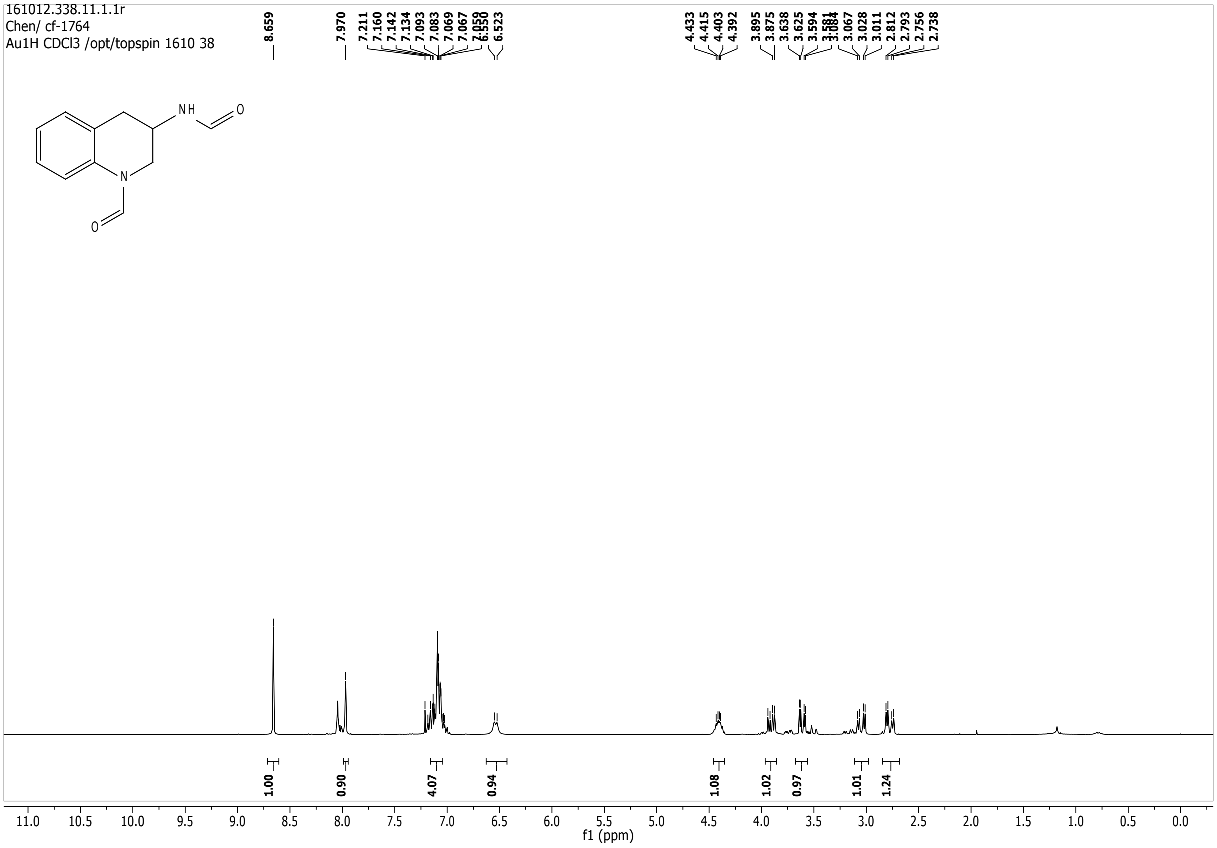
161012.338.11.1.1r
Chen/ cf-1764
Au1H CDCl3 /opt/topspin 1610 38
8.659
7.970
7.211
7.160
7.142
7.134
7.093
7.083
7.069
7.067
6.523
4.433
4.415
4.403
4.392
3.895
3.875
3.638
3.625
3.594
3.067
3.028
3.011
2.812
2.793
2.756
2.738
NH
O
N
O
1.00
0.90
4.07
0.94
1.08
1.02
0.97
1.01
1.24
11.0
10.5
10.0
9.5
9.0
8.5
8.0
7.5
7.0
6.5
6.0
5.5
5.0
4.5
4.0
3.5
3.0
2.5
2.0
1.5
1.0
0.5
0.0
f1 (ppm)

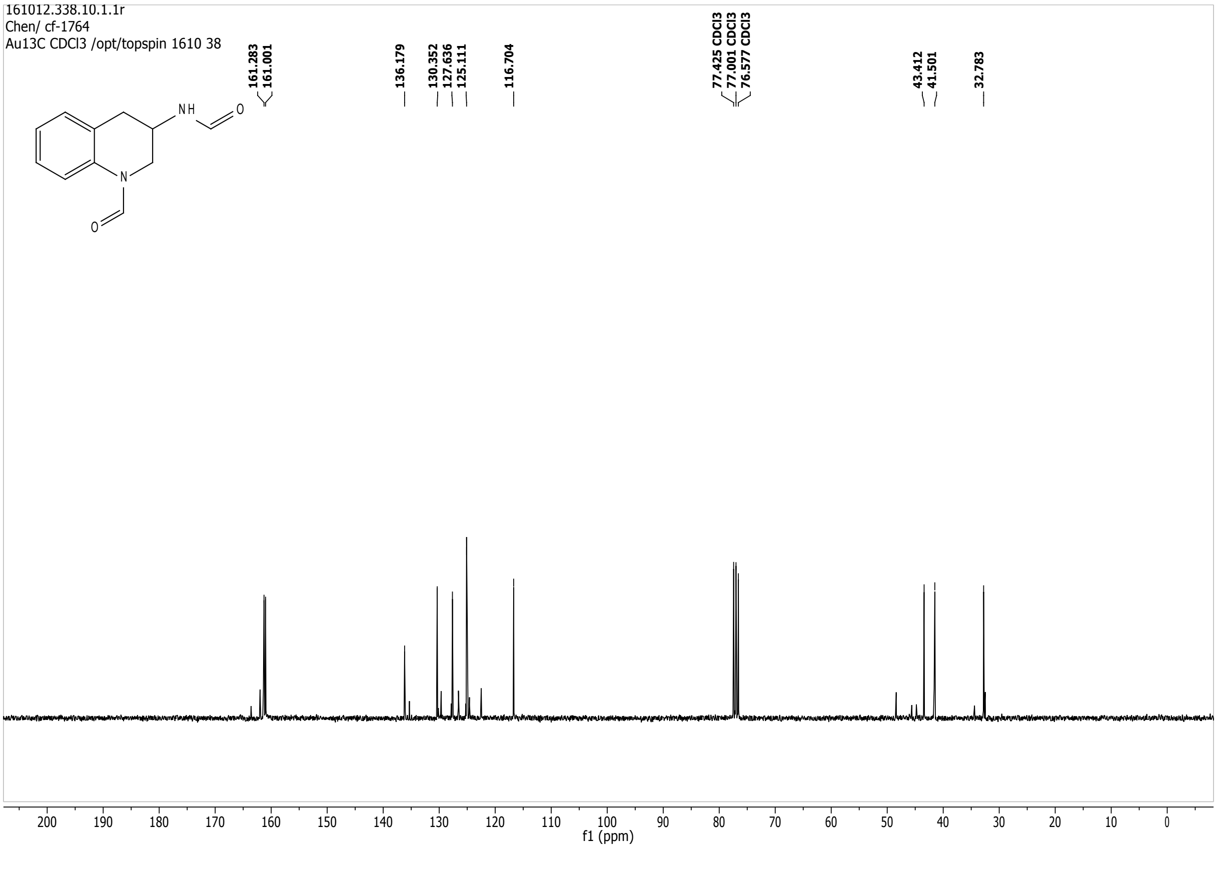
161012.338.10.1.1r
Chen/ cf-1764
Au13C CDCl3 /opt/topspin 1610 38
161.283
161.001
136.179
130.352
127.636
125.111
116.704
77.425 CDCl3
77.001 CDCl3
76.577 CDCl3
43.412
41.501
32.783
NH
O
N
O
200
190
180
170
160
150
140
130
120
110
100
90
80
70
60
50
40
30
20
10
0
f1 (ppm)

161004.f322.10.fid
Sahoo BS-345
PROTON CDCl3 {C:\Bruker\TopSpin3.2PL6} 1610 22

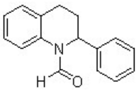

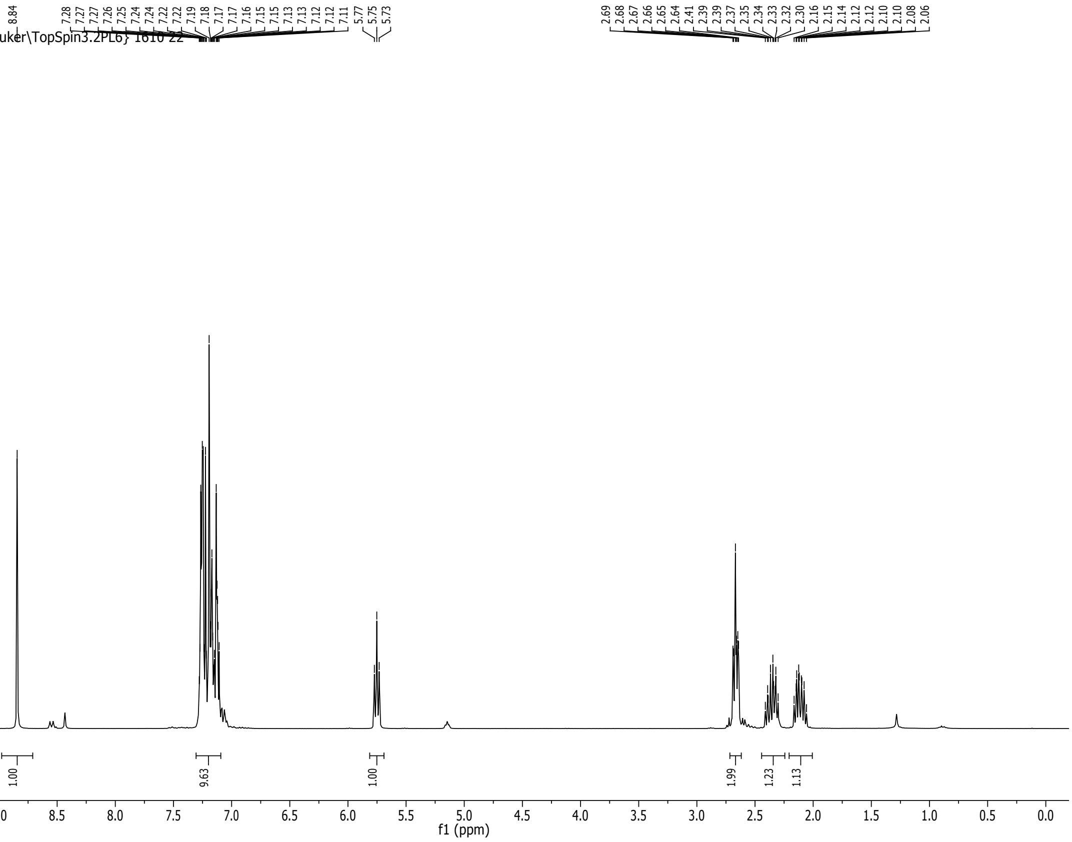

161004.f322.11.fid
Sahoo BS-345
C13CPD CDCl3 {C:\Bruker\TopSpin3.2PL6} 1610 22

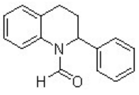

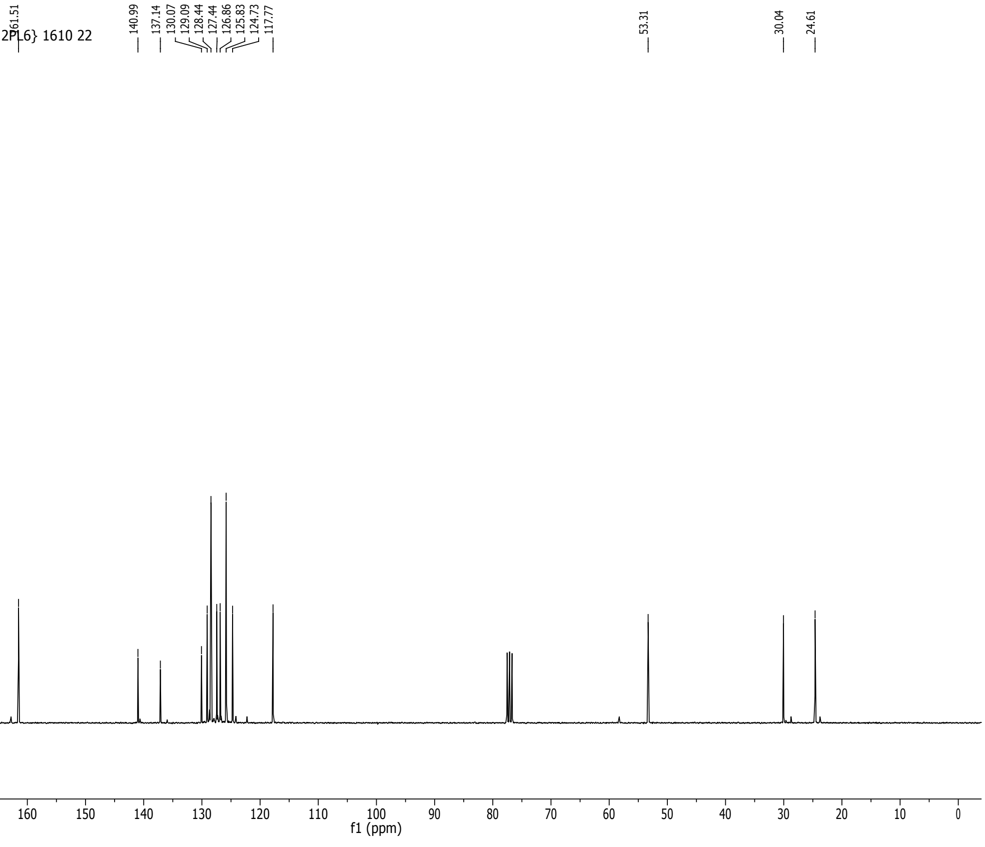

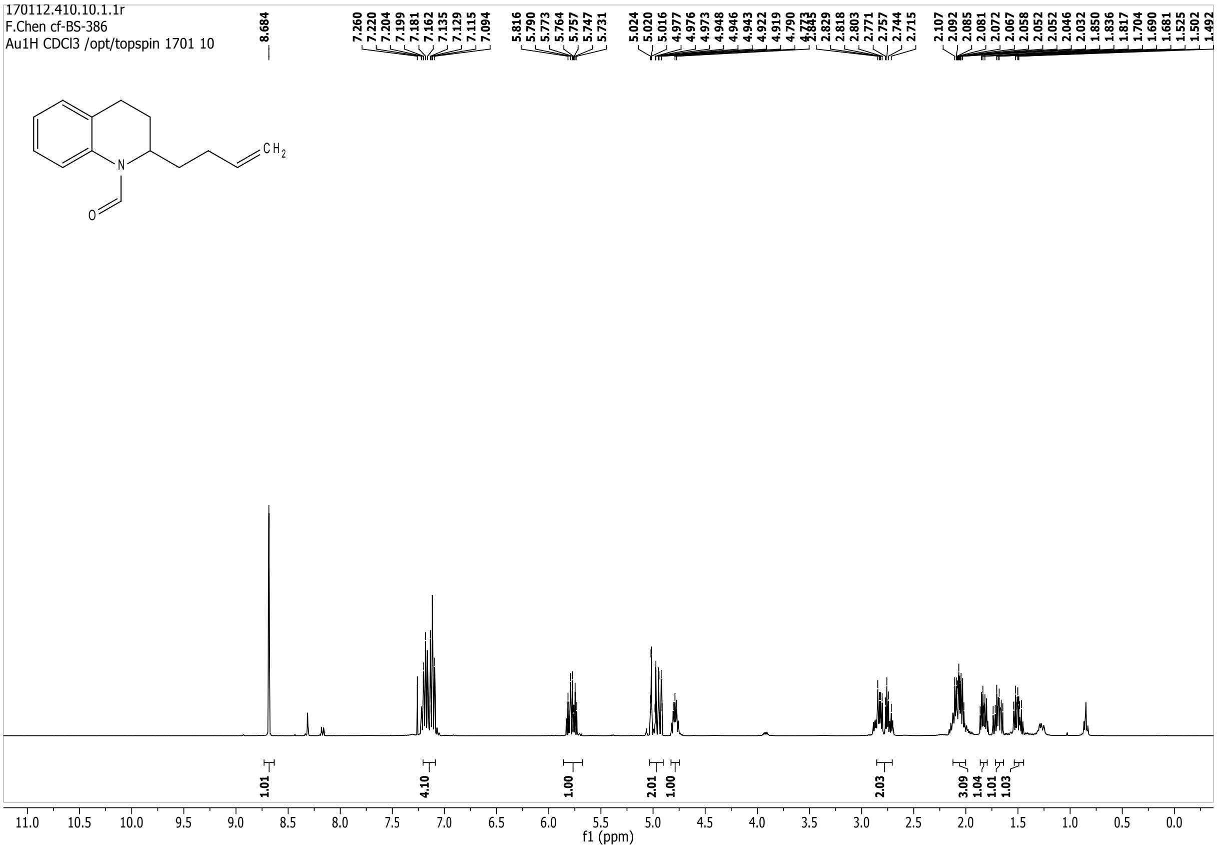
170112.410.10.1.1r
F.Chen cf-BS-386
Au1H CDCl3 /opt/topspin 1701 10
8.684
7.260 7.220 7.204 7.199 7.181 7.162 7.135 7.129 7.115 7.094
5.816 5.790 5.773 5.764 5.757 5.747 5.731
5.024 5.020 5.016 4.977 4.976 4.973 4.948 4.946 4.943 4.922 4.919 4.790
2.829 2.818 2.803 2.771 2.757 2.744 2.715
2.107 2.092 2.085 2.081 2.072 2.067 2.058 2.052 2.052 2.046 2.032 1.850 1.836 1.817 1.704 1.690 1.681 1.525 1.502 1.492
CH2
N
O
1.01
4.10
1.00
2.01
1.00
2.03
3.09
1.04
1.01
1.03
f1 (ppm)

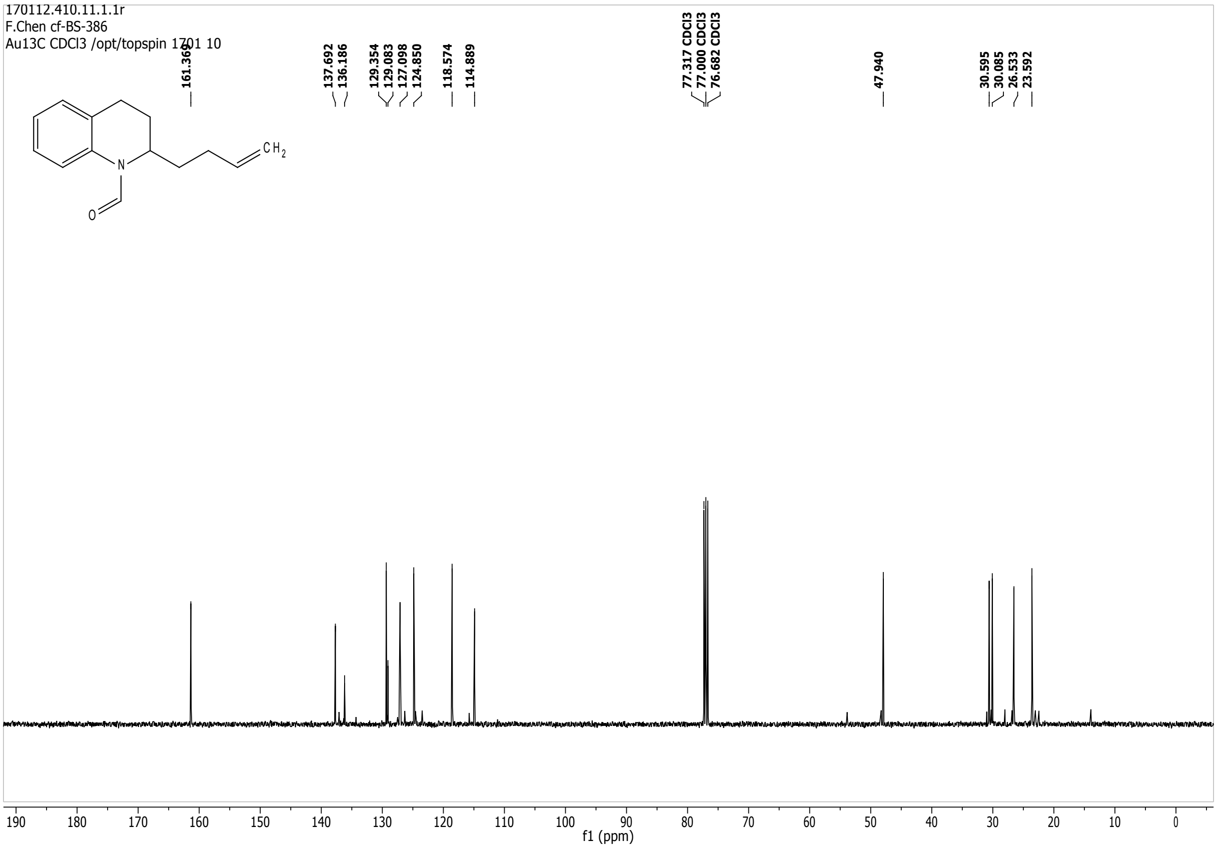
170112.410.11.1.1r
F.Chen cf-BS-386
Au13C CDCl3 /opt/topspin 1701 10
161.360
137.692 136.186
129.354 129.083 127.098 124.850
118.574 114.889
77.317 CDCl3
77.000 CDCl3
76.682 CDCl3
47.940
30.595 30.085 26.533 23.592
CH2
N
O
f1 (ppm)

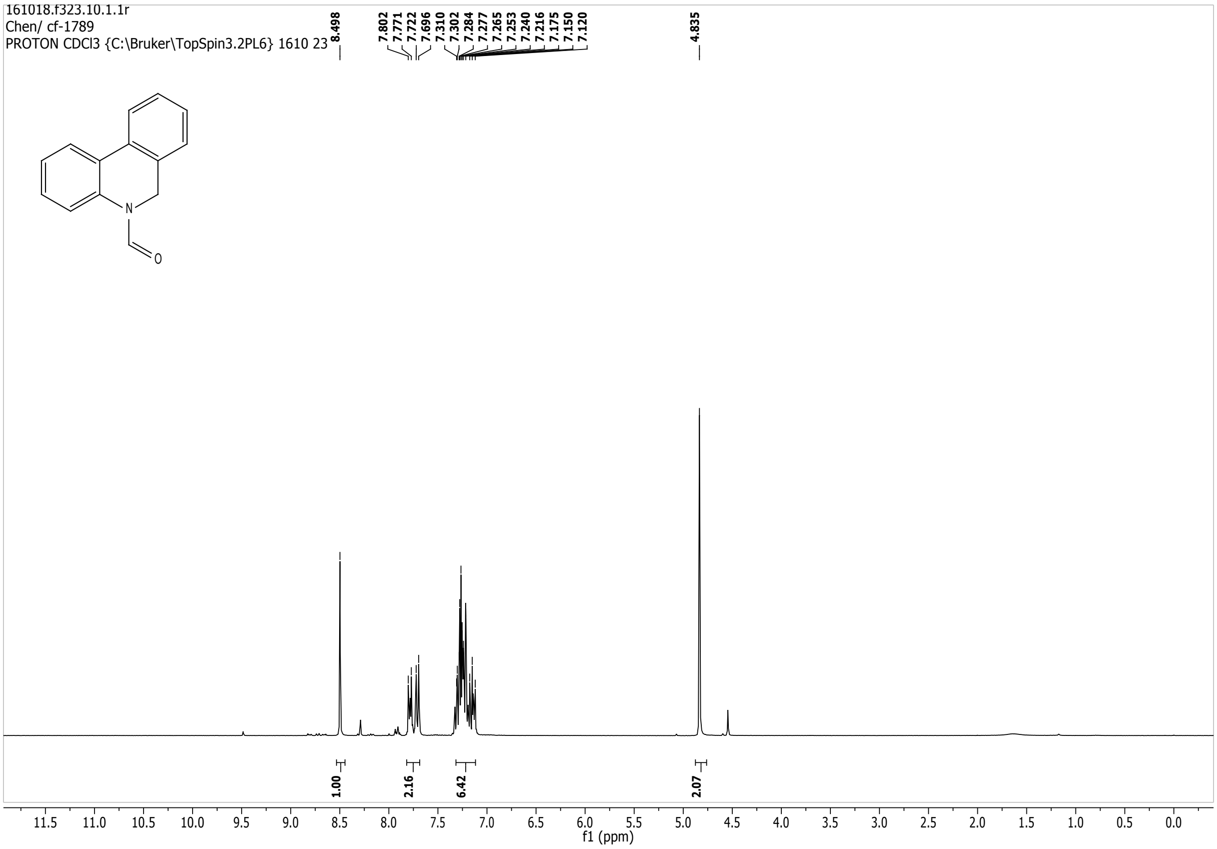
161018.f323.10.1.1r
Chen/ cf-1789
PROTON CDCl3 {C:\Bruker\TopSpin3.2PL6} 1610 23
8.498
7.802
7.771
7.722
7.696
7.310
7.302
7.284
7.277
7.265
7.253
7.240
7.216
7.175
7.150
7.120
4.835
N
O
1.00
2.16
6.42
2.07
f1 (ppm)

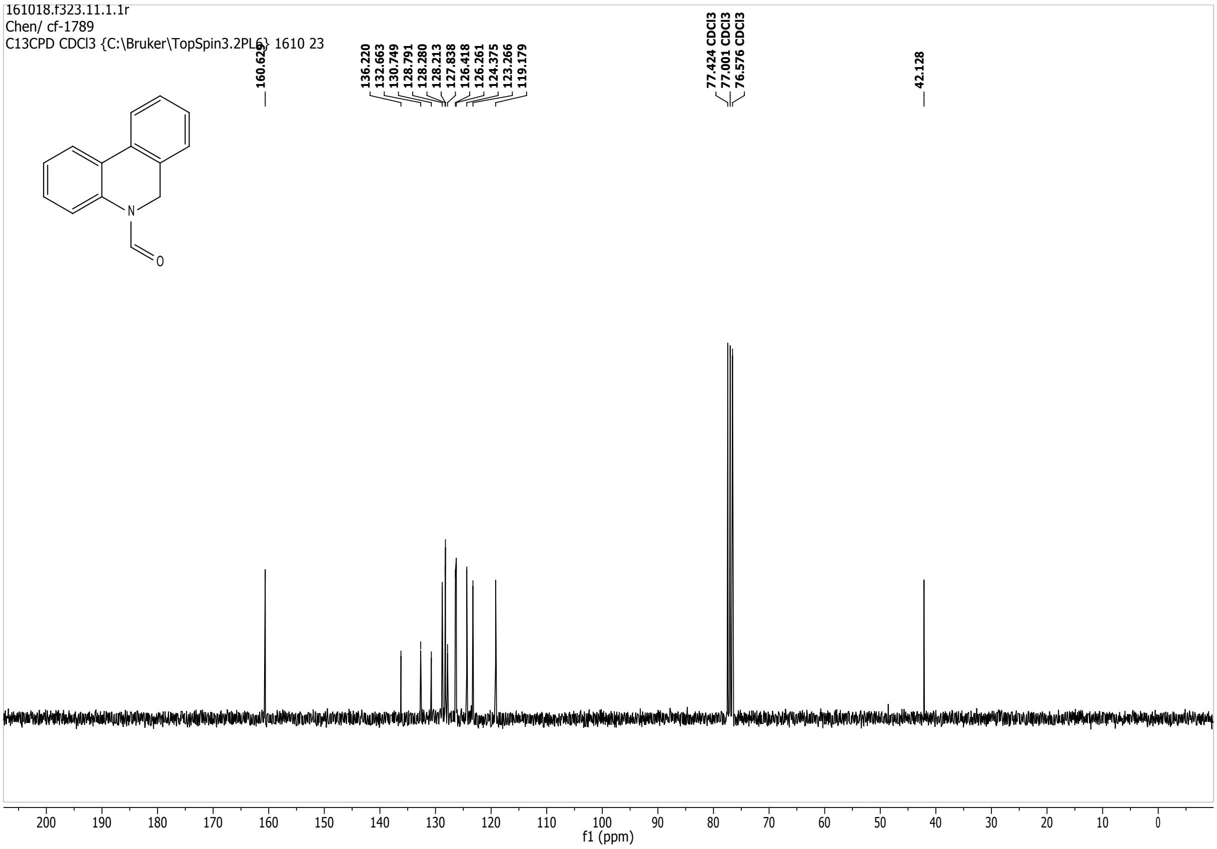
161018.f323.11.1.1r
Chen/ cf-1789
C13CPD CDCl3 {C:\Bruker\TopSpin3.2PL6} 1610 23
160.629
136.220
132.663
130.749
128.791
128.280
128.213
127.838
126.418
126.261
124.375
123.266
119.179
77.424 CDCl3
77.001 CDCl3
76.576 CDCl3
42.128
N
O
f1 (ppm)

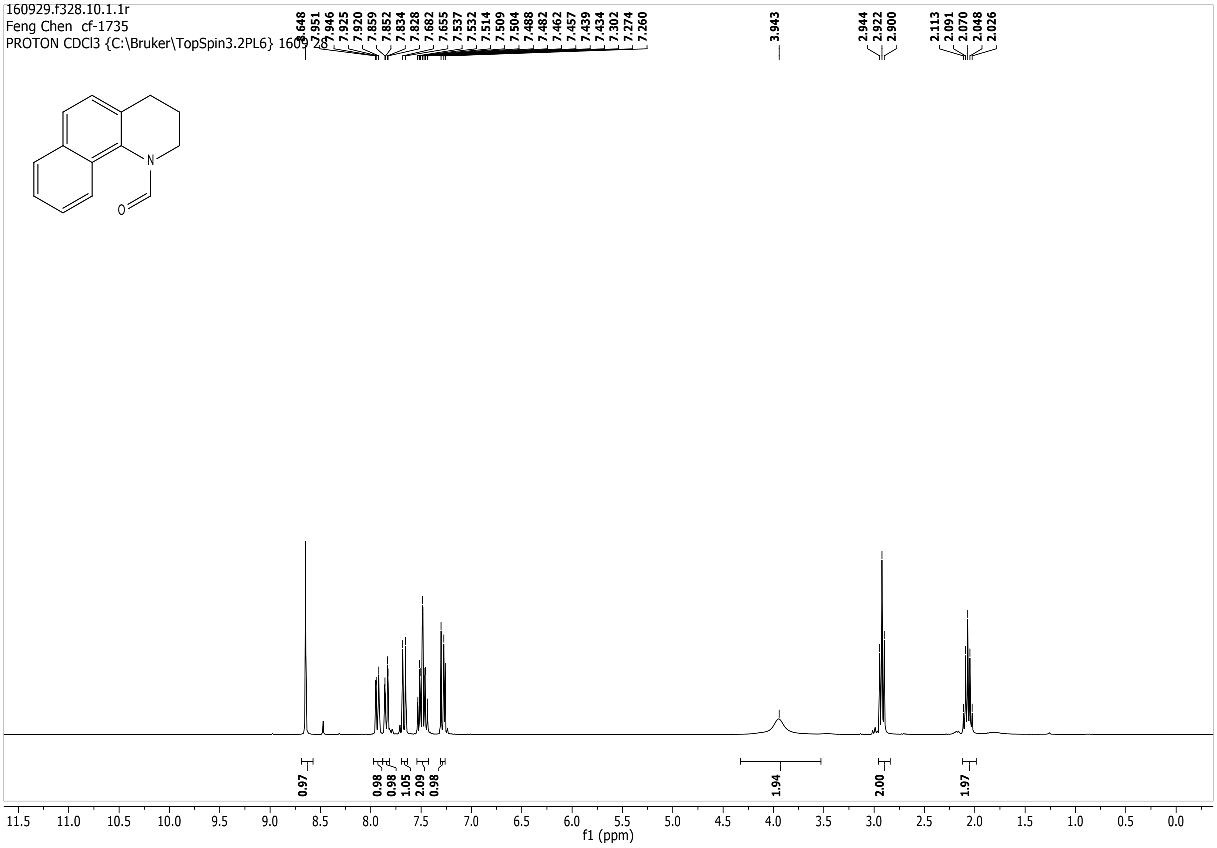
160929.f328.10.1.1r
Feng Chen cf-1735
PROTON CDCl3 {C:\Bruker\TopSpin3.2PL6} 1609 28
8.648
7.951
7.946
7.925
7.920
7.859
7.852
7.834
7.828
7.682
7.655
7.537
7.532
7.514
7.509
7.504
7.488
7.482
7.462
7.457
7.439
7.434
7.302
7.274
7.260
3.943
2.944
2.922
2.900
2.113
2.091
2.070
2.048
2.026
N
O
0.97
0.98
0.98
1.05
2.09
0.98
1.94
2.00
1.97
11.5 11.0 10.5 10.0 9.5 9.0 8.5 8.0 7.5 7.0 6.5 6.0 5.5 5.0 4.5 4.0 3.5 3.0 2.5 2.0 1.5 1.0 0.5 0.0
f1 (ppm)

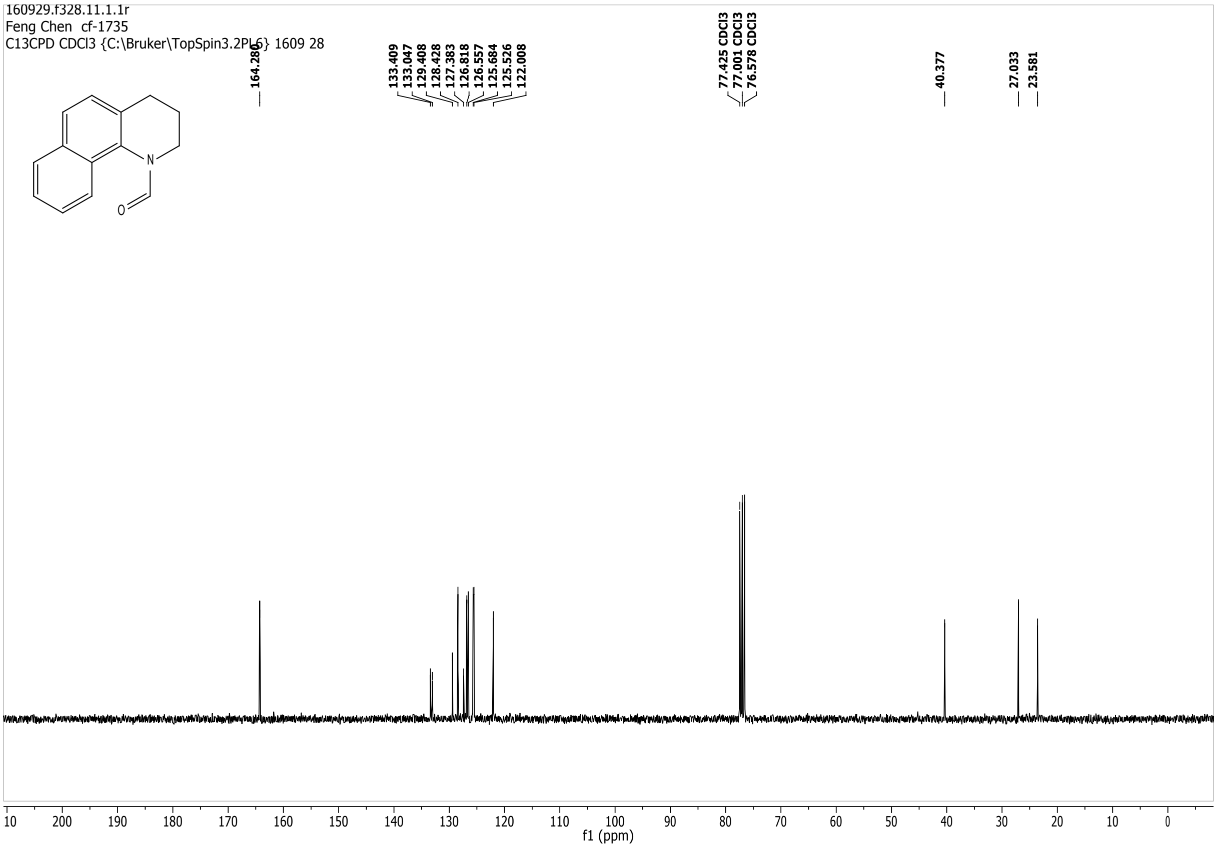
160929.f328.11.1.1r
Feng Chen cf-1735
C13CPD CDCl3 {C:\Bruker\TopSpin3.2PL6} 1609 28
164.286
133.409
133.047
129.408
128.428
127.383
126.818
126.557
125.684
125.526
122.008
77.425 CDCl3
77.001 CDCl3
76.578 CDCl3
40.377
27.033
23.581
N
O
10 200 190 180 170 160 150 140 130 120 110 100 90 80 70 60 50 40 30 20 10 0
f1 (ppm)

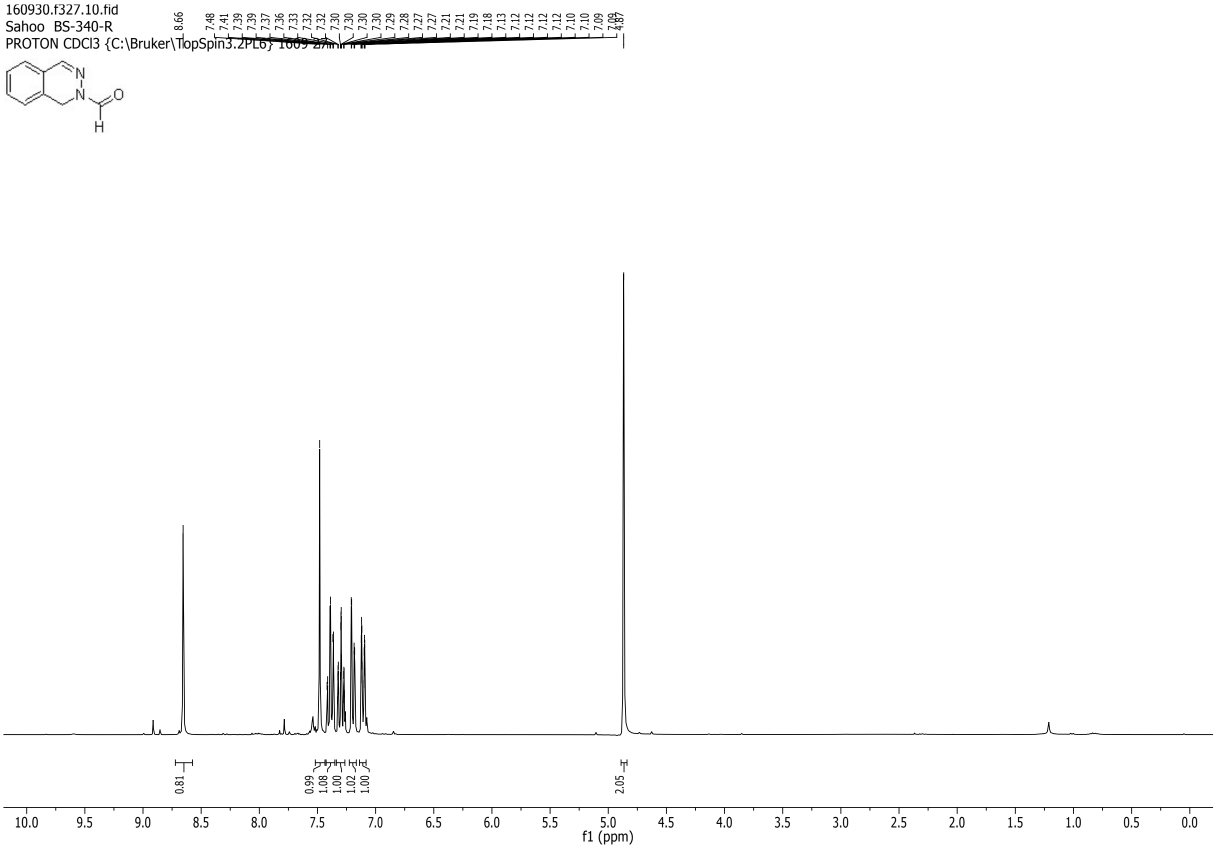
160930.f327.10.fid
Sahoo BS-340-R
PROTON CDCl3 {C:\Bruker\TopSpin3.2PL6} 1609 27
8.66
7.48
7.41
7.39
7.39
7.37
7.36
7.33
7.32
7.32
7.30
7.30
7.30
7.30
7.29
7.28
7.27
7.27
7.21
7.21
7.19
7.18
7.13
7.12
7.12
7.12
7.12
7.10
7.10
7.09
4.87
0.81
0.99
1.08
1.00
1.02
1.00
2.05
f1 (ppm)

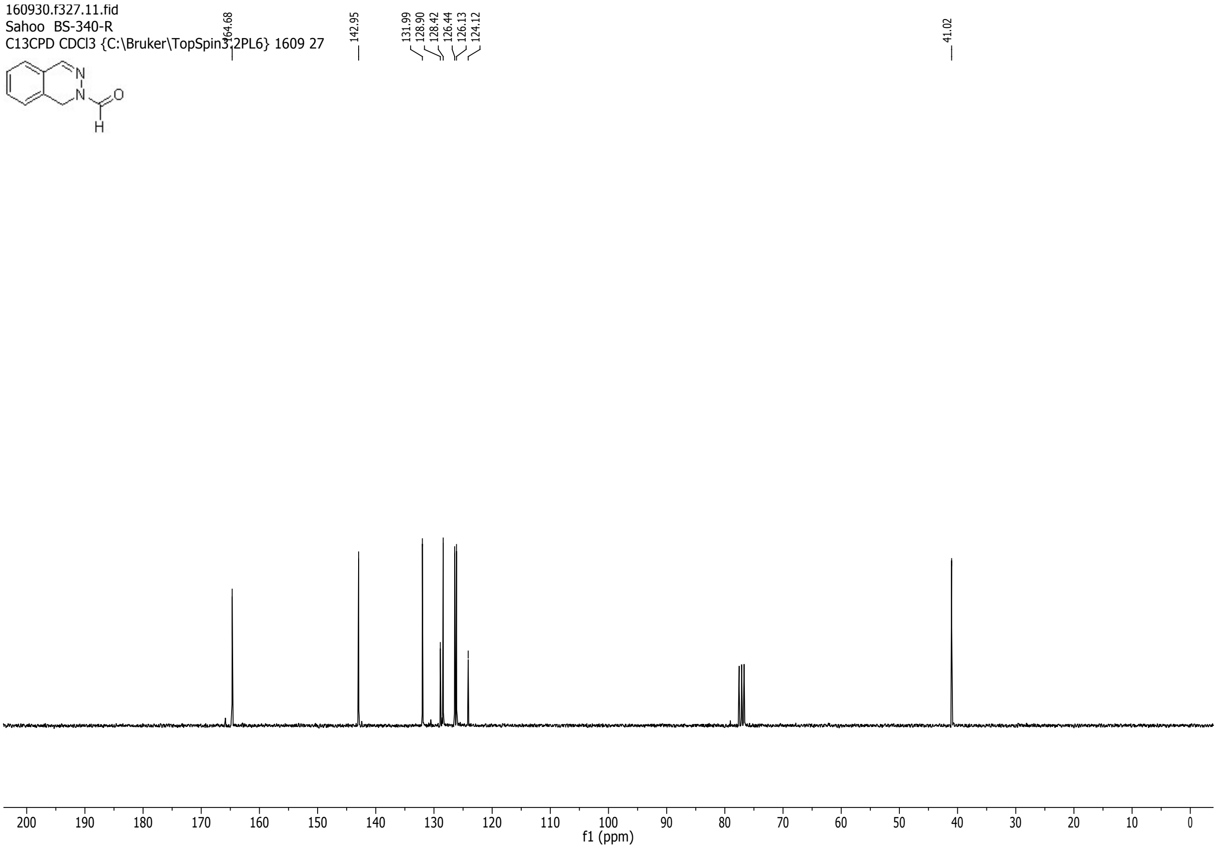
160930.f327.11.fid
Sahoo BS-340-R
C13CPD CDCl3 {C:\Bruker\TopSpin3.2PL6} 1609 27
164.68
142.95
131.99
128.90
128.42
126.44
126.13
124.12
41.02
f1 (ppm)

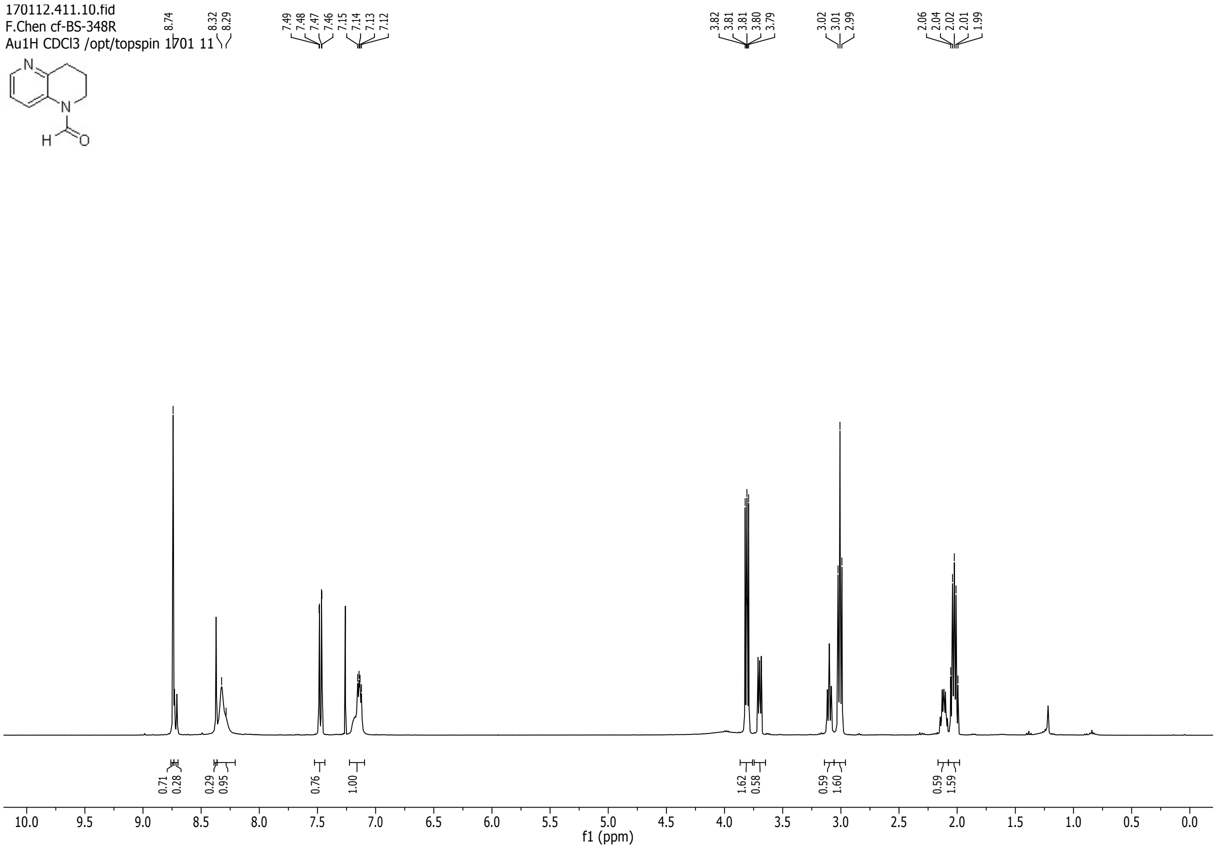
170112.411.10.fid
F.Chen cf-BS-348R
Au1H CDCl3 /opt/topspin 1701 11
N
N
H
O
8.74
8.32
8.29
7.49
7.48
7.47
7.46
7.15
7.14
7.13
7.12
3.82
3.81
3.81
3.80
3.79
3.02
3.01
2.99
2.06
2.04
2.02
2.01
1.99
0.71
0.28
0.29
0.95
0.76
1.00
1.62
0.58
0.59
1.60
0.59
1.59
10.0 9.5 9.0 8.5 8.0 7.5 7.0 6.5 6.0 5.5 5.0 4.5 4.0 3.5 3.0 2.5 2.0 1.5 1.0 0.5 0.0
f1 (ppm)

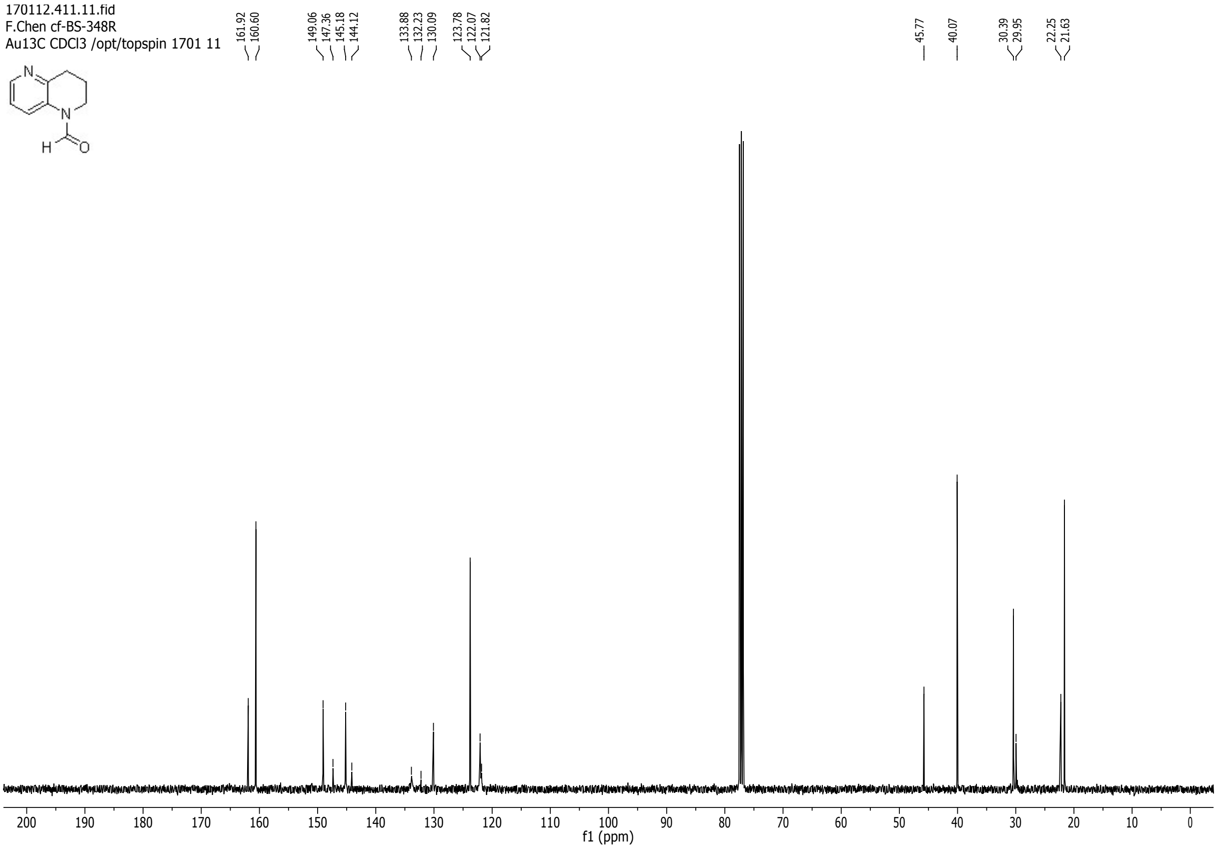
170112.411.11.fid
F.Chen cf-BS-348R
Au13C CDCl3 /opt/topspin 1701 11
N
N
H
O
161.92
160.60
149.06
147.36
145.18
144.12
133.88
132.23
130.09
123.78
122.07
121.82
45.77
40.07
30.39
29.95
22.25
21.63
200 190 180 170 160 150 140 130 120 110 100 90 80 70 60 50 40 30 20 10 0
f1 (ppm)

11. References:

1. J. Wu, C. Wang, W. Tang, A. Pettman and J. Xiao, *Chem. Eur. J.* **2012**, *18*, 9525-9529.
